# Supplementary material for: Investigation of gene-gene interactions in cardiac traits and serum fatty acid levels in the LURIC Health Study
Source: PLoS One. 2020 Sep 11;15(9):e0238304. doi: 10.1371/journal.pone.0238304 (PMC7485803; doi:10.1371/journal.pone.0238304)
Supplement: S1 Fig — In the Manhattan plots, the blue line represents the genome wide significance (-log10 (5×10^−8^) = 7.30) and the red line represents the suggestive Bonferroni significance (-log10 (0.05/577007) = 7.06). Seven significant associations (at genome wide significance) between SNPs (rs174548, rs174549, rs4246215, rs174577, rs174583, rs174547, and rs174534) and log transformed Dihomo_g_Linolenic_C20_3n6 were identified. Two significant associations (at or close to the suggestive Bonferroni significance) were identified between rs11744802 and log transformed C18_2n6tt and between rs174577 and Arachidonic_acid_C20_4n6. (PDF) [file pone.0238304.s001.pdf]

S1 Fig. GWAS results of the Manhattan plots and the QQ plots for each phenotype. In the Manhattan plots, the red line represents the genome wide significance ($-\log_{10}(5\times10^{-8}) = 7.30$) and the blue line represents the suggestive Bonferroni significance ($-\log_{10}(0.05/577007) = 7.06$). Seven significant associations (at genome wide significance) between SNPs (rs174548, rs174549, rs4246215, rs174577, rs174583, rs174547, and rs174534) and log transformed Dihomo_g_Linolenic_C20_3n6 were identified. Two significant associations (at or close to the suggestive Bonferroni significance) were identified between rs11744802 and log transformed C18_2n6tt and between rs174577 and Arachidonic_acid_C20_4n6.

A. Directory for the plots:

Cardiac traits	Description	Manhattan Plot	QQ plot	Fatty Acids	Description	Manhattan Plot	QQ plot
**afibtyp**	Arrhythmia (1=chronic,2=intermit.)	B	C	**Palmitic_acid_C16_0**	Saturated fatty acid	FF	GG
**afibyn**	Atrial fibrillation (yes/no)	D	E	**Stearic_acid_C18_0**	Saturated fatty acid	HH	II
**cadyn**	Coronary artery disease (>10% (=20% or more)) or clinical(y/n)	F	G	**DHA_C22_6n3**	polyunsaturated omega-3 fatty acid	JJ	KK
**canceryn**	Cancer disease	H	I	**LOG_Myristic_acid_C14_0**	Log-transformed measurement in saturated fatty acid	LL	MM
**cmpyn**	Cardiomyopathy (yes/no)	J	K	**LOG_Trans_Palmitoleic_acid_C16_1n7t**	Log-transformed measurement in exogenous monosaturated fatty acid, trans-palmitoleic acid (C16:1n7t)	NN	OO
**death2010**	Yes/No	L	M	**LOG_Oleic_acid_C18_1n9**	Log-transformed measurement in monounsaturated omega-9 fatty acid	PP	QQ
**diabetes2010**	Diabetes (definition including hba1c > = 6.5)	N	O	**LOG_C18_2n6tt**	Log-transformed measurement in trans-fatty acid isomer of C18:2n6, 9-trans 12-trans octadecanoic acid, linolelaidic acid	RR	SS
**dm2yn**	Type II diabetes mellitus	P	Q	**LOG_C18_2n6ct**	Log-transformed measurement in trans-fatty acid isomer of C18:2n6, 9-cis 12-trans octadecanoic acid	TT	UU
**hyptenyn**	History of arterial hypertension	R	S	**LOG_C18_2n6tc**	Log-transformed measurement in trans-fatty acid isomer of C18:2n6, 9-trans 12-cis octadecanoic acid	VV	WW
**insuthyn**	Treatment with insulin	T	U	**LOG_Linoleic_acid_C18_2n6**	Log-transformed measurement in polyunsaturated omega-6 fatty acid	XX	YY
**pvdyn**	Peripheral vascular disease	V	W	**LOG_a_Linolenic_acid_C18_3n3**	Log-transformed measurement in polyunsaturated omega-3 fatty acid	ZZ	AAA
**rhythyn**	Peripheral vascular disease	X	Y	**Arachidonic_acid_C20_4n6**	Polyunsaturated omega-6 fatty acid	BBB	CCC
**strokeyn**	Arrhythmia	Z	AA	**LOG_Dihomo_g_Linolenic_C20_3n6**	Log-transformed measurement in polyunsaturated omega-6 fatty acid	DDD	EEE
**vdyn**	Stroke/PRIND/TIA	BB	CC	**LOG_EP A_C20_5n3**	Log-transformed measurement in polyunsaturated omega-3 fatty acid	FFF	GGG
**venthrom**	Valve disease (yes/no)	DD	EE				

Directory

B

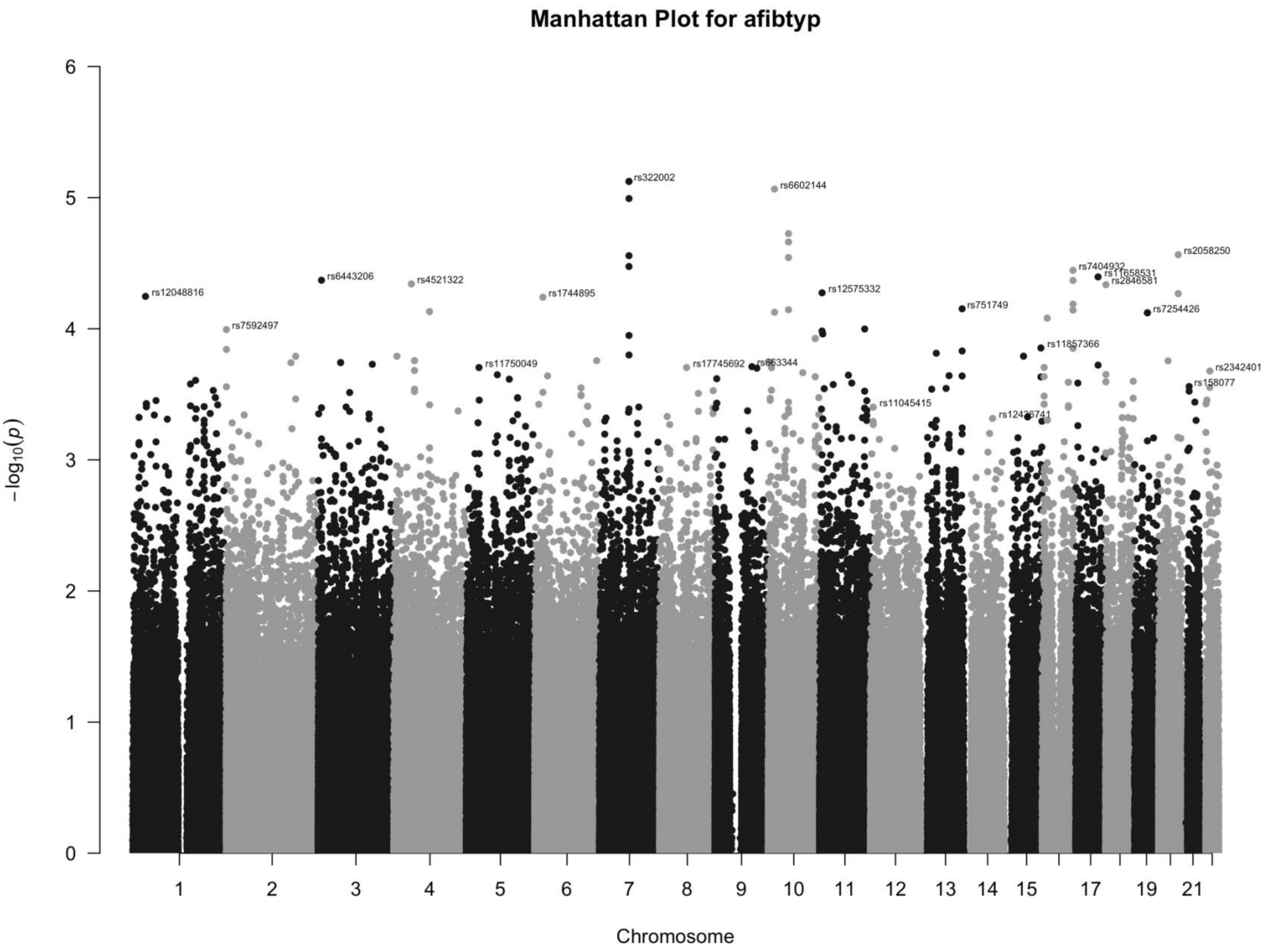

Manhattan Plot for afibtyp
6
5
4
3
2
1
0
−log10(p)
rs322002
rs6602144
rs2058250
rs7404932
rs11658531
rs2846581
rs12048816
rs6443206
rs4521322
rs1744895
rs12575332
rs751749
rs7254426
rs7592497
rs11857366
rs11750049
rs17745692
rs663344
rs2342401
rs158077
rs11045415
rs12436741
1
2
3
4
5
6
7
8
9
10
11
12
13
14
15
17
19
21
Chromosome

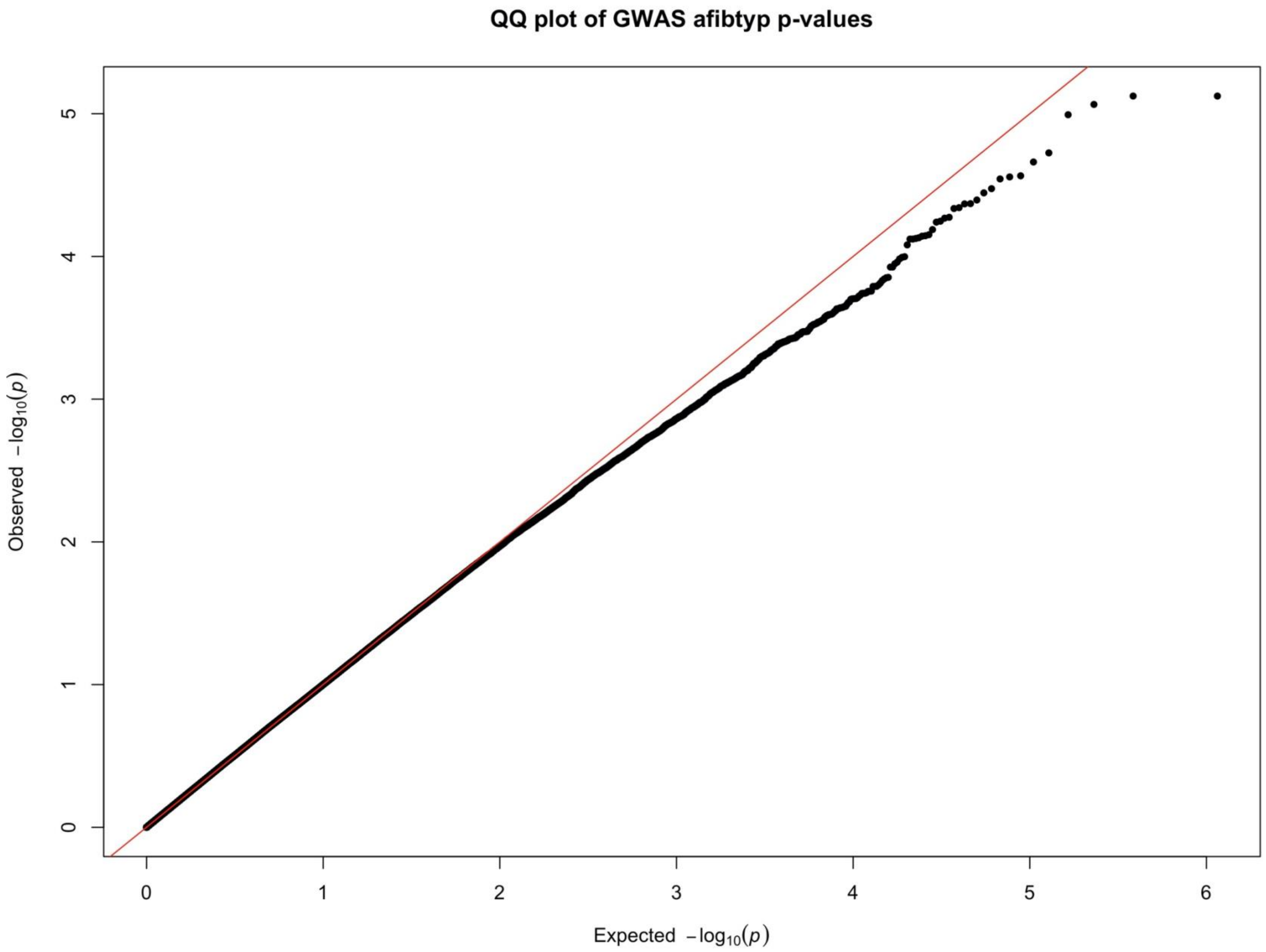
QQ plot of GWAS afibtyp p-values
Observed −log10(p)
Expected −log10(p)
0
1
2
3
4
5
6

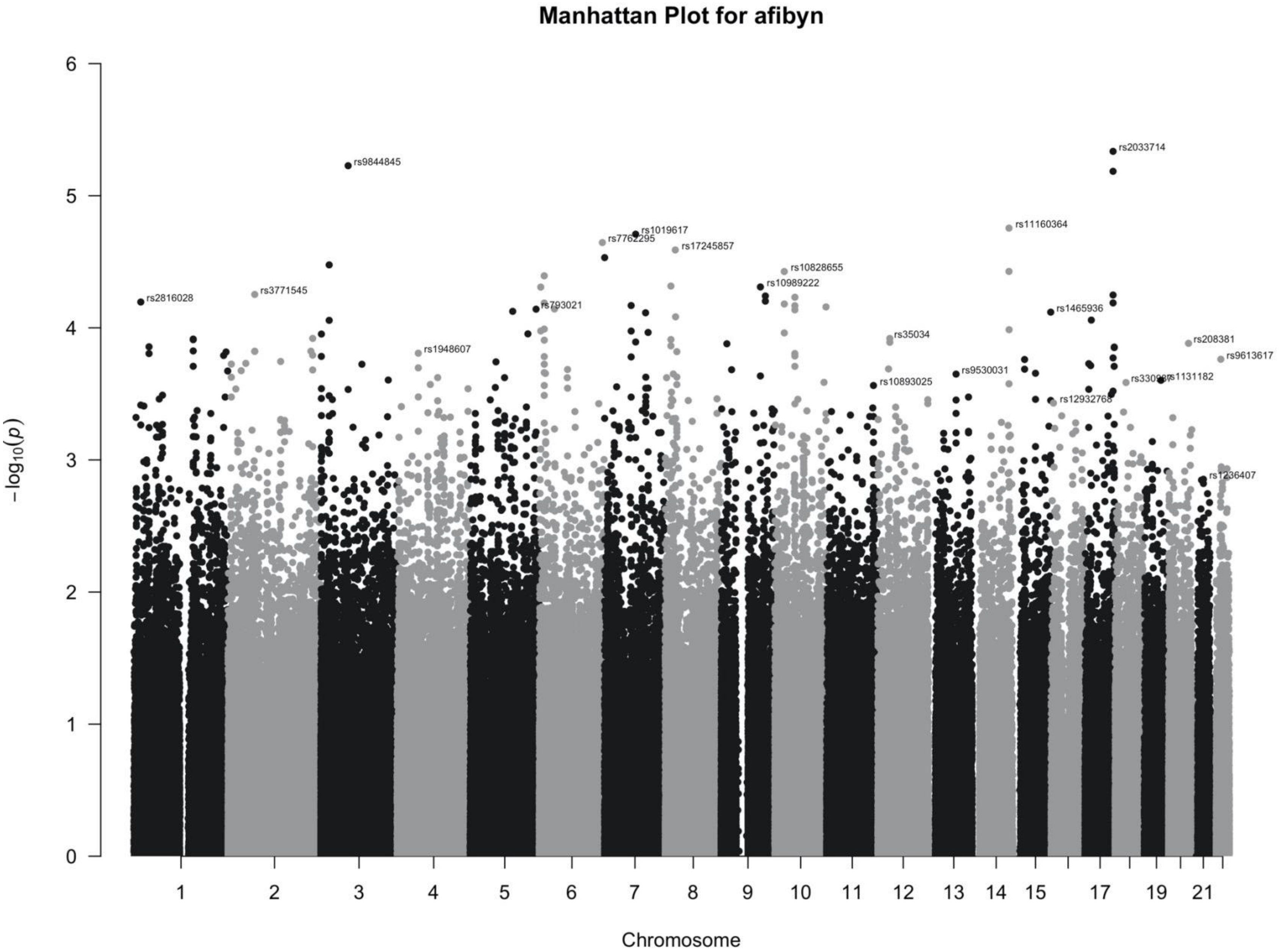
Manhattan Plot for afibyn
−log10(p)
Chromosome
rs9844845
rs2033714
rs1019617
rs7762295
rs17245857
rs11160364
rs10828655
rs10989222
rs3771545
rs2816028
rs793021
rs1465936
rs35034
rs208381
rs1948607
rs9613617
rs9530031
rs1131182
rs10893025
rs12932768
rs1236407

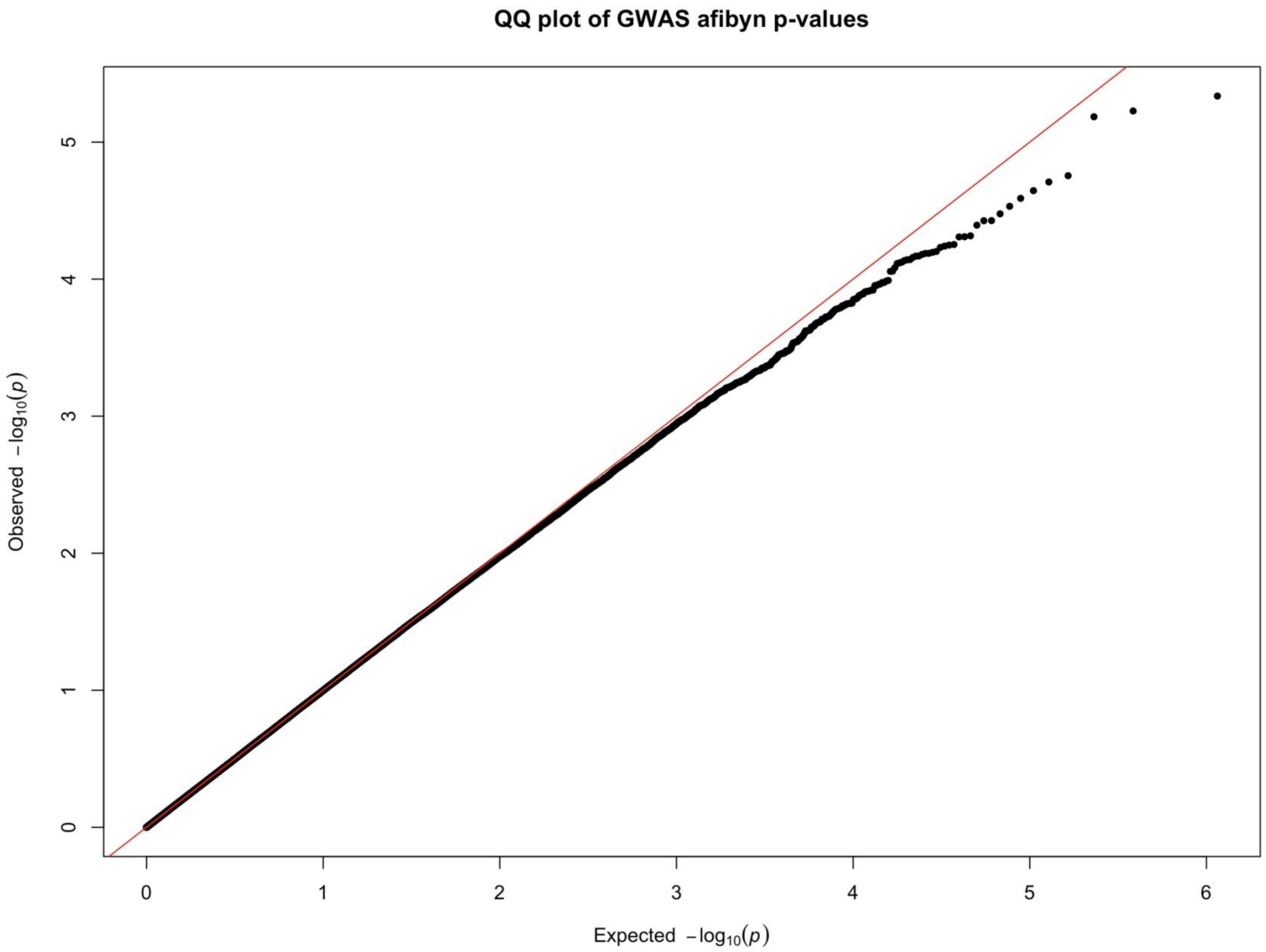
QQ plot of GWAS afibyn p-values
Observed −log10(p)
Expected −log10(p)
0
1
2
3
4
5
6

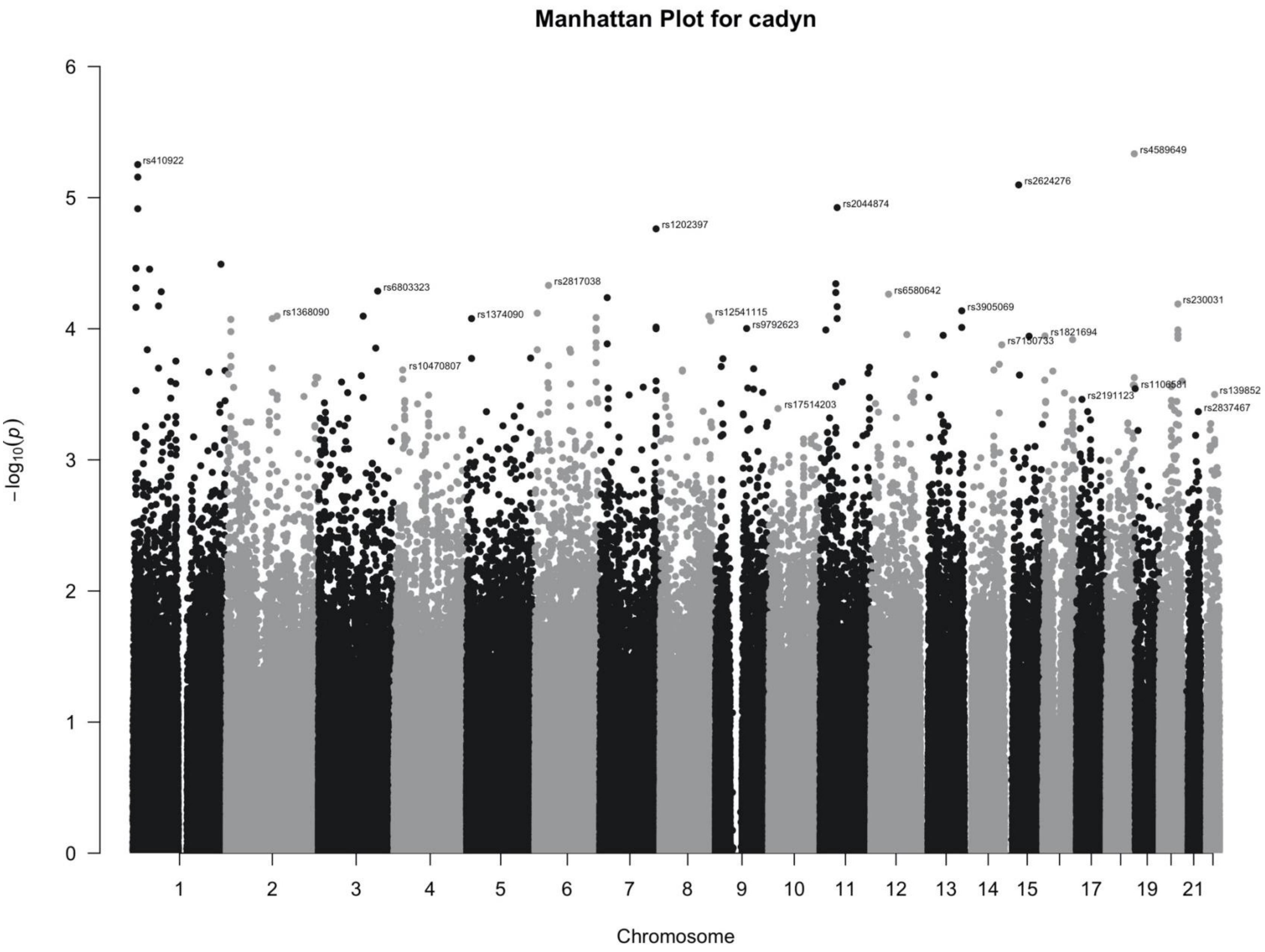
Manhattan Plot for cadyn
−log10(p)
0
1
2
3
4
5
6
Chromosome
1
2
3
4
5
6
7
8
9
10
11
12
13
14
15
17
19
21
rs410922
rs1368090
rs6803323
rs10470807
rs1374090
rs2817038
rs1202397
rs12541115
rs9792623
rs17514203
rs2044874
rs6580642
rs3905069
rs1821694
rs2624276
rs2191123
rs4589649
rs230031
rs139852
rs2837467

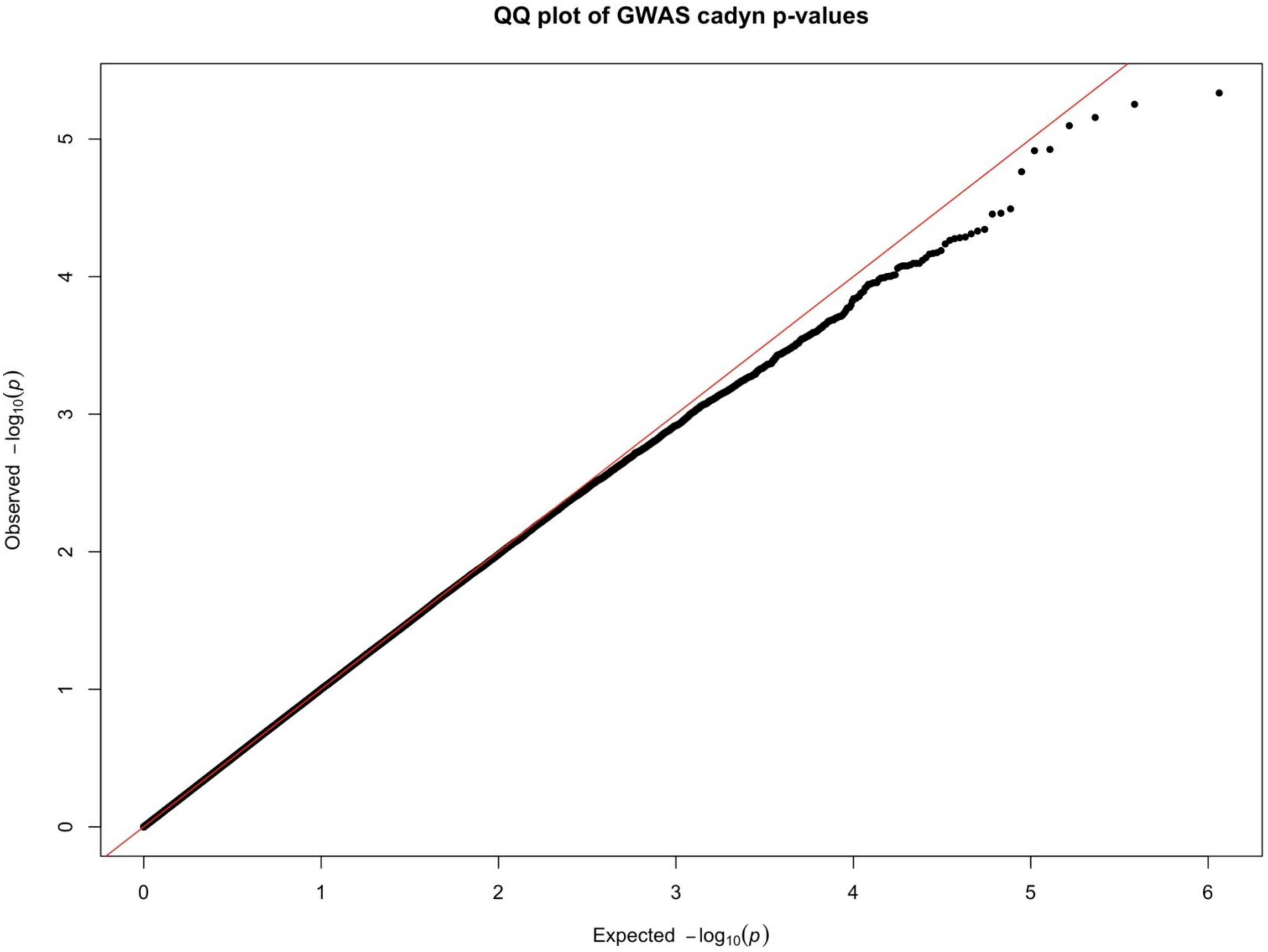
QQ plot of GWAS cadyn p-values
Observed $-\log_{10}(p)$
Expected $-\log_{10}(p)$
0
1
2
3
4
5
6

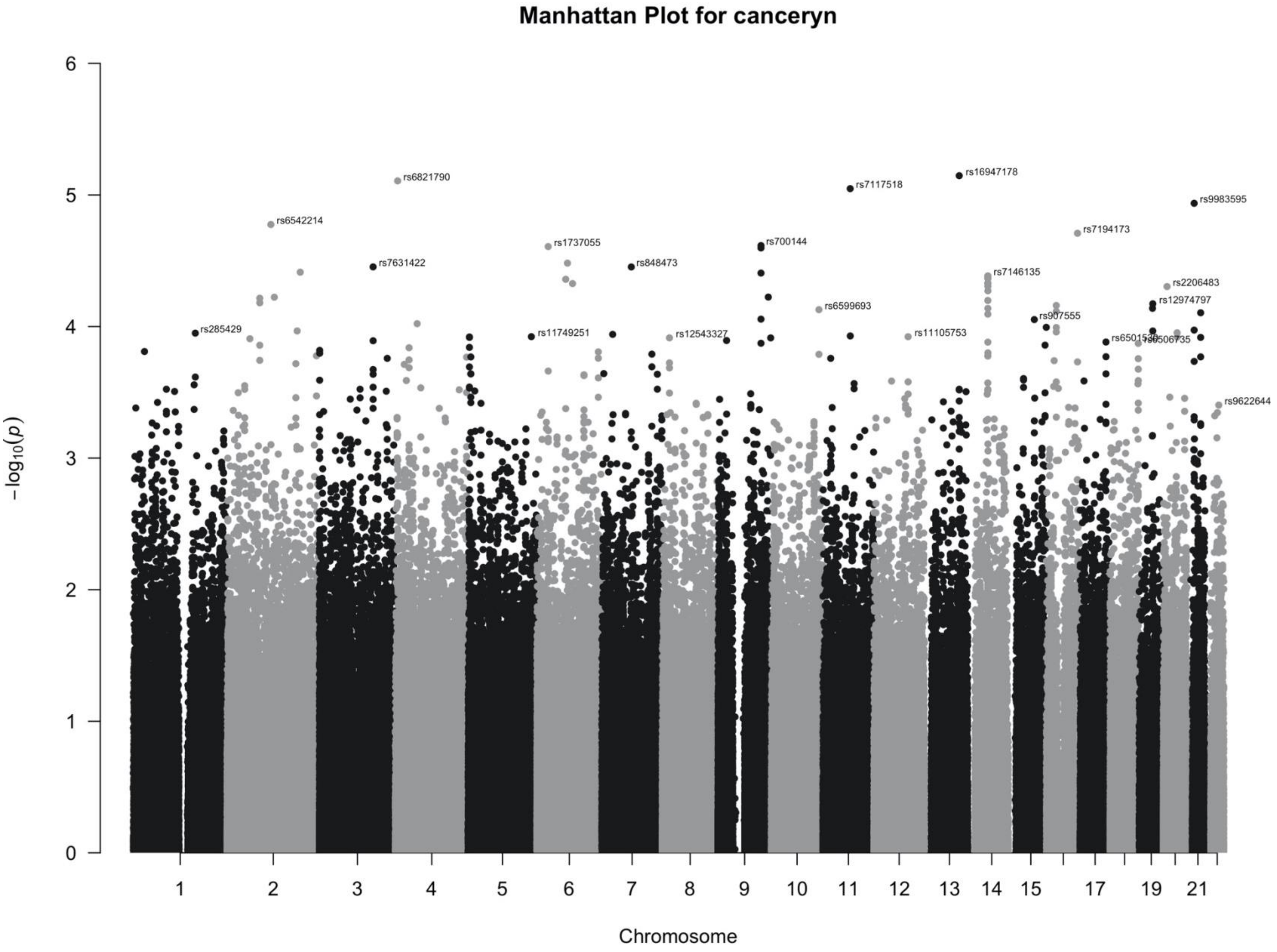
Manhattan Plot for canceryn
−log10(p)
Chromosome
0
1
2
3
4
5
6
1 2 3 4 5 6 7 8 9 10 11 12 13 14 15 17 19 21
rs6821790
rs16947178
rs7117518
rs9983595
rs6542214
rs7194173
rs1737055
rs700144
rs7631422
rs848473
rs7146135
rs2206483
rs6599693
rs12974797
rs907555
rs285429
rs11749251
rs12543327
rs11105753
rs9622644

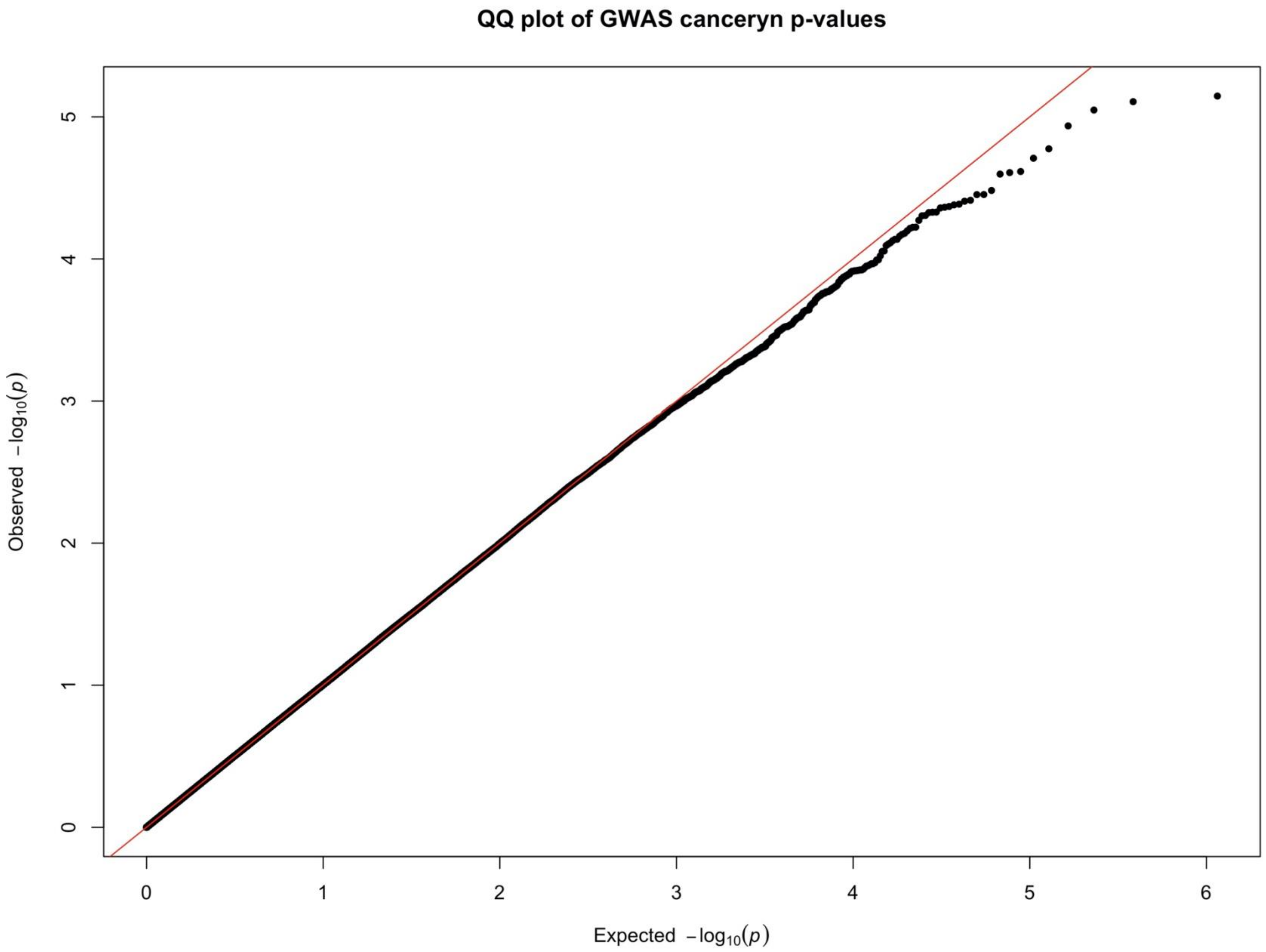
QQ plot of GWAS canceryn p-values
Observed −log10(p)
Expected −log10(p)
0
1
2
3
4
5
6

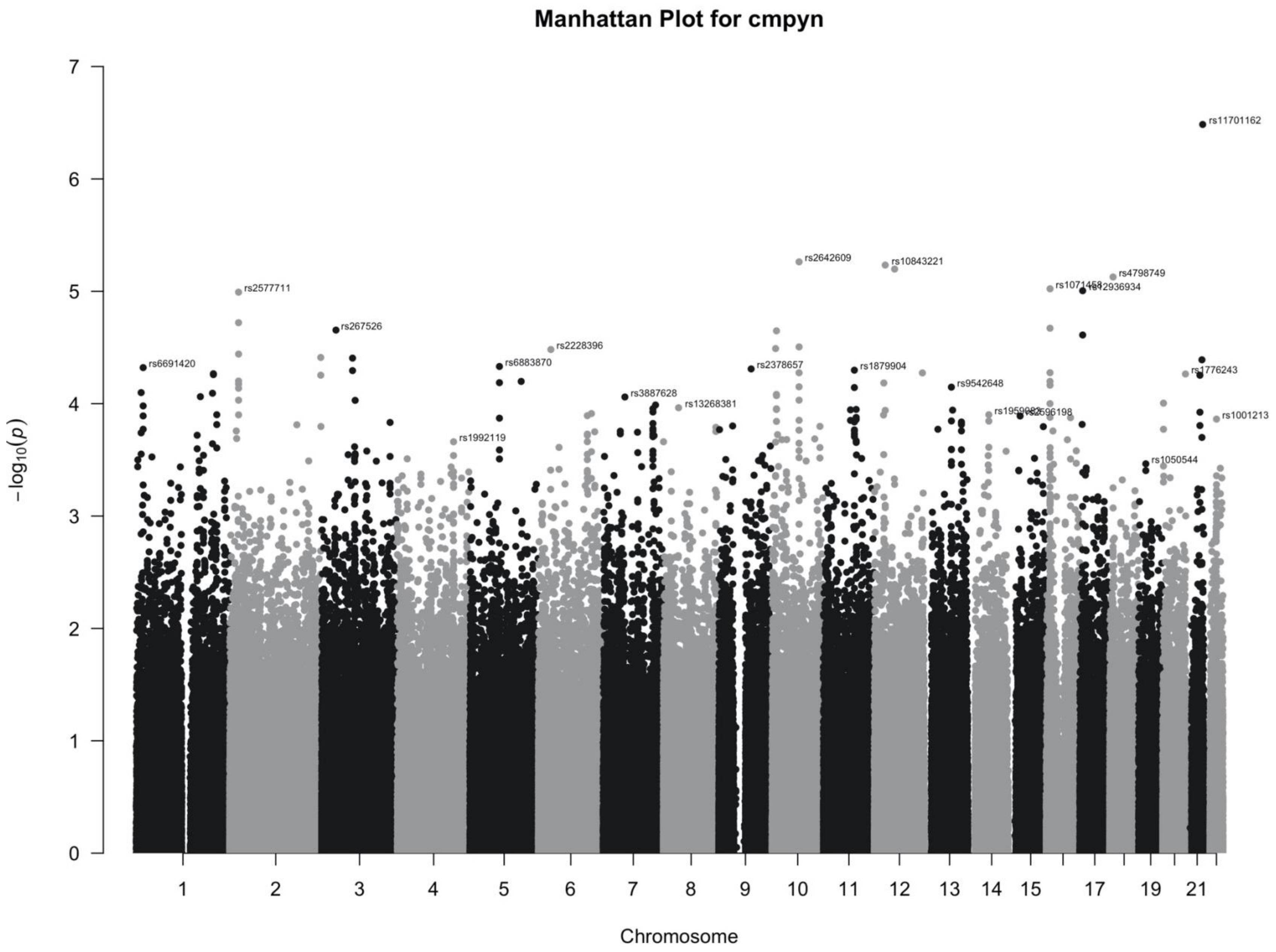
Manhattan Plot for cmpyn
−log10(p)
0
1
2
3
4
5
6
7
Chromosome
1
2
3
4
5
6
7
8
9
10
11
12
13
14
15
17
19
21
rs11701162
rs2642609
rs10843221
rs4798749
rs2577711
rs267526
rs2228396
rs6691420
rs6883870
rs2378657
rs1879904
rs1776243
rs9542648
rs3887628
rs13268381
rs1001213
rs1992119
rs1050544

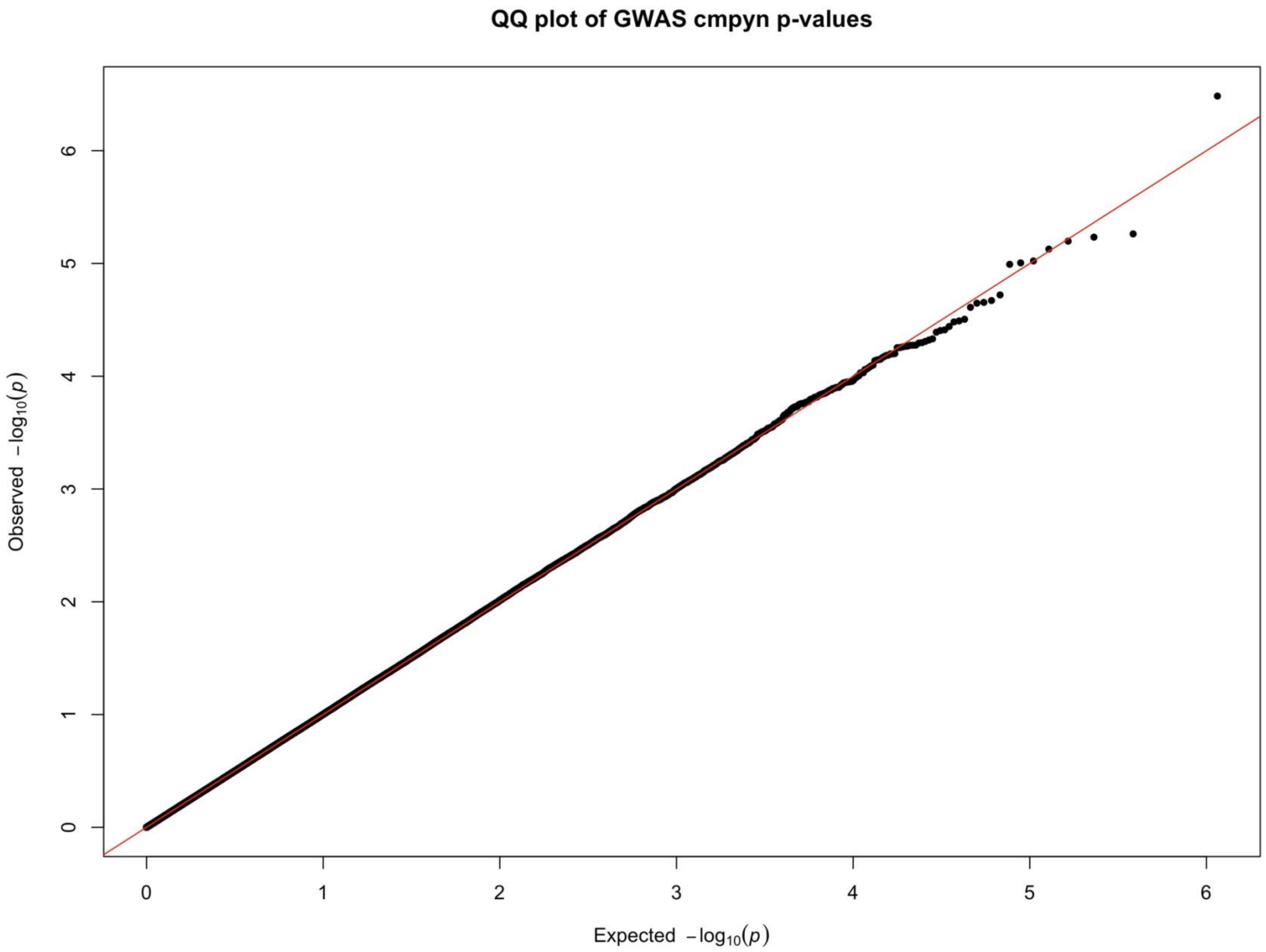
QQ plot of GWAS cmpyn p-values
Observed $-\log_{10}(p)$
Expected $-\log_{10}(p)$
0
1
2
3
4
5
6

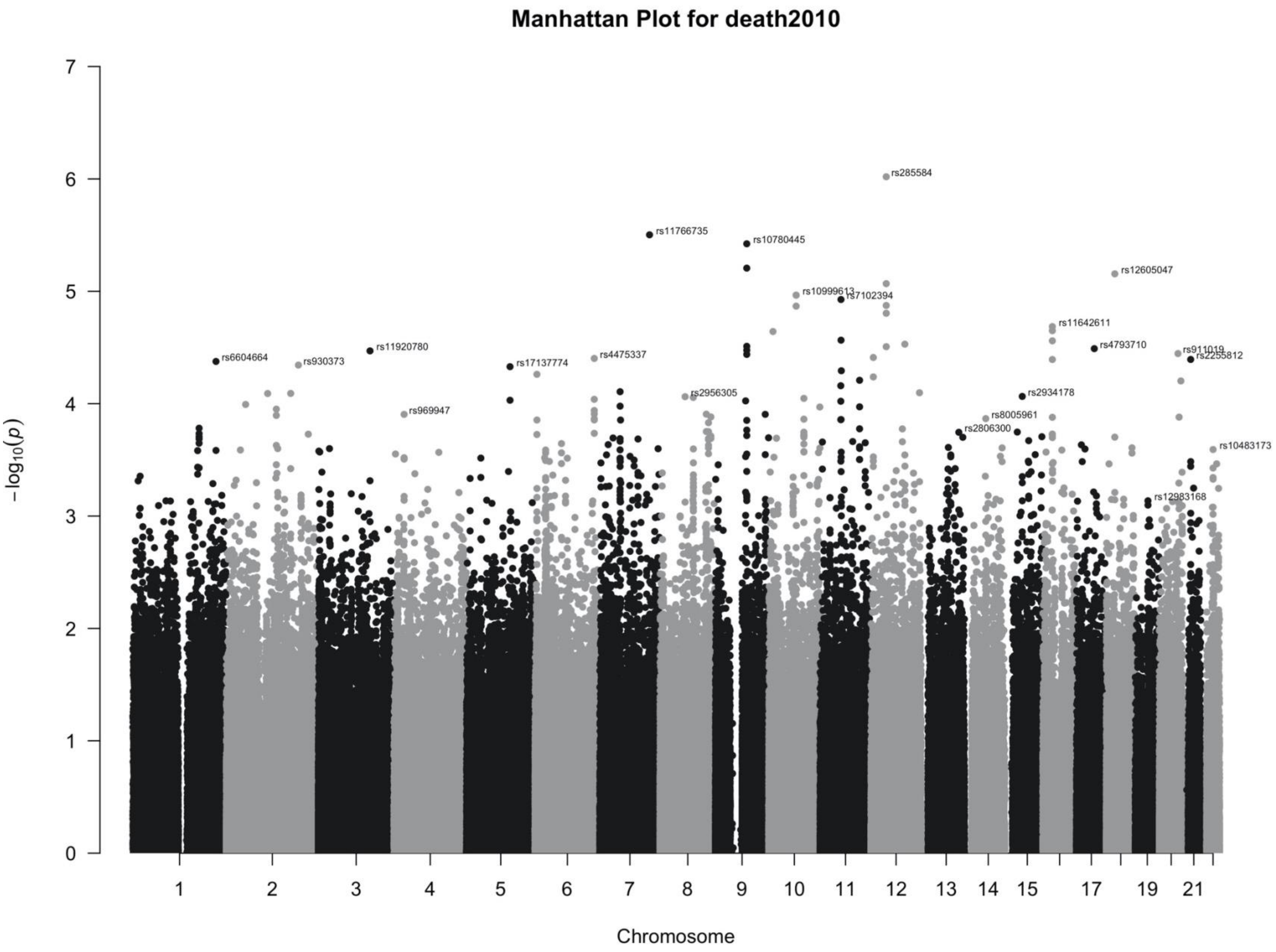
Manhattan Plot for death2010
7
6
5
4
3
2
1
0
−log10(p)
rs285584
rs11766735
rs10780445
rs12605047
rs10999613
rs7102394
rs11642611
rs4793710
rs11920780
rs911019
rs2255812
rs6604664
rs930373
rs17137774
rs4475337
rs2956305
rs2934178
rs969947
rs8005961
rs2806300
rs10483173
rs12983168
1
2
3
4
5
6
7
8
9
10
11
12
13
14
15
17
19
21
Chromosome

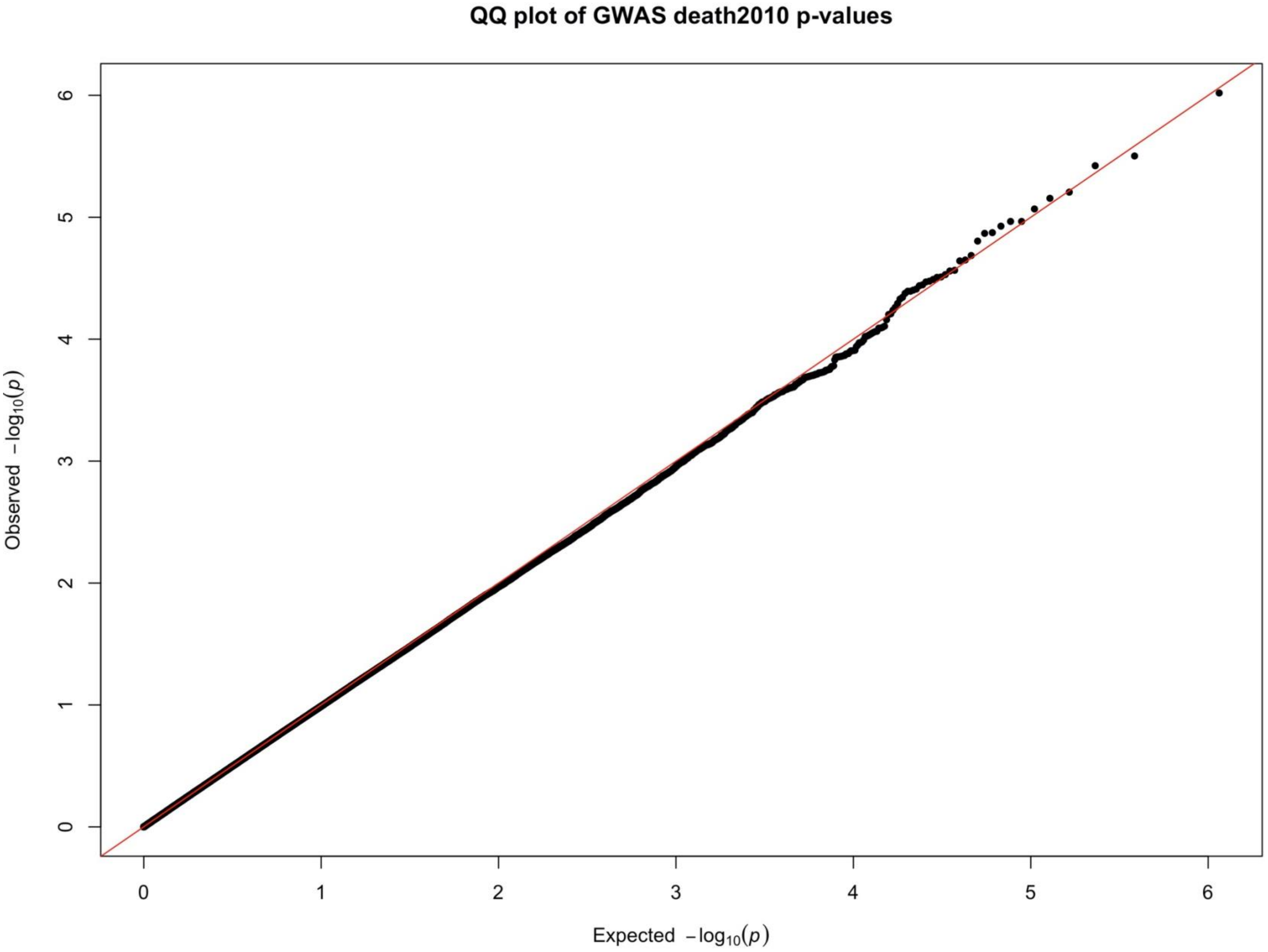
QQ plot of GWAS death2010 p-values
Observed −log10(p)
Expected −log10(p)
0
1
2
3
4
5
6

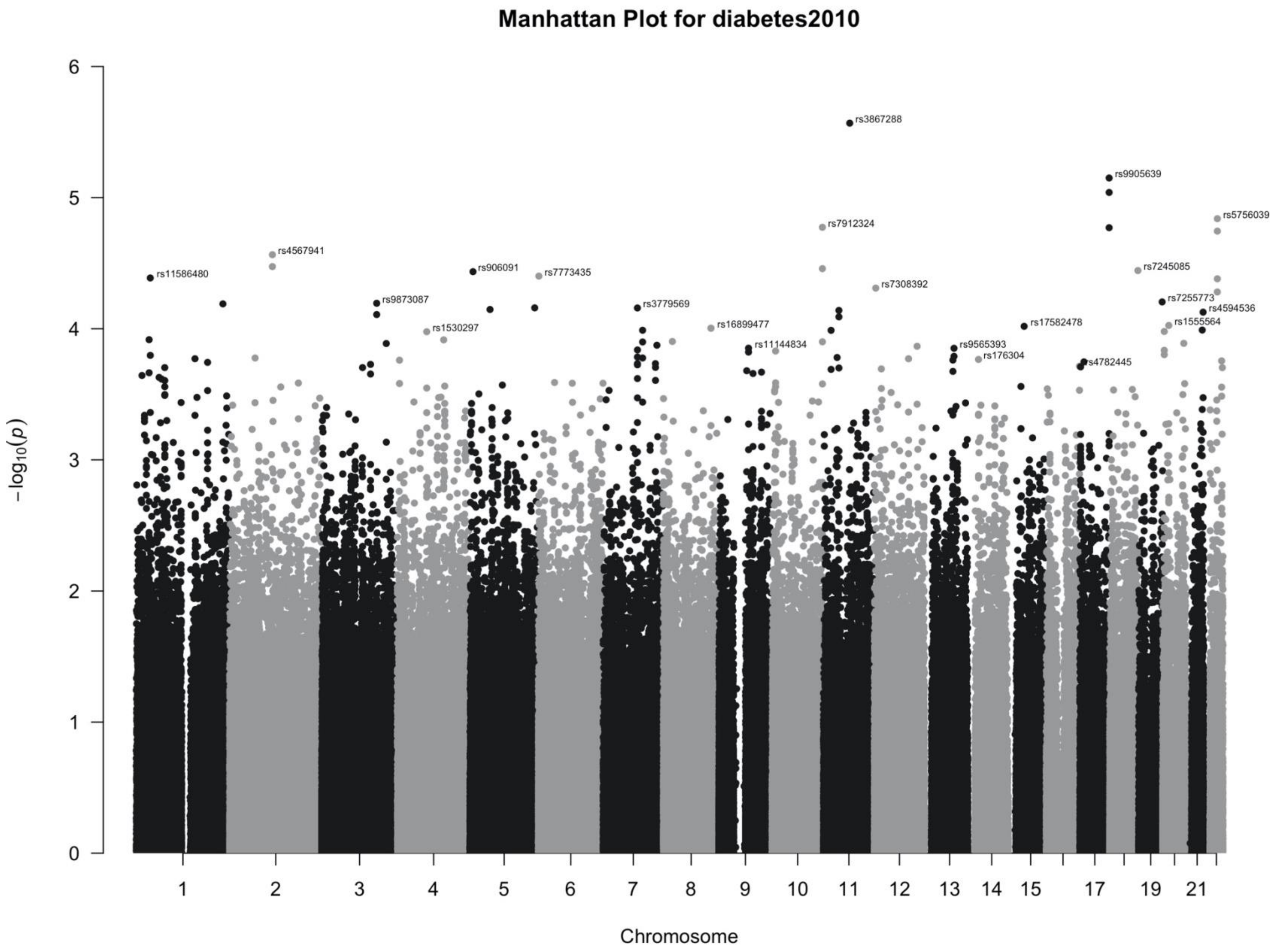
Manhattan Plot for diabetes2010
6
5
4
3
2
1
0
−log10(p)
rs3867288
rs9905639
rs5756039
rs7912324
rs4567941
rs7245085
rs11586480
rs906091
rs7773435
rs7308392
rs9873087
rs7255773
rs3779569
rs4594536
rs1530297
rs16899477
rs17582478
rs1555564
rs11144834
rs9565393
rs176304
rs4782445
1
2
3
4
5
6
7
8
9
10
11
12
13
14
15
17
19
21
Chromosome

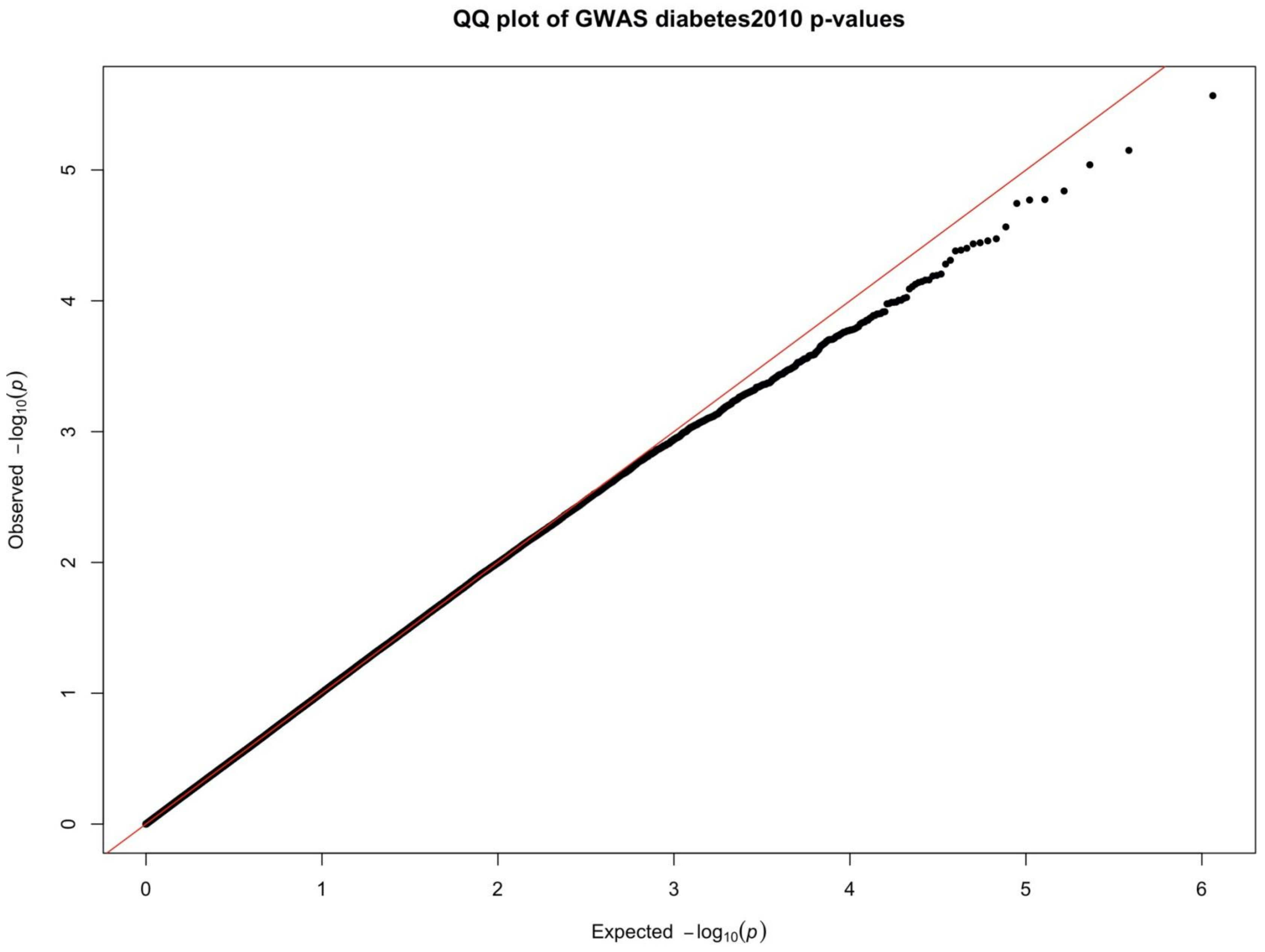

QQ plot of GWAS diabetes2010 p-values
Observed −log10(p)
Expected −log10(p)
0
1
2
3
4
5
6

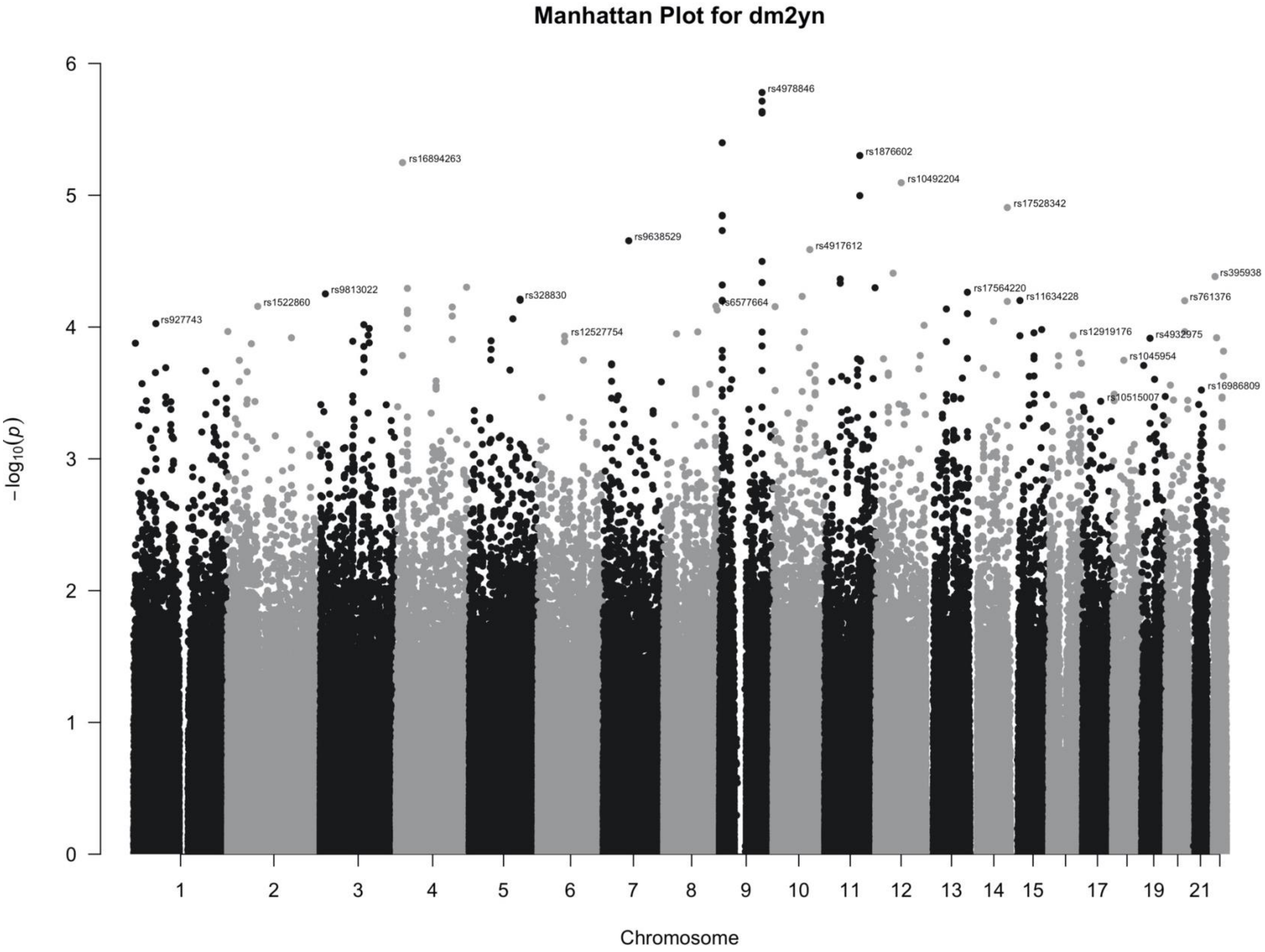
Manhattan Plot for dm2yn
−log10(p)
Chromosome
0
1
2
3
4
5
6
1
2
3
4
5
6
7
8
9
10
11
12
13
14
15
17
19
21
rs4978846
rs16894263
rs1876602
rs10492204
rs17528342
rs9638529
rs4917612
rs395938
rs9813022
rs328830
rs1522860
rs927743
rs12527754
s6577664
rs17564220
rs11634228
rs761376
rs12919176
rs4932975
rs1045954
rs16986809
rs10515007

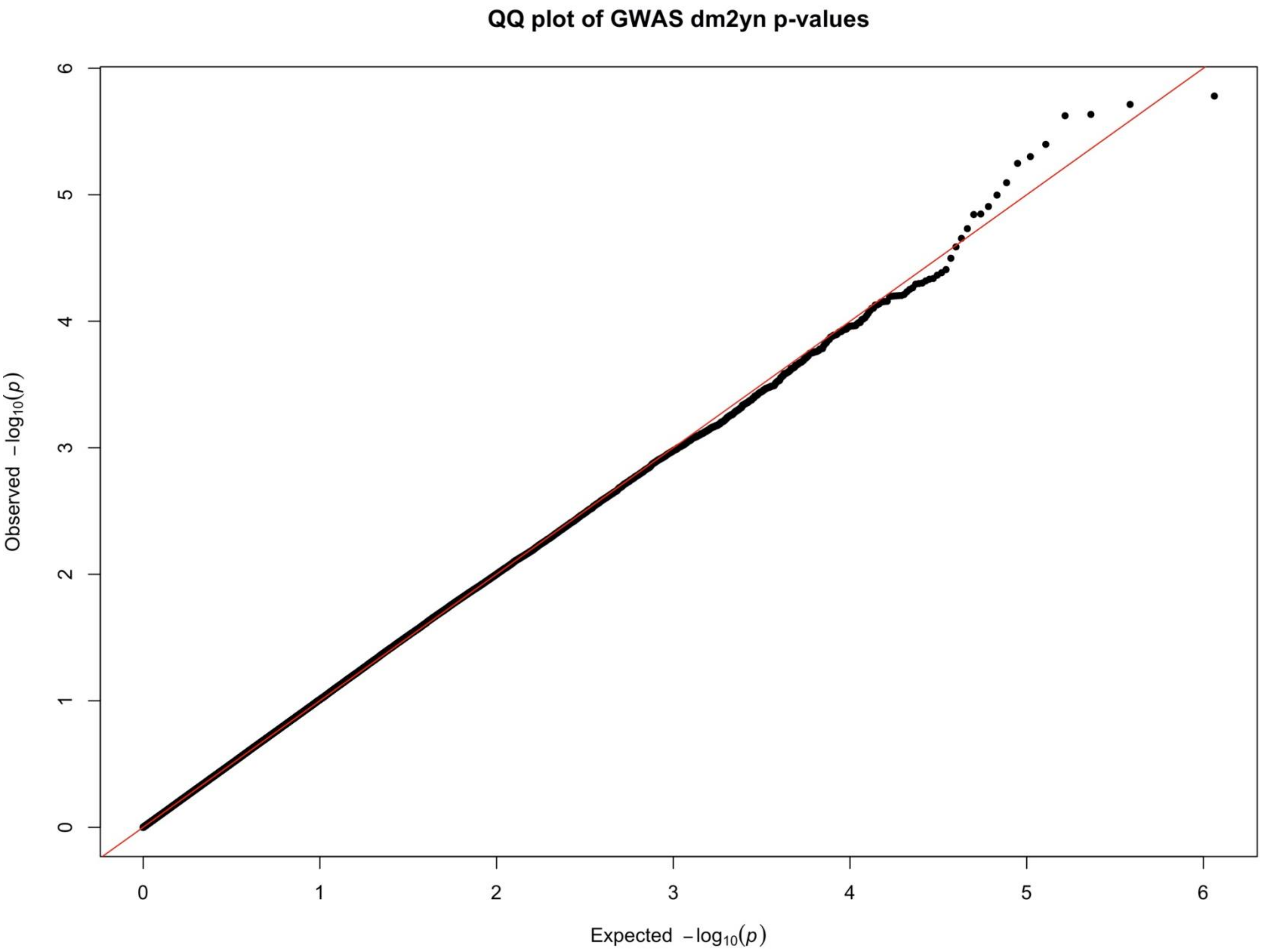
QQ plot of GWAS dm2yn p-values
Observed −log10(p)
Expected −log10(p)
0
1
2
3
4
5
6

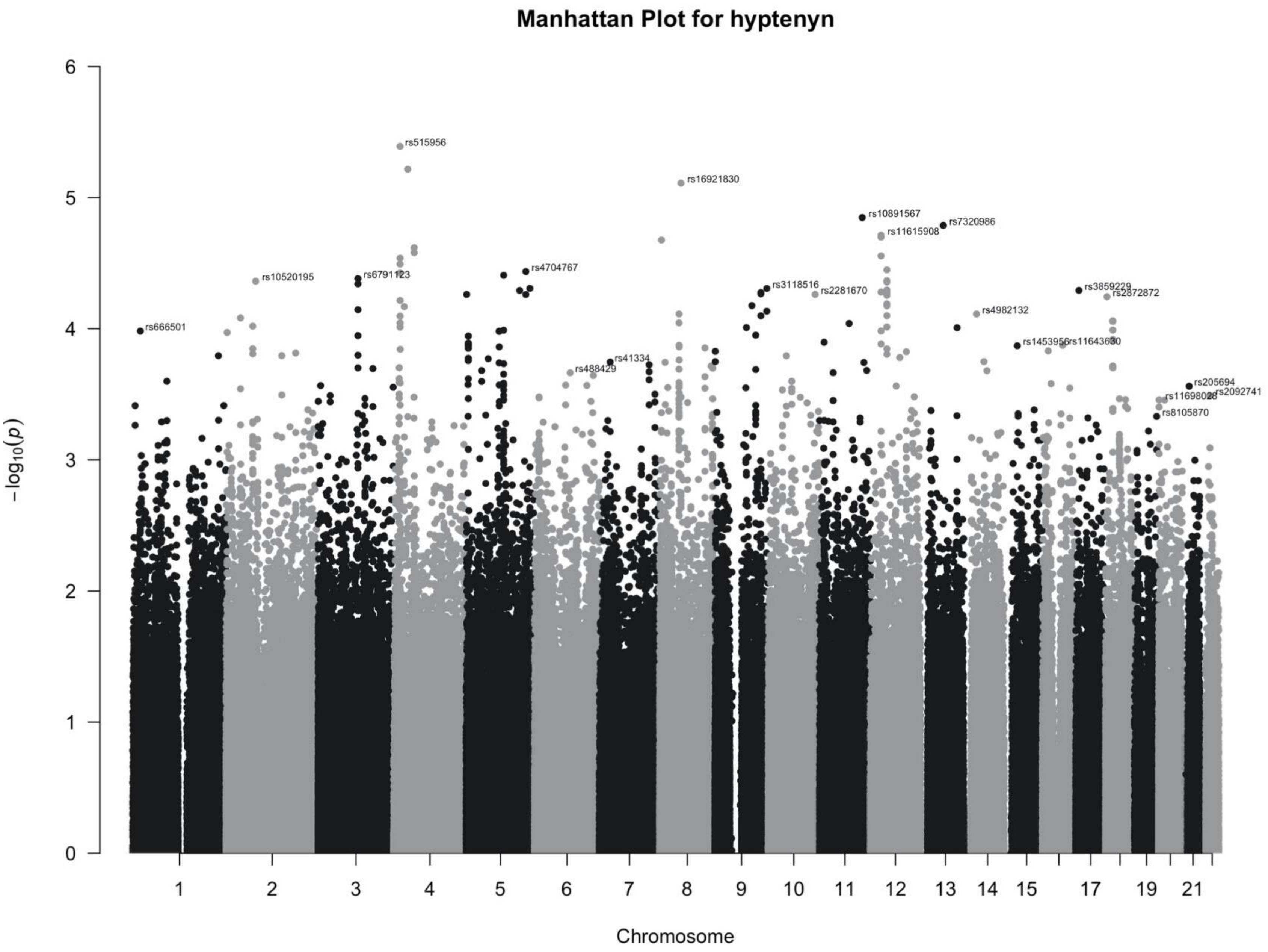
Manhattan Plot for hyptenyn
−log10(p)
0
1
2
3
4
5
6
Chromosome
1
2
3
4
5
6
7
8
9
10
11
12
13
14
15
17
19
21
rs515956
rs16921830
rs10891567
rs7320986
rs11615908
rs4704767
rs10520195
rs6791123
rs3118516
rs2281670
rs3859229
rs2872872
rs4982132
rs666501
rs1453956rs11643630
rs41334
rs488429
rs205694
rs11698028rs2092741
rs8105870

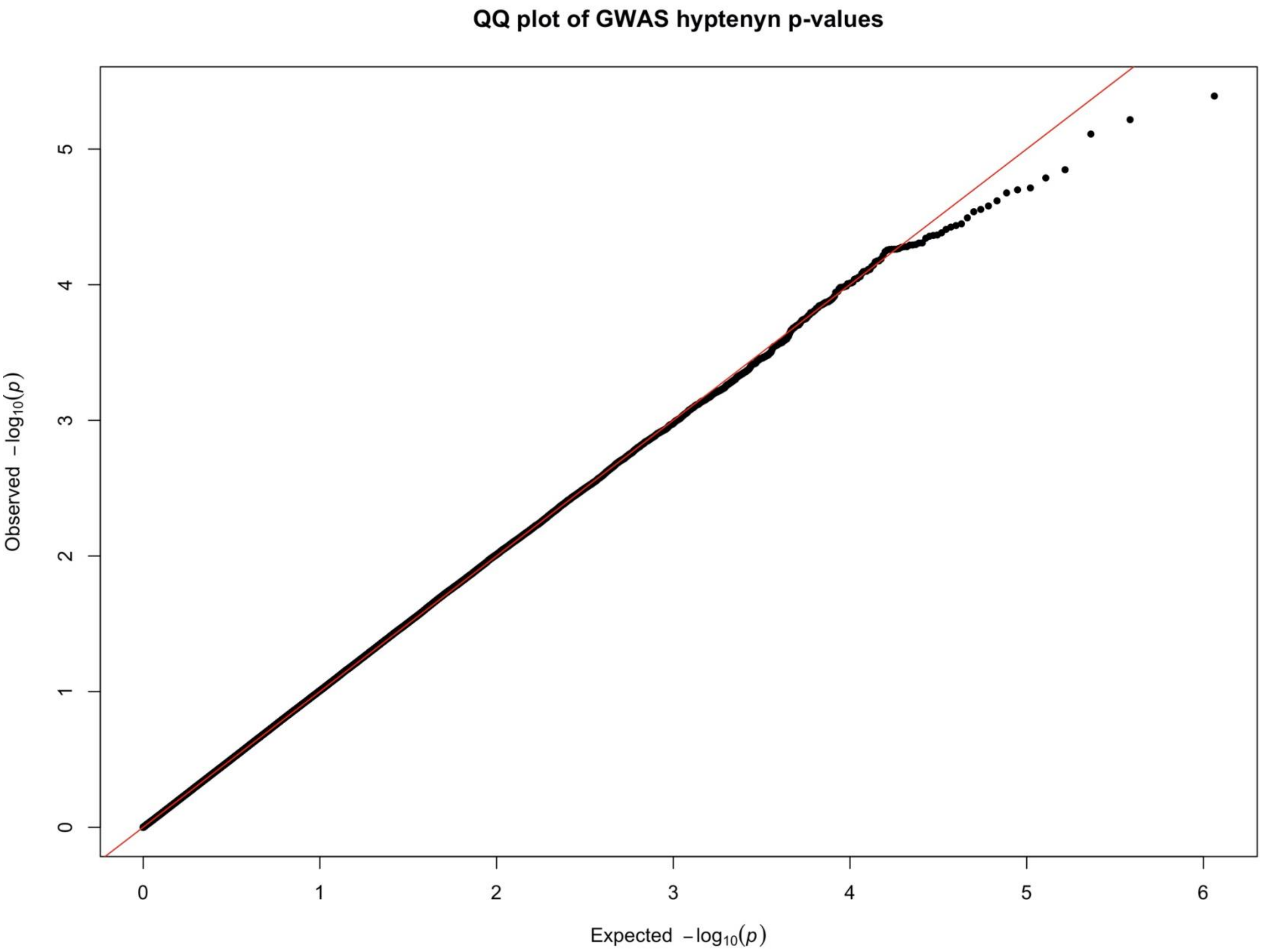
QQ plot of GWAS hyptenyn p-values
Observed −log10(p)
Expected −log10(p)
0
1
2
3
4
5
6

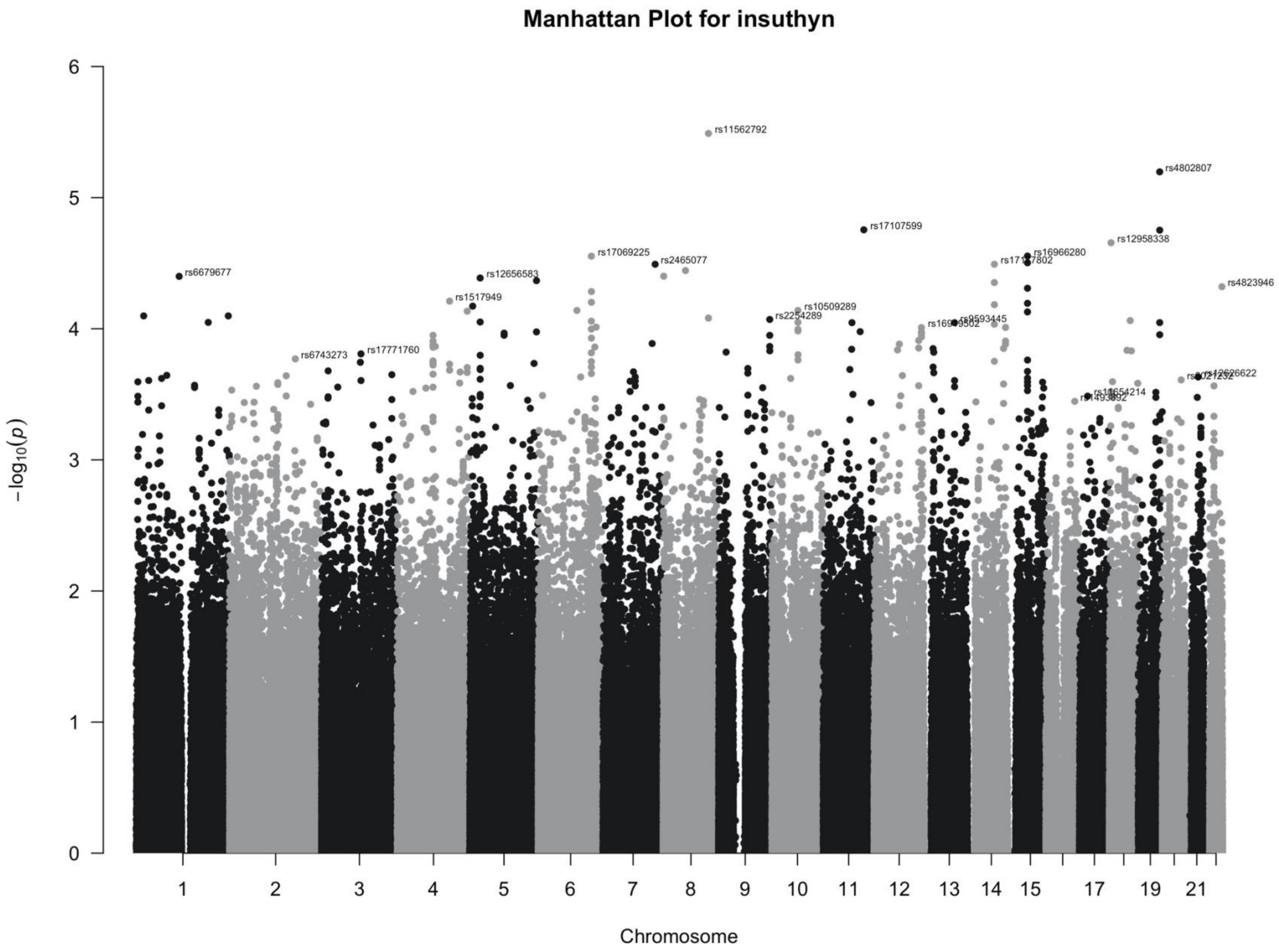
Manhattan Plot for insuthyn
6
5
4
3
2
1
0
−log10(p)
rs11562792
rs4802807
rs17107599
rs12958338
rs16966280
rs17069225
rs2465077
rs6679677
rs12656583
rs4823946
rs1517949
rs10509289
rs2254289
rs9593445
rs6743273
rs17771760
rs11654214
rs12626622
1
2
3
4
5
6
7
8
9
10
11
12
13
14
15
17
19
21
Chromosome

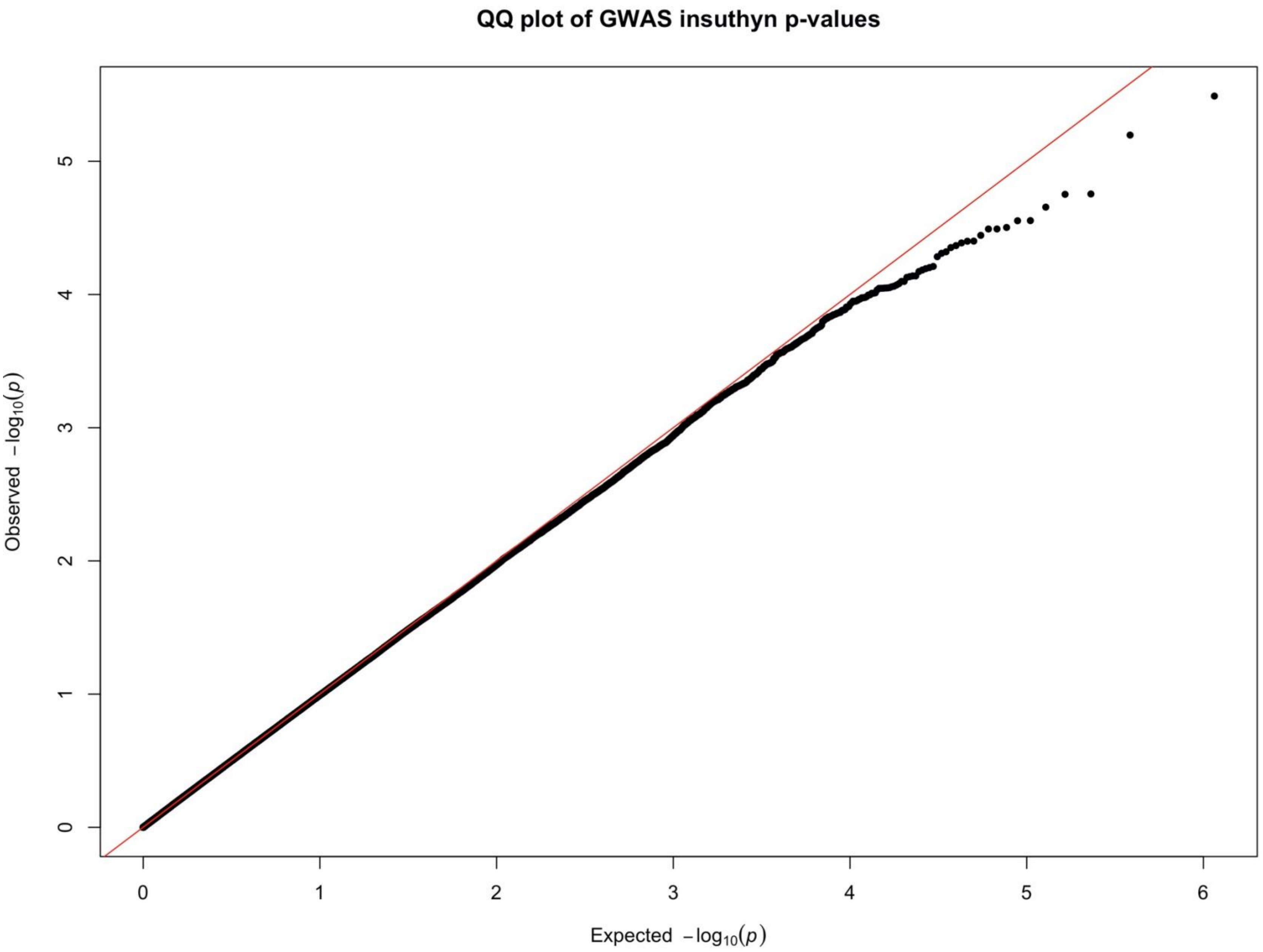
QQ plot of GWAS insuthyn p-values
Observed −log10(p)
Expected −log10(p)
0
1
2
3
4
5
6

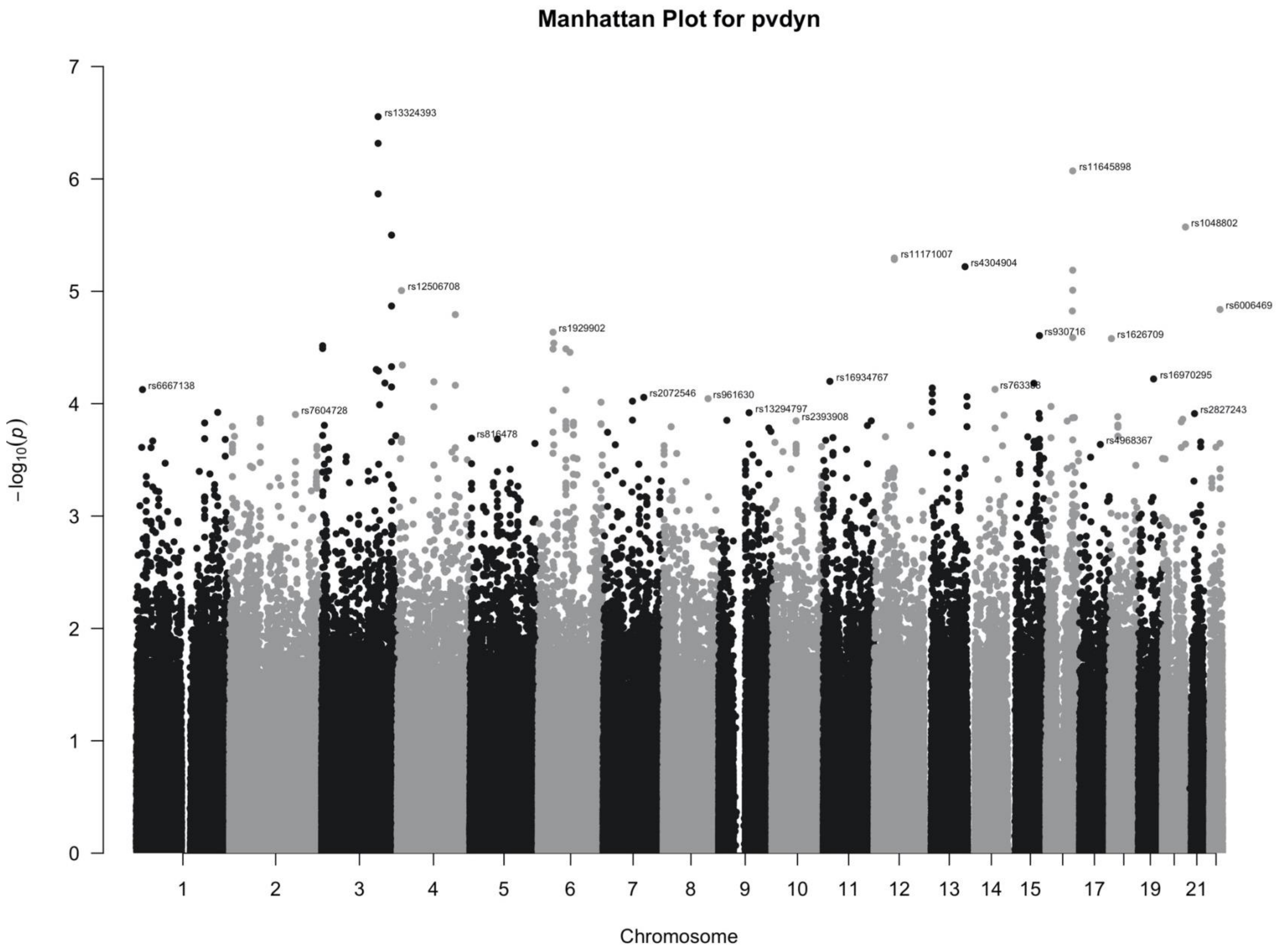
Manhattan Plot for pvdyn
−log10(p)
Chromosome
0
1
2
3
4
5
6
7
1 2 3 4 5 6 7 8 9 10 11 12 13 14 15 17 19 21
rs13324393
rs12506708
rs1929902
rs6667138
rs7604728
rs816478
rs2072546
rs961630
rs13294797
rs2393908
rs16934767
rs11171007
rs4304904
rs11645898
rs1048802
rs6006469
rs930716
rs1626709
rs16970295
rs2827243
rs4968367

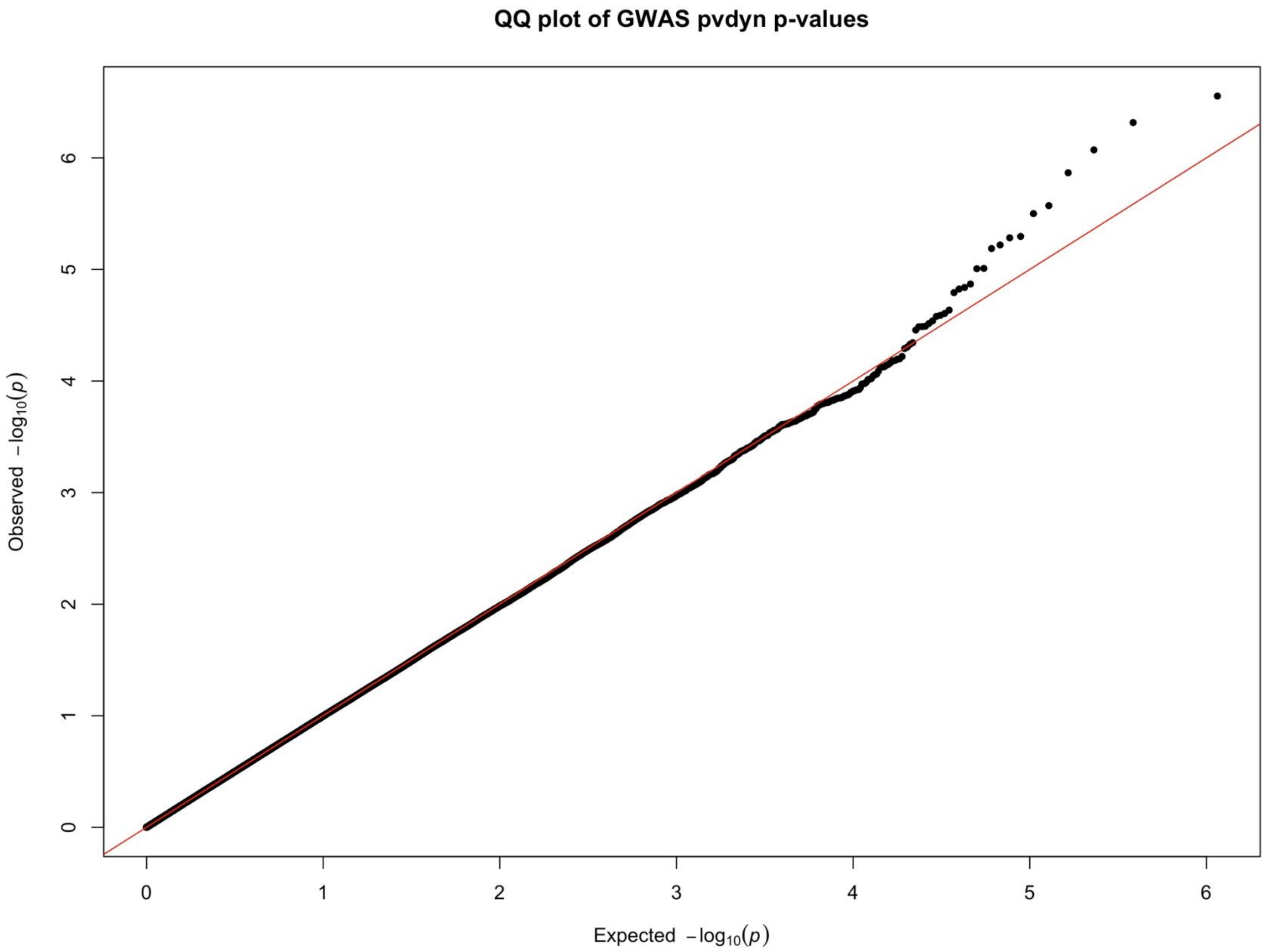
QQ plot of GWAS pvdyn p-values
Observed $-\log_{10}(p)$
Expected $-\log_{10}(p)$
0
1
2
3
4
5
6

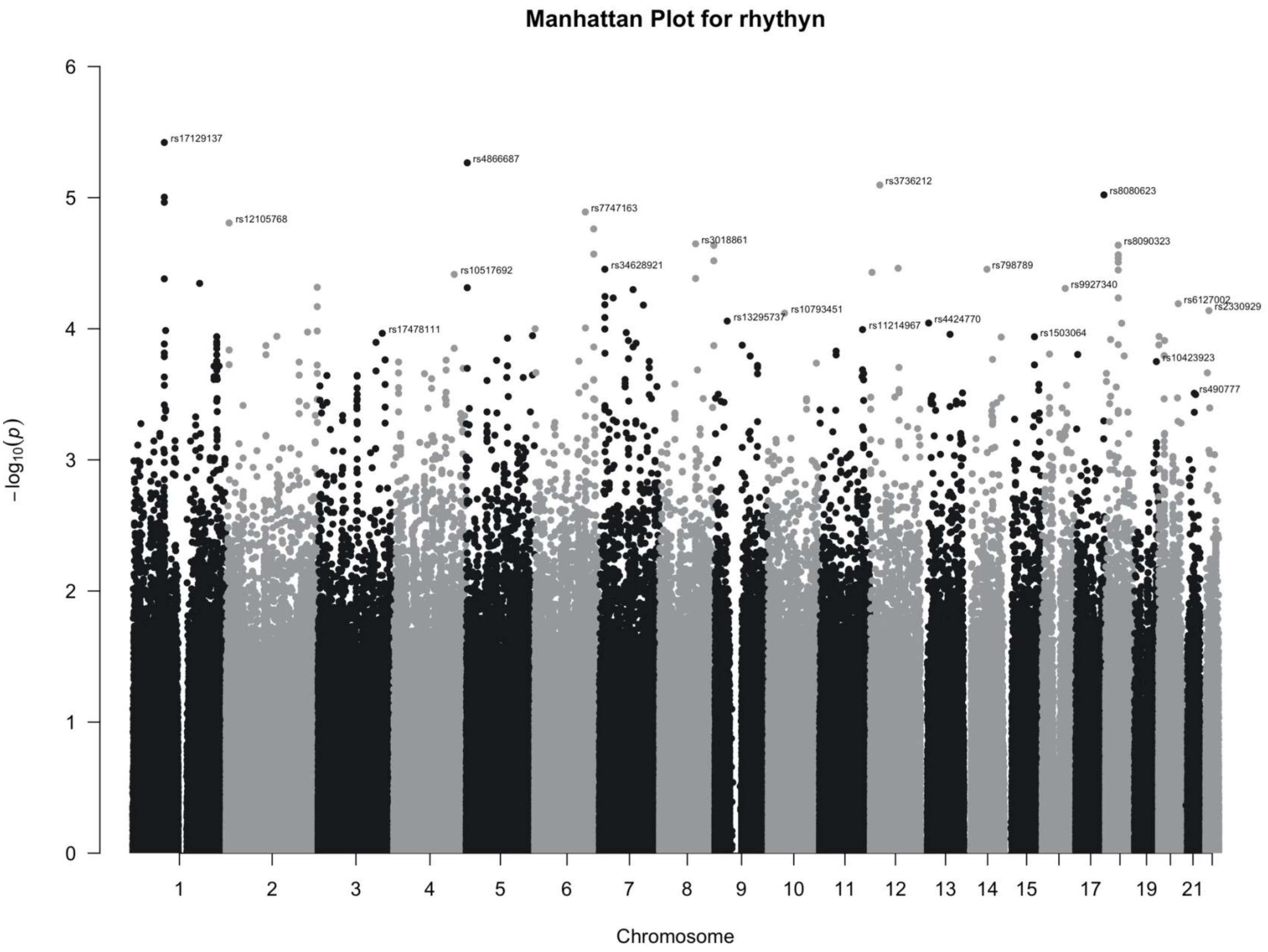
Manhattan Plot for rhythyn
rs17129137
rs4866687
rs3736212
rs8080623
rs7747163
rs12105768
rs3018861
rs8090323
rs34628921
rs798789
rs10517692
rs9927340
rs6127002
rs2330929
rs10793451
rs13295737
rs11214967
rs4424770
rs17478111
rs1503064
rs10423923
rs490777
−log10(p)
Chromosome
1 2 3 4 5 6 7 8 9 10 11 12 13 14 15 17 19 21

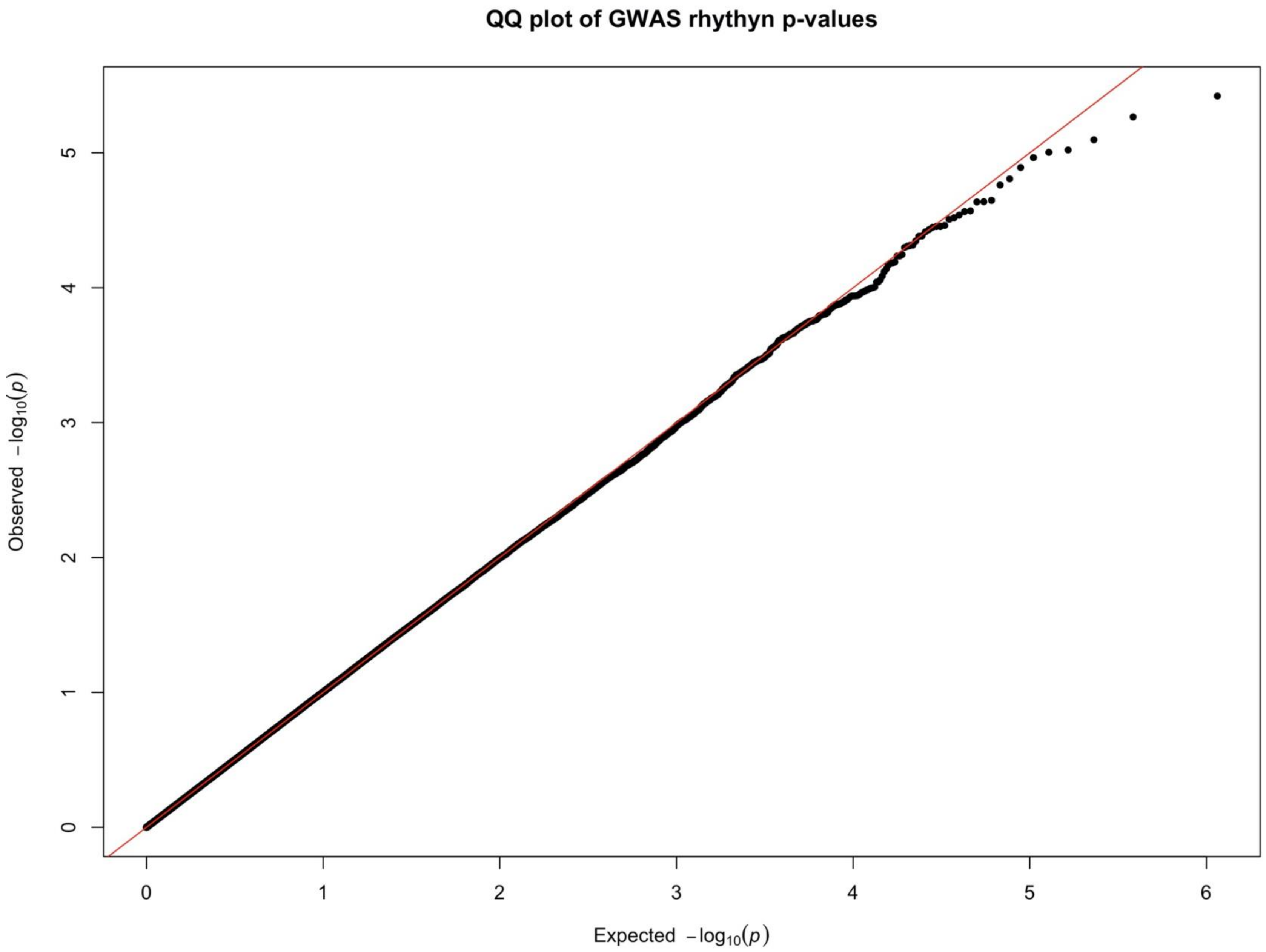
QQ plot of GWAS rhythyn p-values
Observed $-\log_{10}(p)$
Expected $-\log_{10}(p)$
0
1
2
3
4
5
6

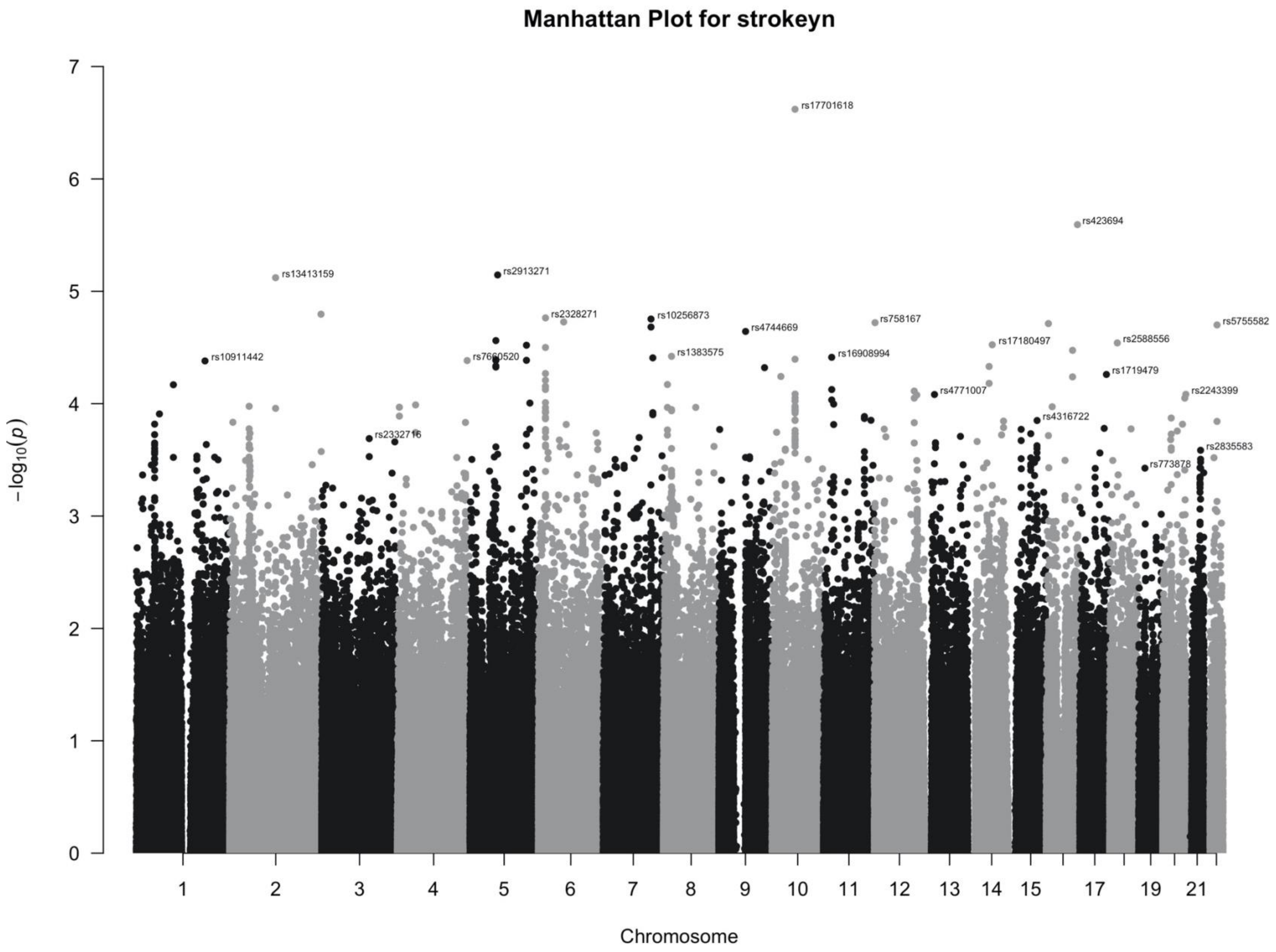
Manhattan Plot for strokeyn
rs17701618
rs423694
rs13413159
rs2913271
rs2328271
rs10256873
rs758167
rs5755582
rs4744669
rs17180497
rs2588556
rs1383575
rs16908994
rs10911442
rs7660520
rs1719479
rs4771007
rs2243399
rs4316722
rs2332716
rs2835583
rs773878
−log10(p)
Chromosome
0
1
2
3
4
5
6
7
1 2 3 4 5 6 7 8 9 10 11 12 13 14 15 17 19 21

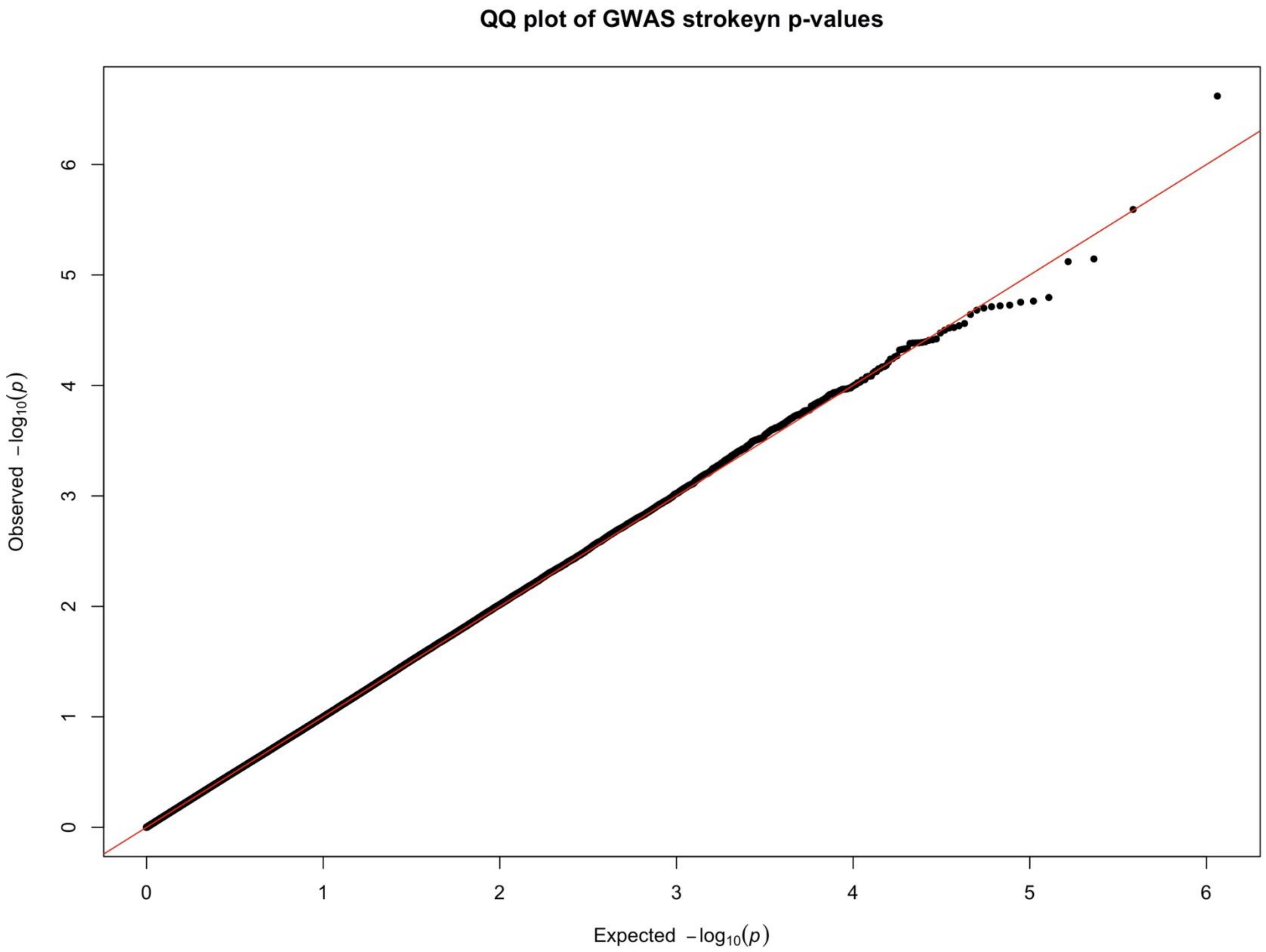
QQ plot of GWAS strokeyn p-values
Observed $-\log_{10}(p)$
Expected $-\log_{10}(p)$
0
1
2
3
4
5
6
0
1
2
3
4
5
6

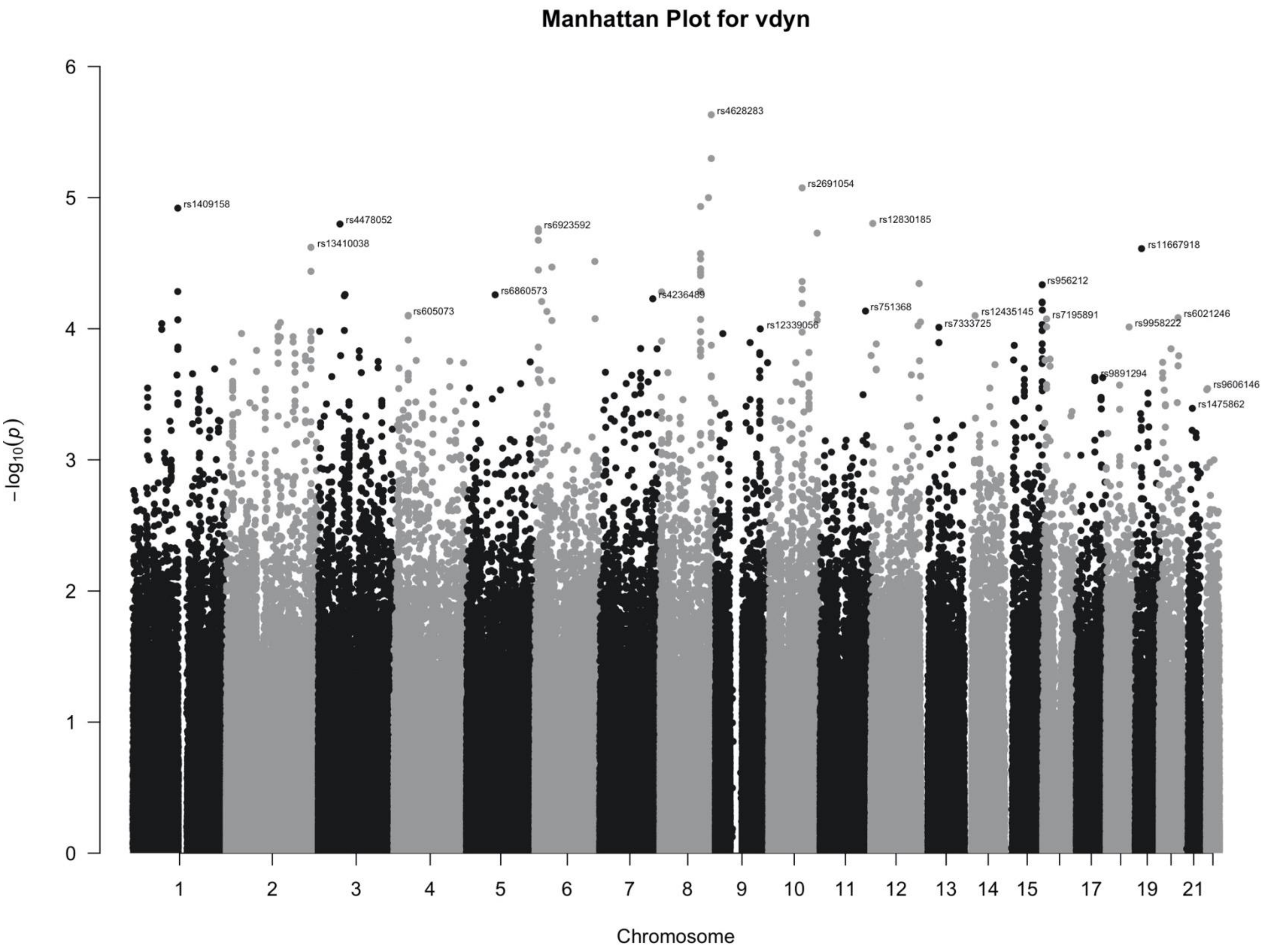
Manhattan Plot for vdyn
rs4628283
rs2691054
rs1409158
rs4478052
rs6923592
rs12830185
rs13410038
rs11667918
rs956212
rs6860573
rs4236489
rs751368
rs605073
rs12435145
rs7195891
rs6021246
rs12339056
rs7333725
rs9958222
rs9891294
rs9606146
rs1475862
−log10(p)
Chromosome
0
1
2
3
4
5
6
1
2
3
4
5
6
7
8
9
10
11
12
13
14
15
17
19
21

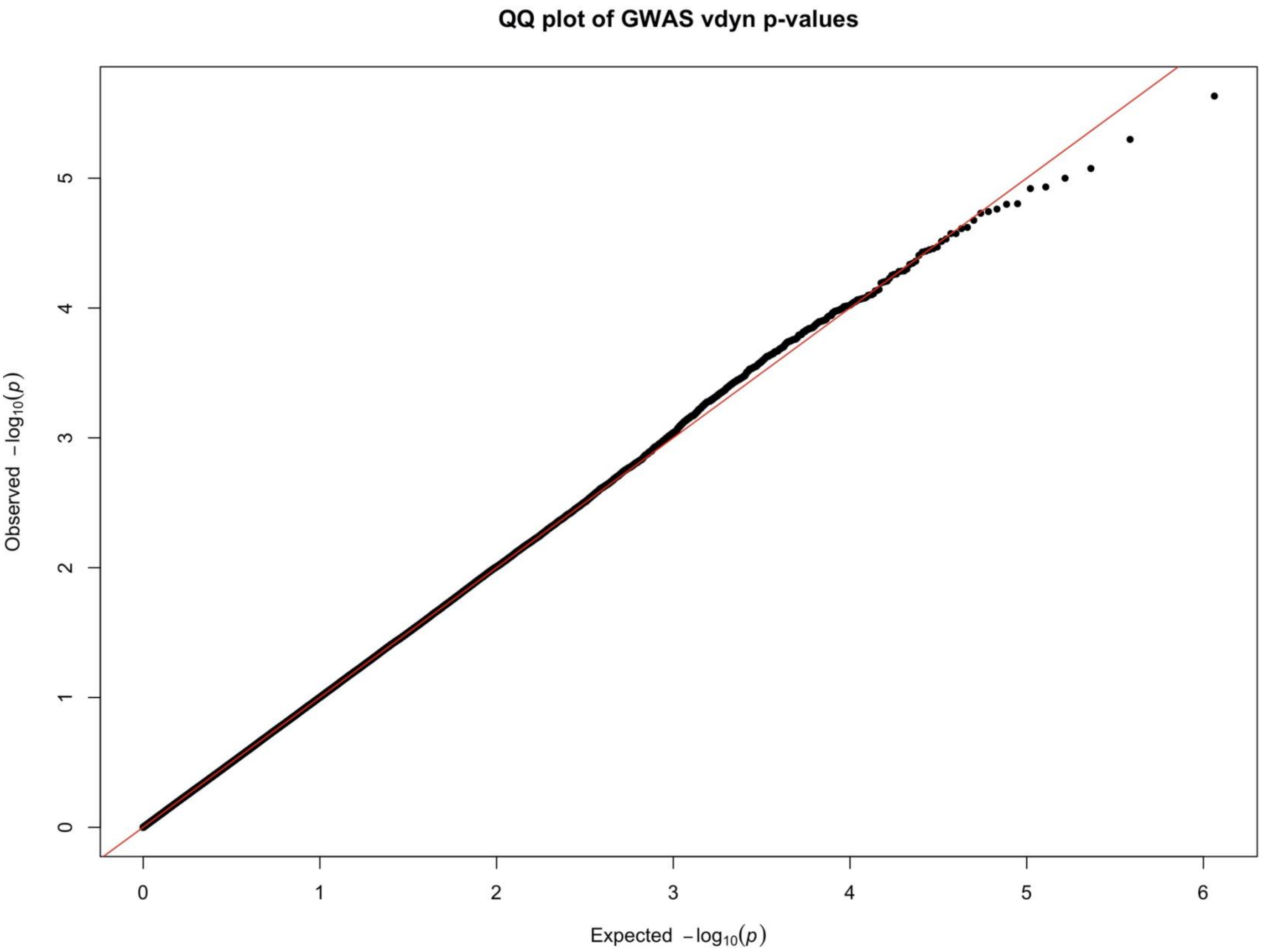
QQ plot of GWAS vdyn p-values
Observed −log10(p)
Expected −log10(p)
0
1
2
3
4
5
6

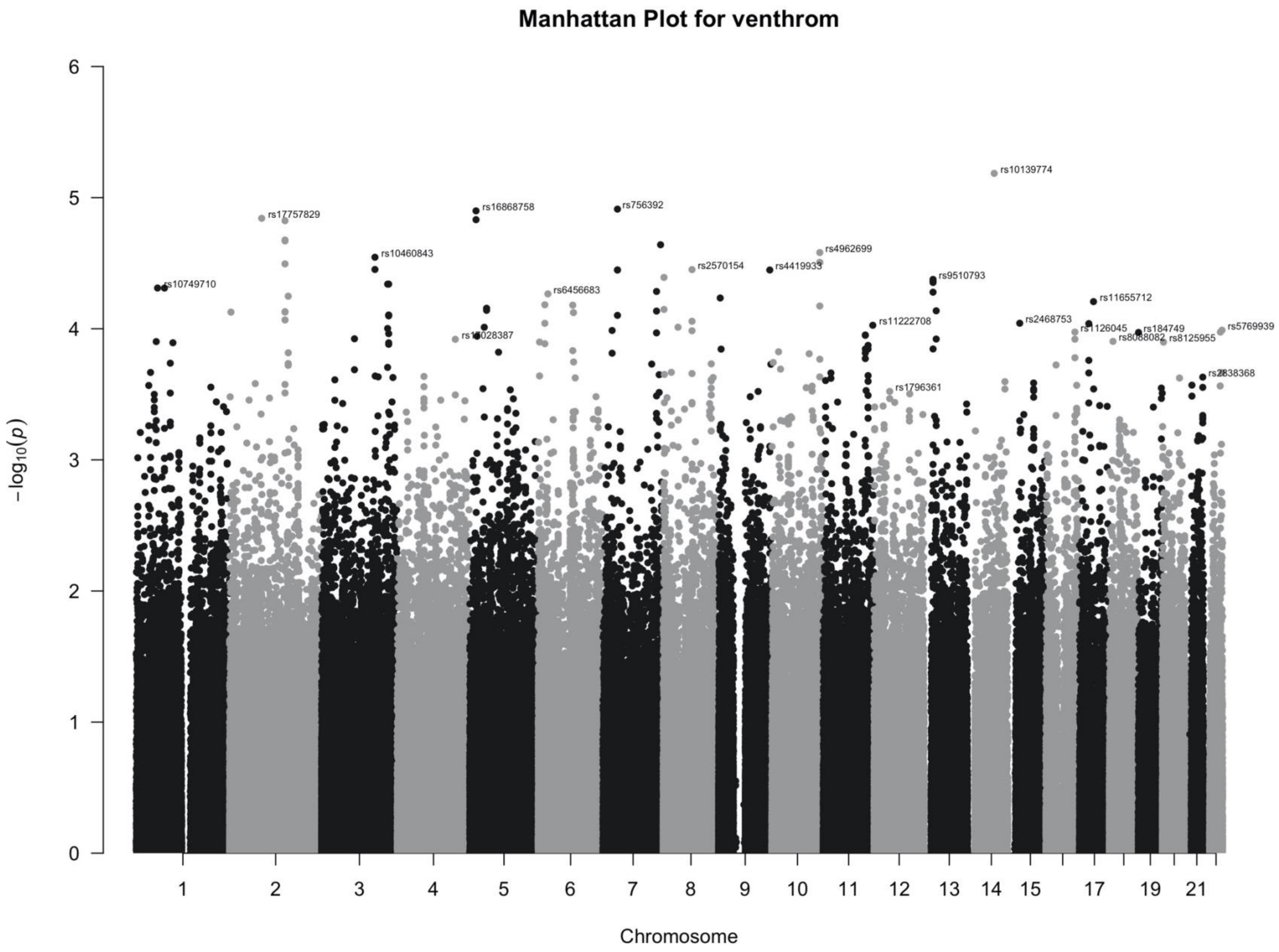
Manhattan Plot for venthrom
−log10(p)
Chromosome
rs10139774
rs17757829
rs16868758
rs756392
rs10460843
rs4962699
rs2570154
rs4419933
rs9510793
rs10749710
rs6456683
rs11655712
rs11222708
rs2468753
rs1126045
rs184749
rs5769939
rs8088082
rs8125955
rs1796361

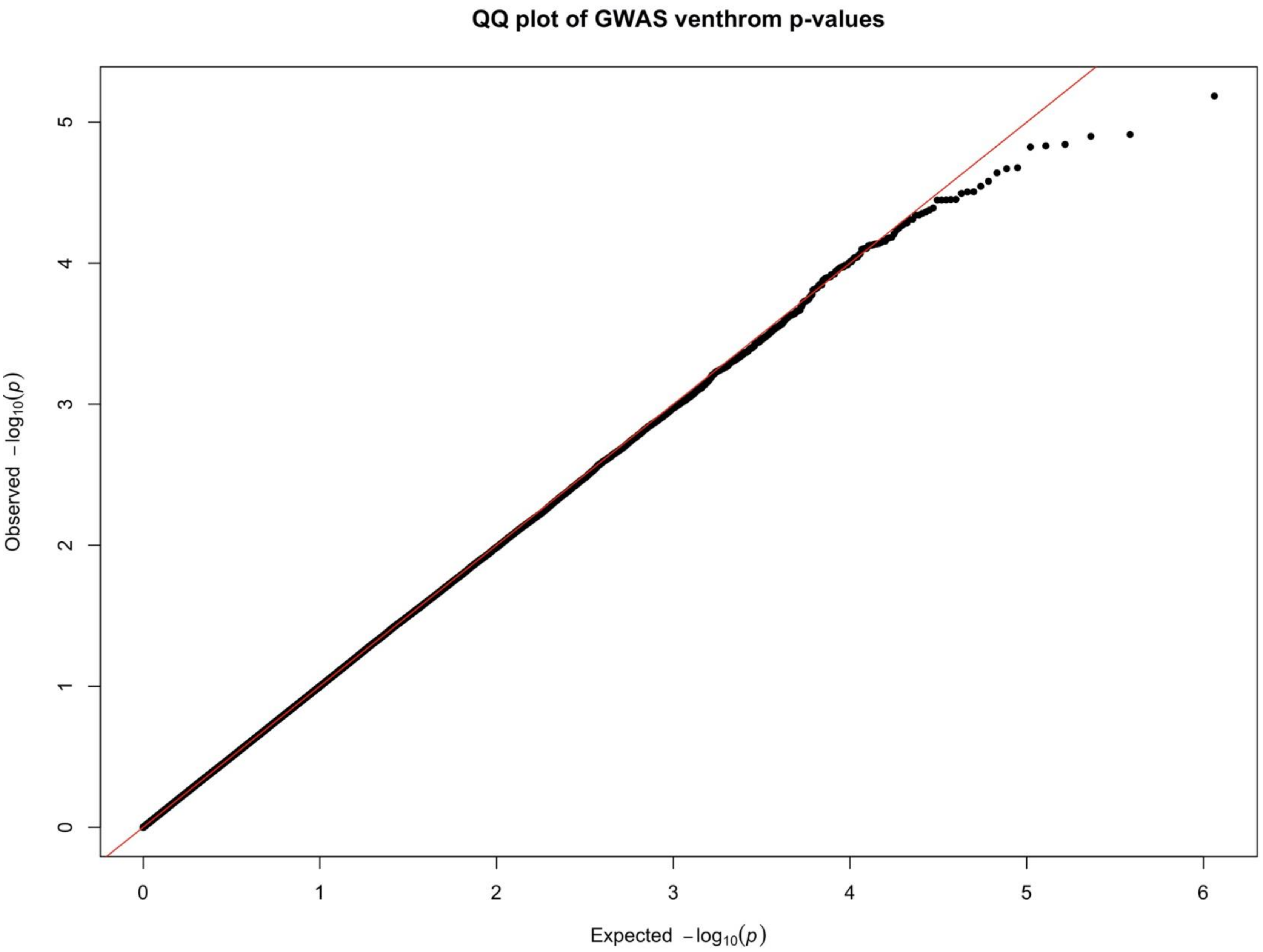
QQ plot of GWAS venthrom p-values
Observed −log10(p)
Expected −log10(p)
0
1
2
3
4
5
6

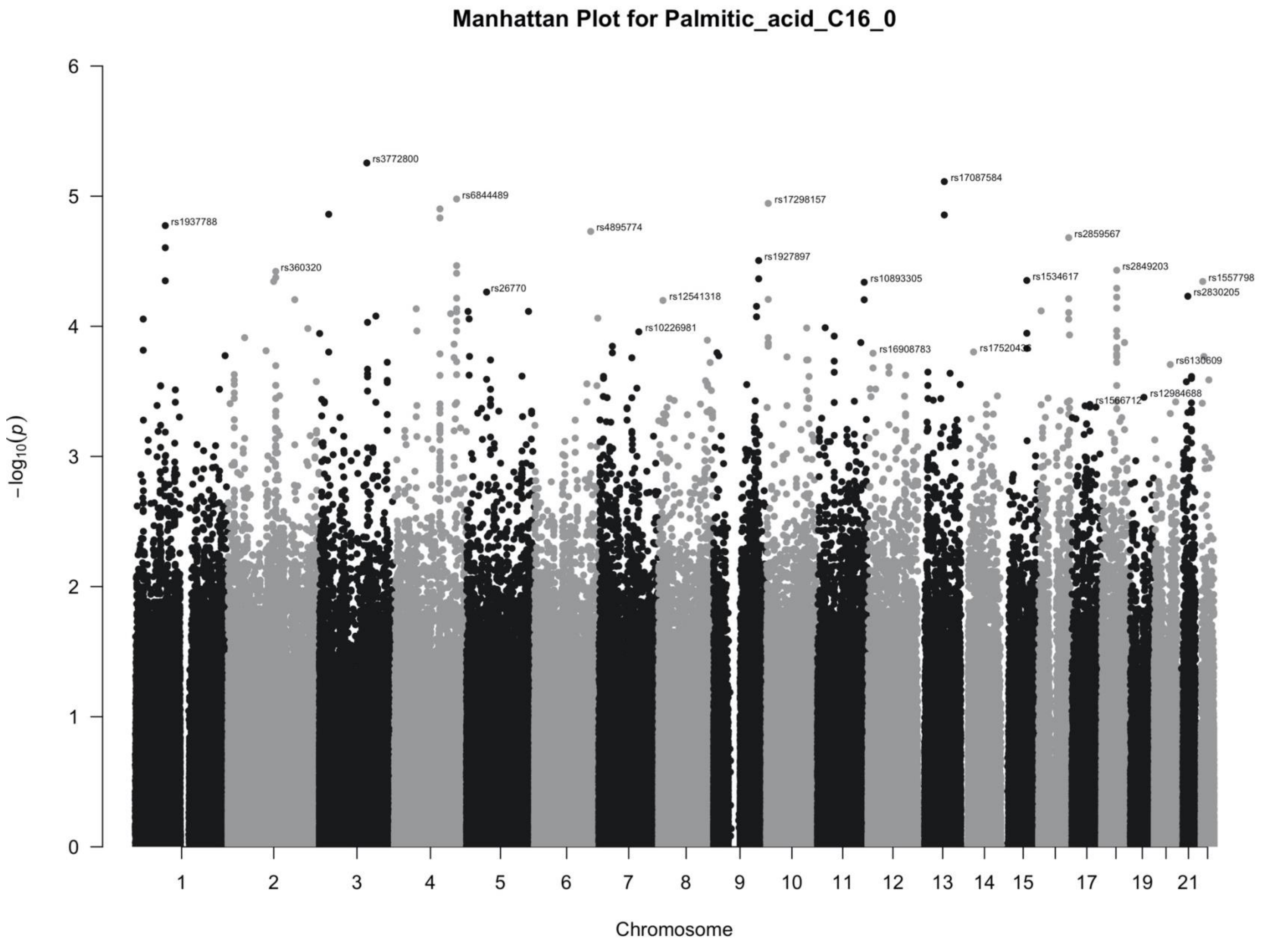

Manhattan Plot for Palmitic_acid_C16_0
−log10(p)
Chromosome
rs3772800
rs17087584
rs6844489
rs17298157
rs1937788
rs4895774
rs2859567
rs1927897
rs360320
rs2849203
rs26770
rs10893305
rs1534617
rs1557798
rs2830205
rs12541318
rs10226981
rs16908783
rs6130609
rs12984688
0
1
2
3
4
5
6
1
2
3
4
5
6
7
8
9
10
11
12
13
14
15
17
19
21

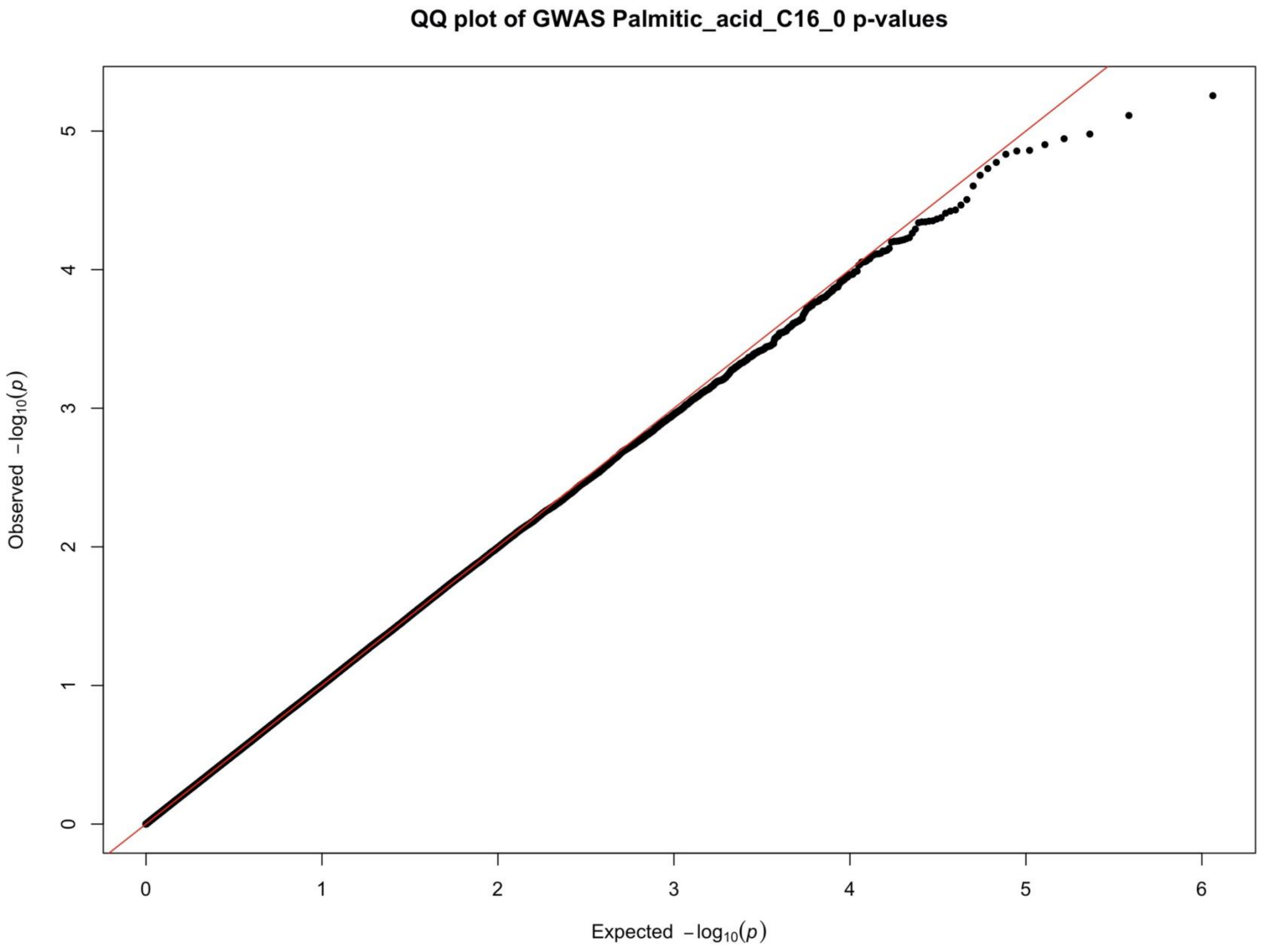
QQ plot of GWAS Palmitic_acid_C16_0 p-values
Observed −log10(p)
Expected −log10(p)
0
1
2
3
4
5
6

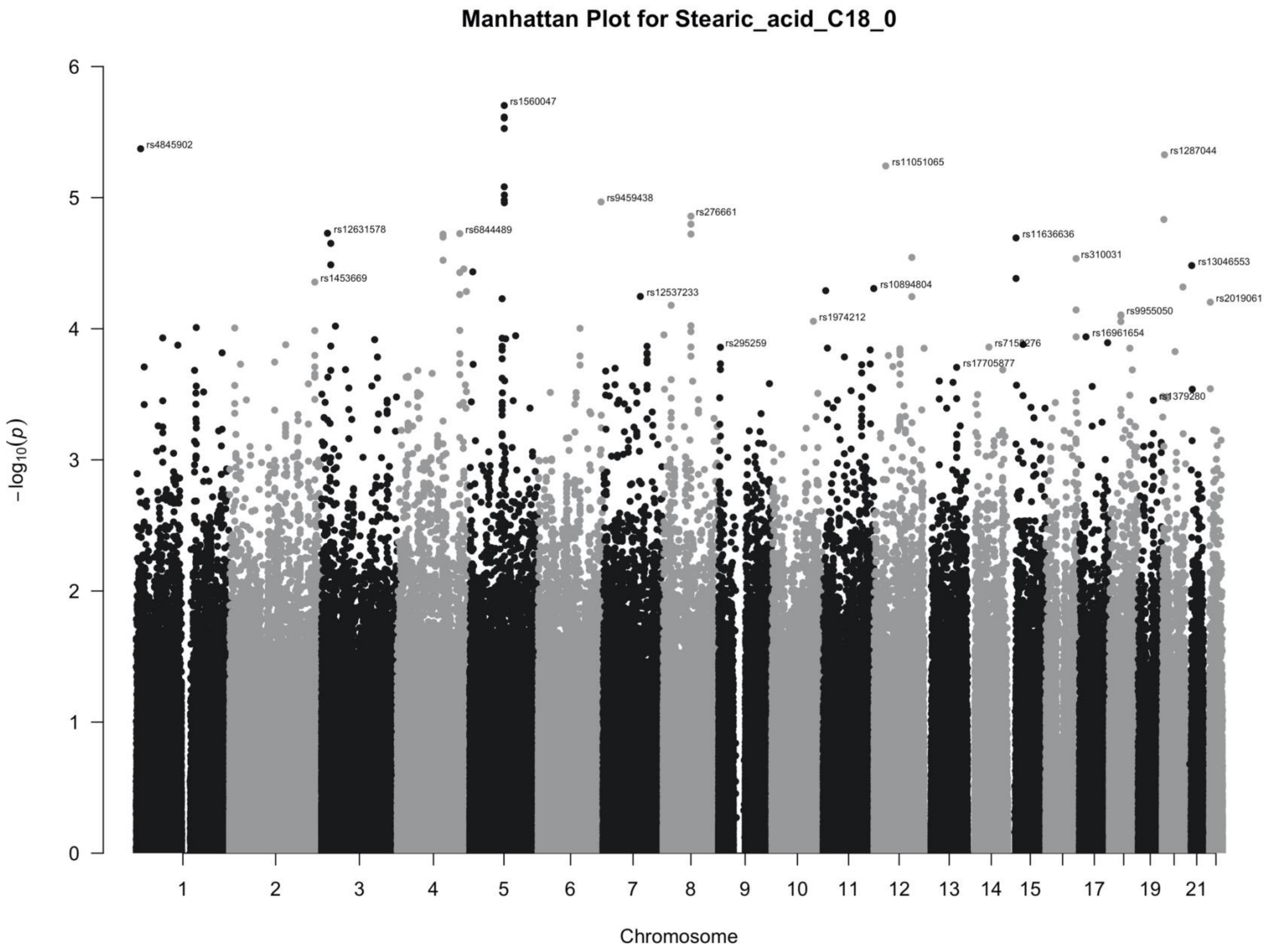
Manhattan Plot for Stearic_acid_C18_0
rs1560047
rs4845902
rs1287044
rs11051065
rs9459438
rs276661
rs12631578
rs6844489
rs11636636
rs310031
rs13046553
rs1453669
rs10894804
rs12537233
rs2019061
rs9955050
rs1974212
rs16961654
rs295259
rs17705877
rs1379280
−log10(p)
Chromosome
0
1
2
3
4
5
6
1
2
3
4
5
6
7
8
9
10
11
12
13
14
15
17
19
21

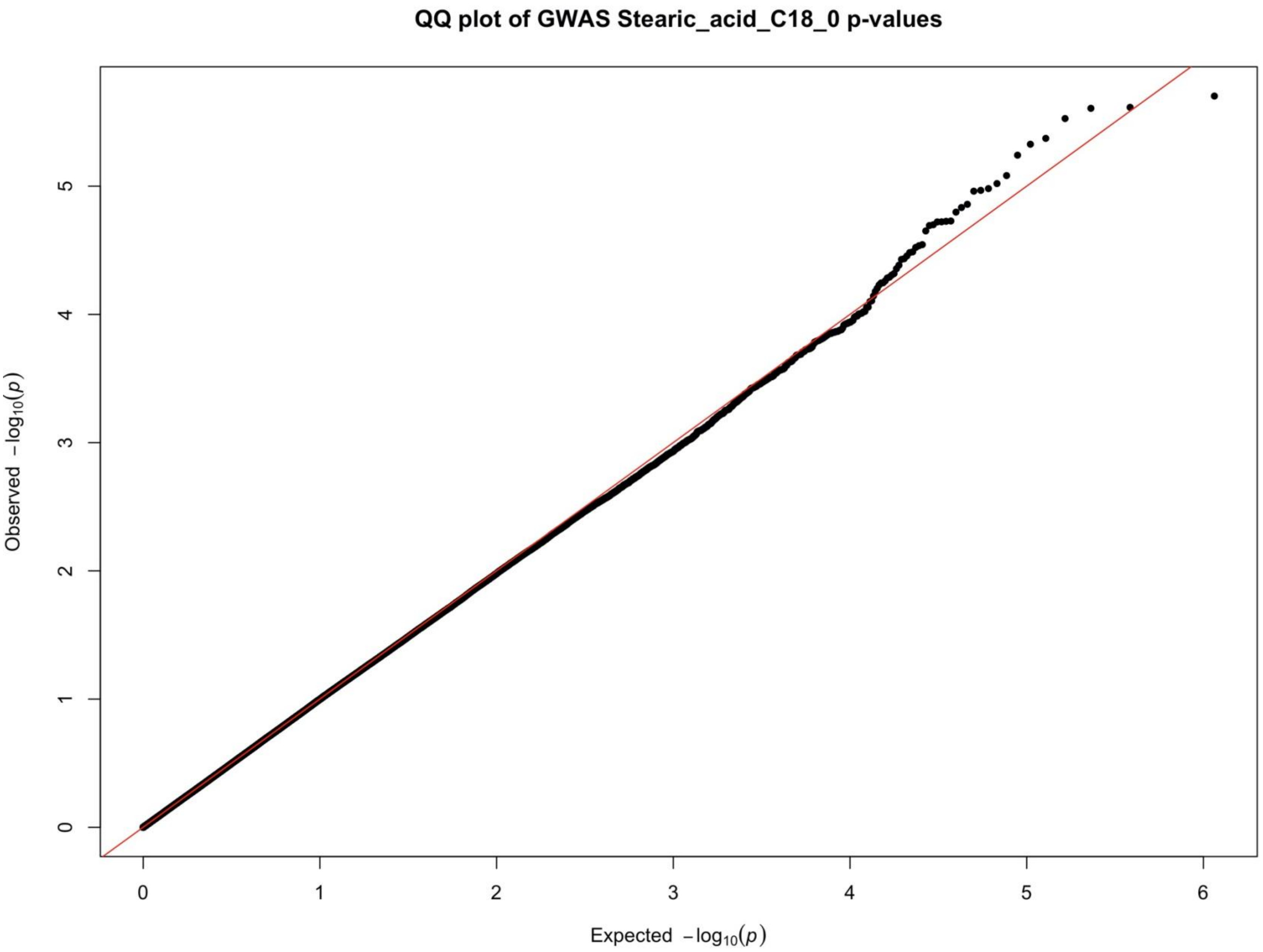
QQ plot of GWAS Stearic_acid_C18_0 p-values
Observed −log10(p)
Expected −log10(p)
0
1
2
3
4
5
6

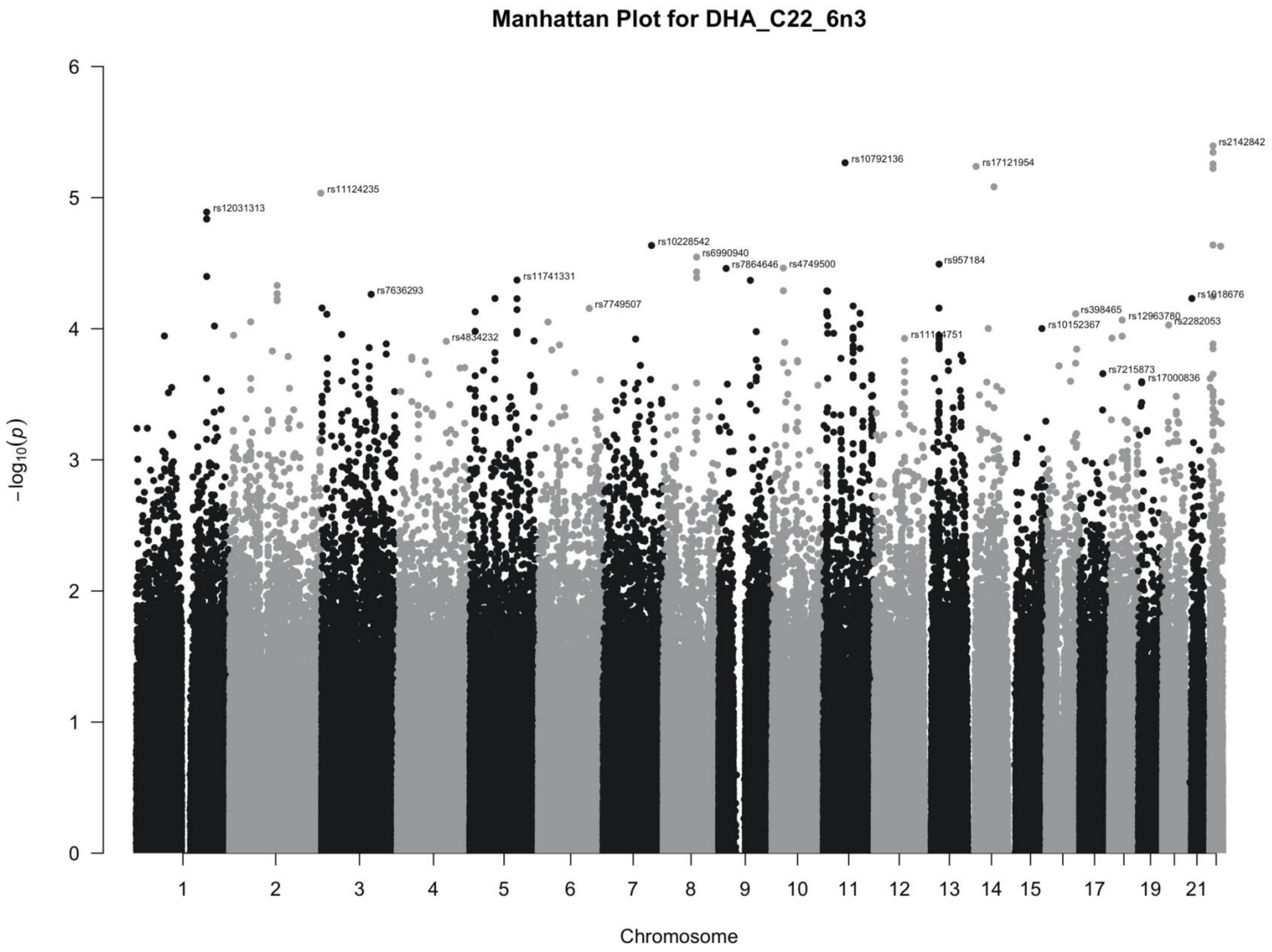
Manhattan Plot for DHA_C22_6n3
−log10(p)
6
5
4
3
2
1
0
Chromosome
1
2
3
4
5
6
7
8
9
10
11
12
13
14
15
17
19
21
rs12031313
rs11124235
rs7636293
rs4834232
rs11741331
rs7749507
rs10228542
rs6990940
rs7864646
rs4749500
rs10792136
rs957184
rs17121954
rs10152367
rs398465
rs7215873
rs12963780
rs17000836
rs2282053
rs2142842

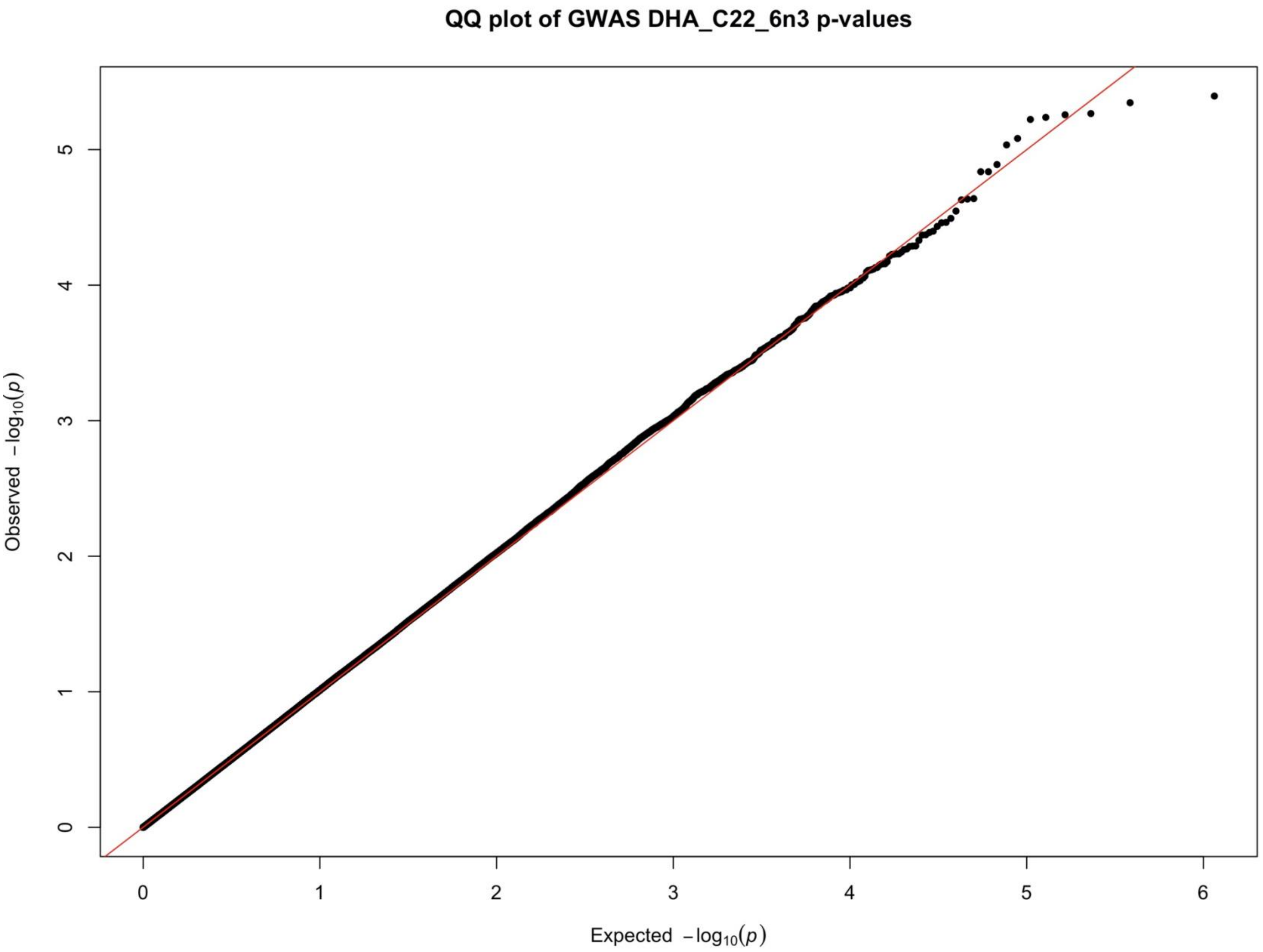
QQ plot of GWAS DHA_C22_6n3 p-values
Observed $-\log_{10}(p)$
Expected $-\log_{10}(p)$
0
1
2
3
4
5
6

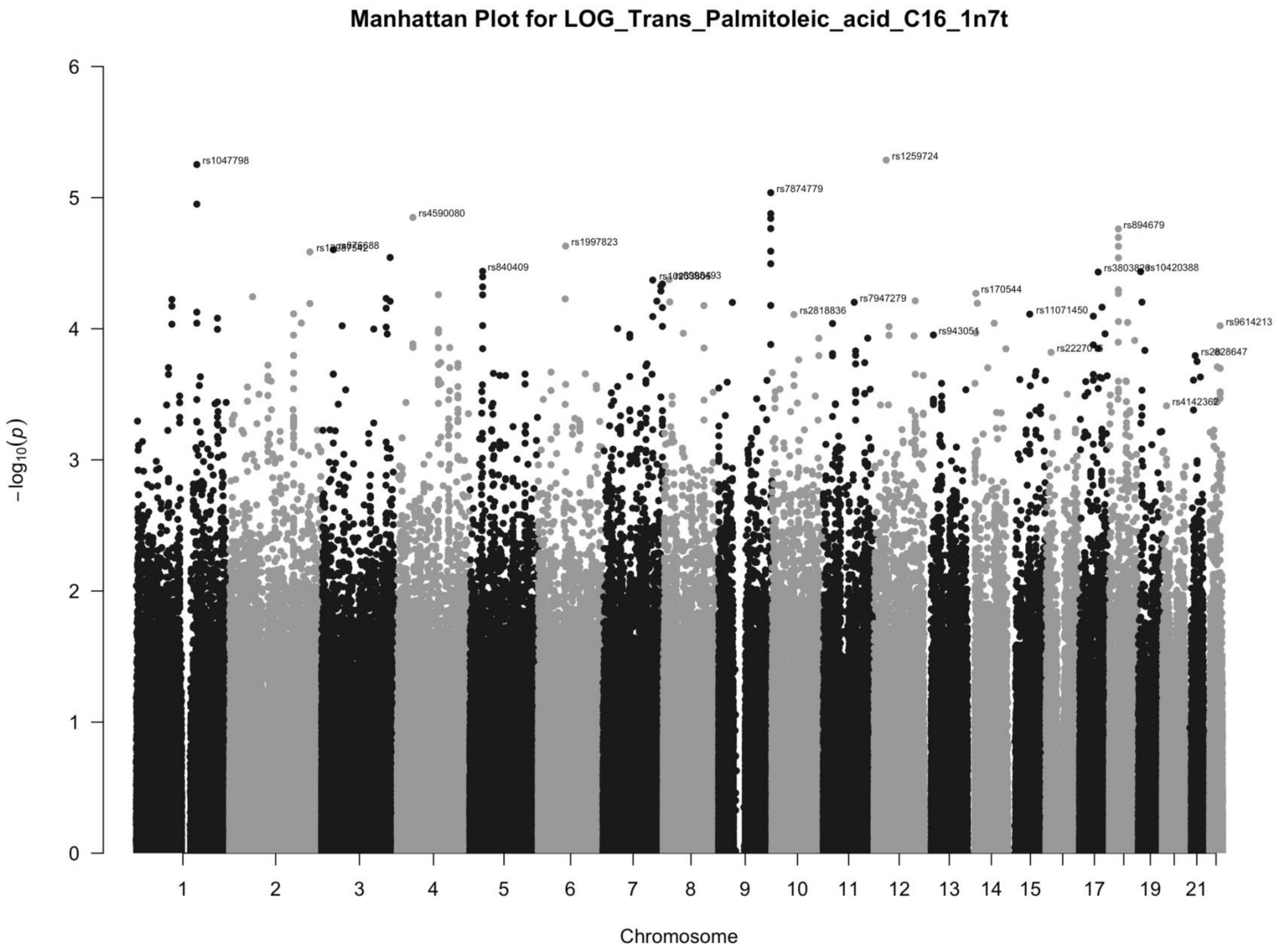
Manhattan Plot for LOG_Trans_Palmitoleic_acid_C16_1n7t
−log10(p)
Chromosome
rs1047798
rs1259724
rs7874779
rs4590080
rs894679
rs1997823
rs840409
rs3803826
rs10420388
rs170544
rs7947279
rs2818836
rs11071450
rs943051
rs9614213
rs4142362

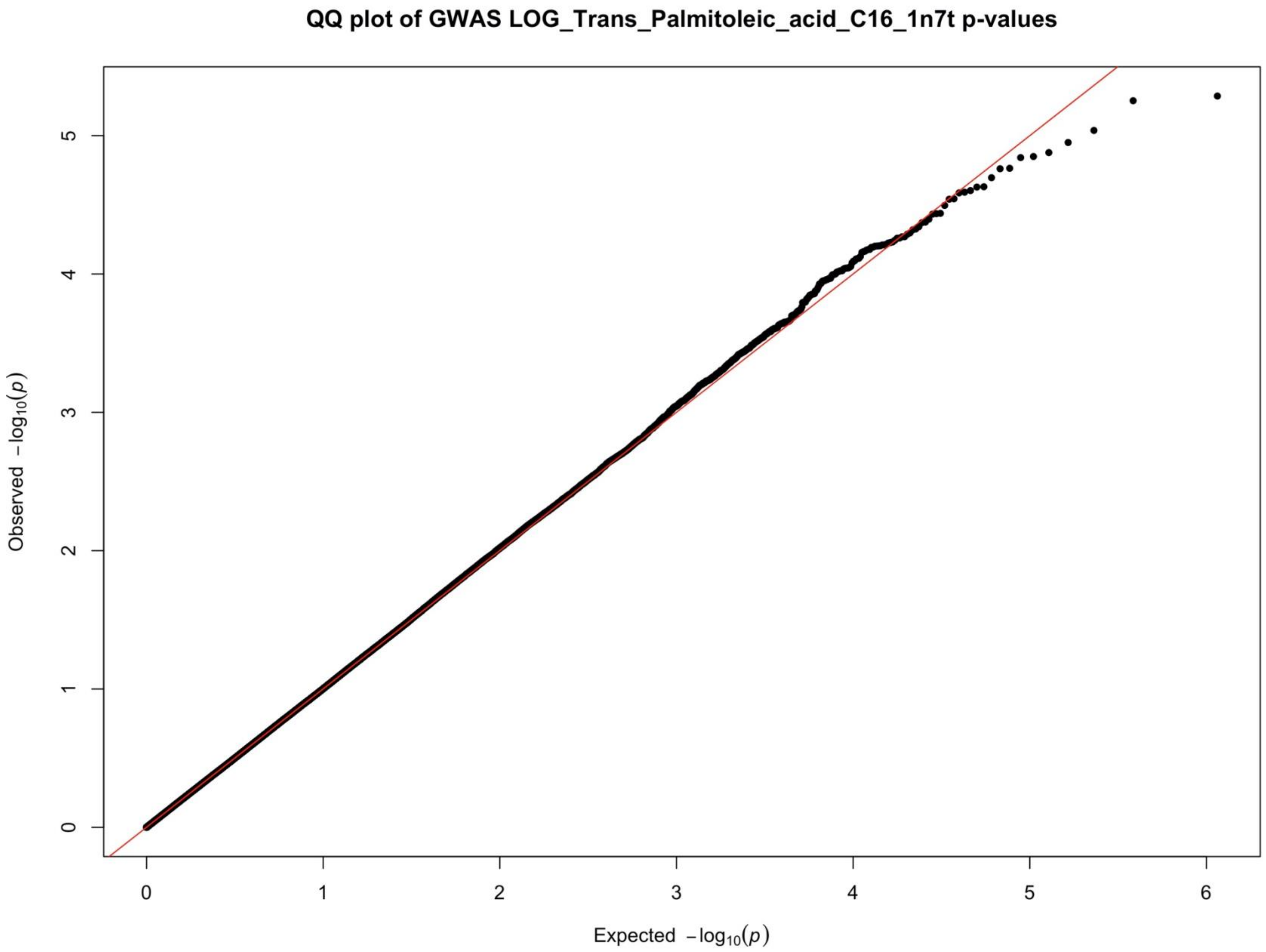
QQ plot of GWAS LOG_Trans_Palmitoleic_acid_C16_1n7t p-values
Observed −log10(p)
Expected −log10(p)
0
1
2
3
4
5
6

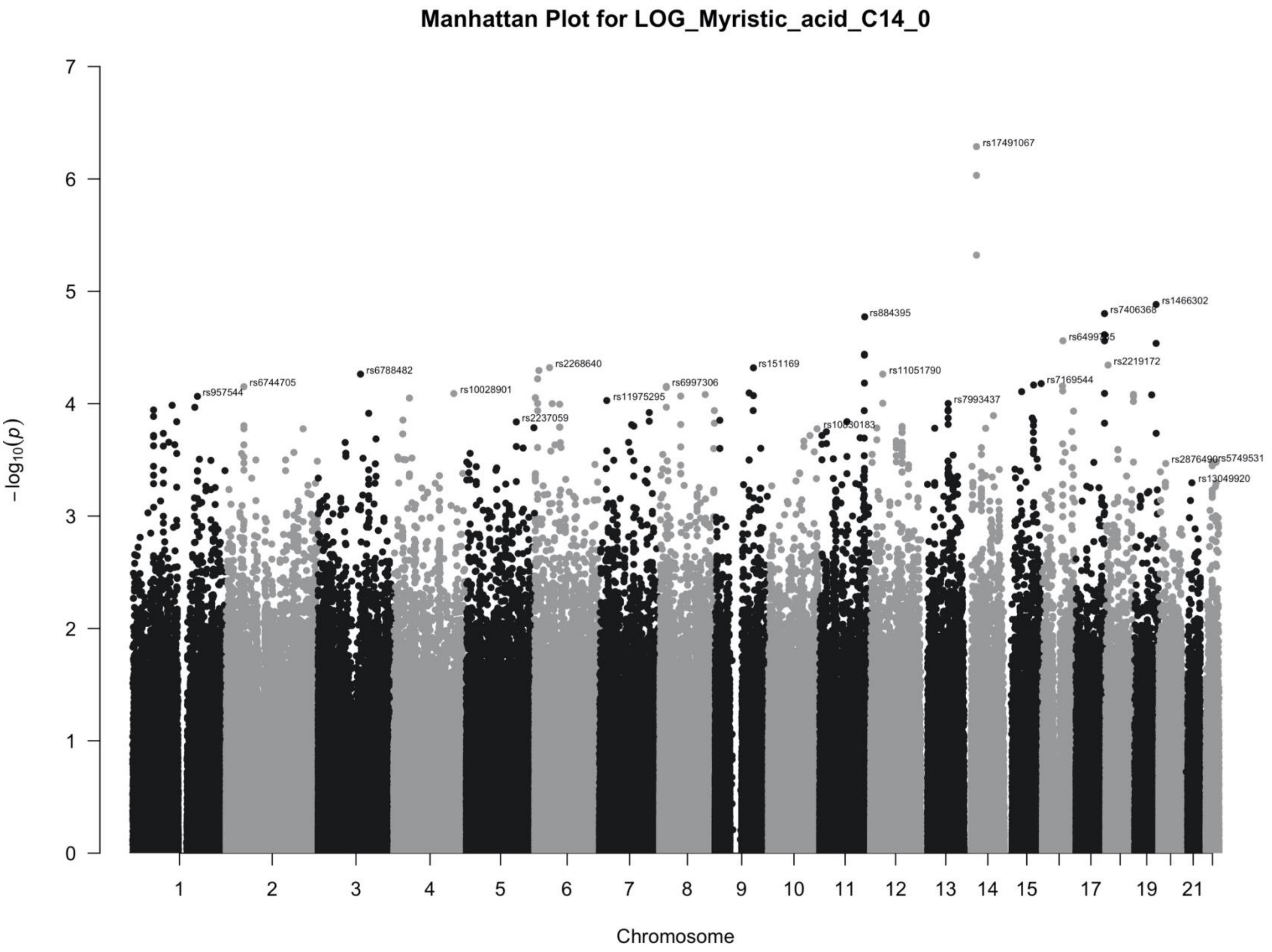
Manhattan Plot for LOG_Myristic_acid_C14_0
−log10(p)
Chromosome
0
1
2
3
4
5
6
7
1
2
3
4
5
6
7
8
9
10
11
12
13
14
15
17
19
21
rs17491067
rs1466302
rs7406368
rs884395
rs2219172
rs151169
rs2268640
rs11051790
rs6788482
rs6997306
rs7169544
rs6744705
rs957544
rs10028901
rs11975295
rs7993437
rs2237059
rs5749531
rs13049920

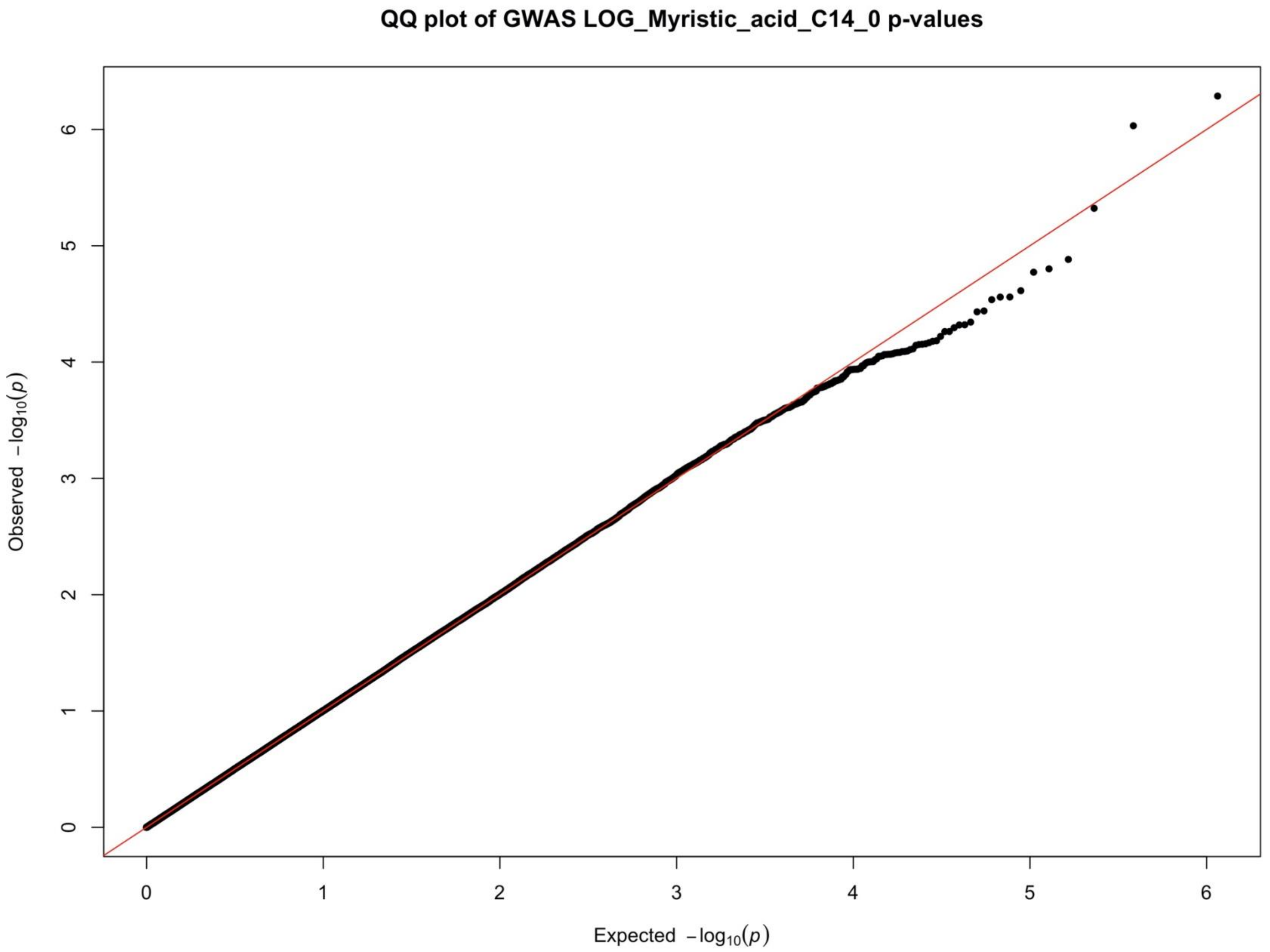
QQ plot of GWAS LOG_Myristic_acid_C14_0 p-values
Observed −log10(p)
Expected −log10(p)
0
1
2
3
4
5
6

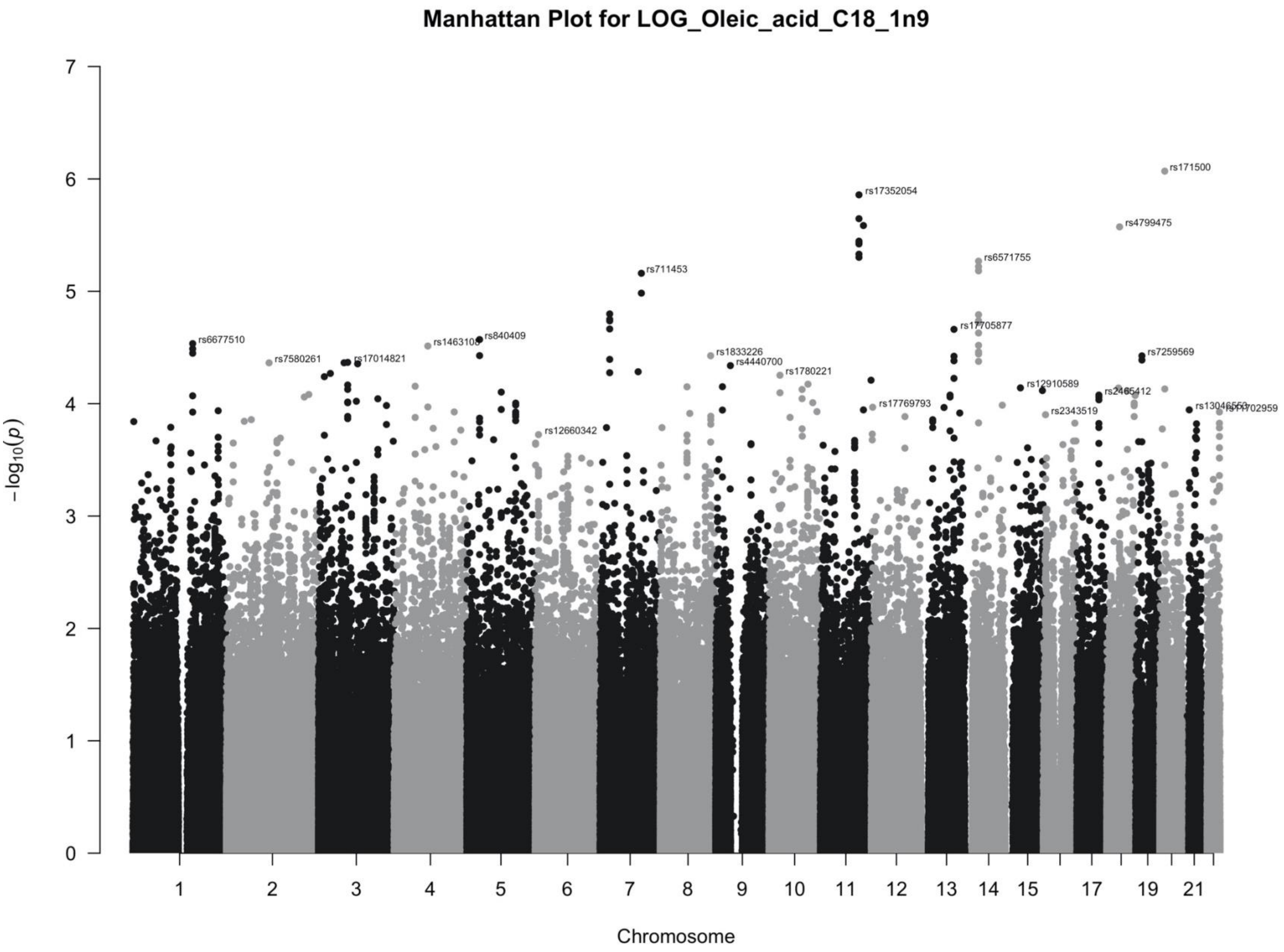
Manhattan Plot for LOG_Oleic_acid_C18_1n9
−log10(p)
7
6
5
4
3
2
1
0
Chromosome
1
2
3
4
5
6
7
8
9
10
11
12
13
14
15
17
19
21
rs171500
rs17352054
rs4799475
rs6571755
rs711453
rs17705877
rs6677510
rs840409
rs7580261
rs17014821
rs1833226
rs4440700
rs7259569
rs1780221
rs12910589
rs2465412
rs17769793
rs2343519
rs12660342

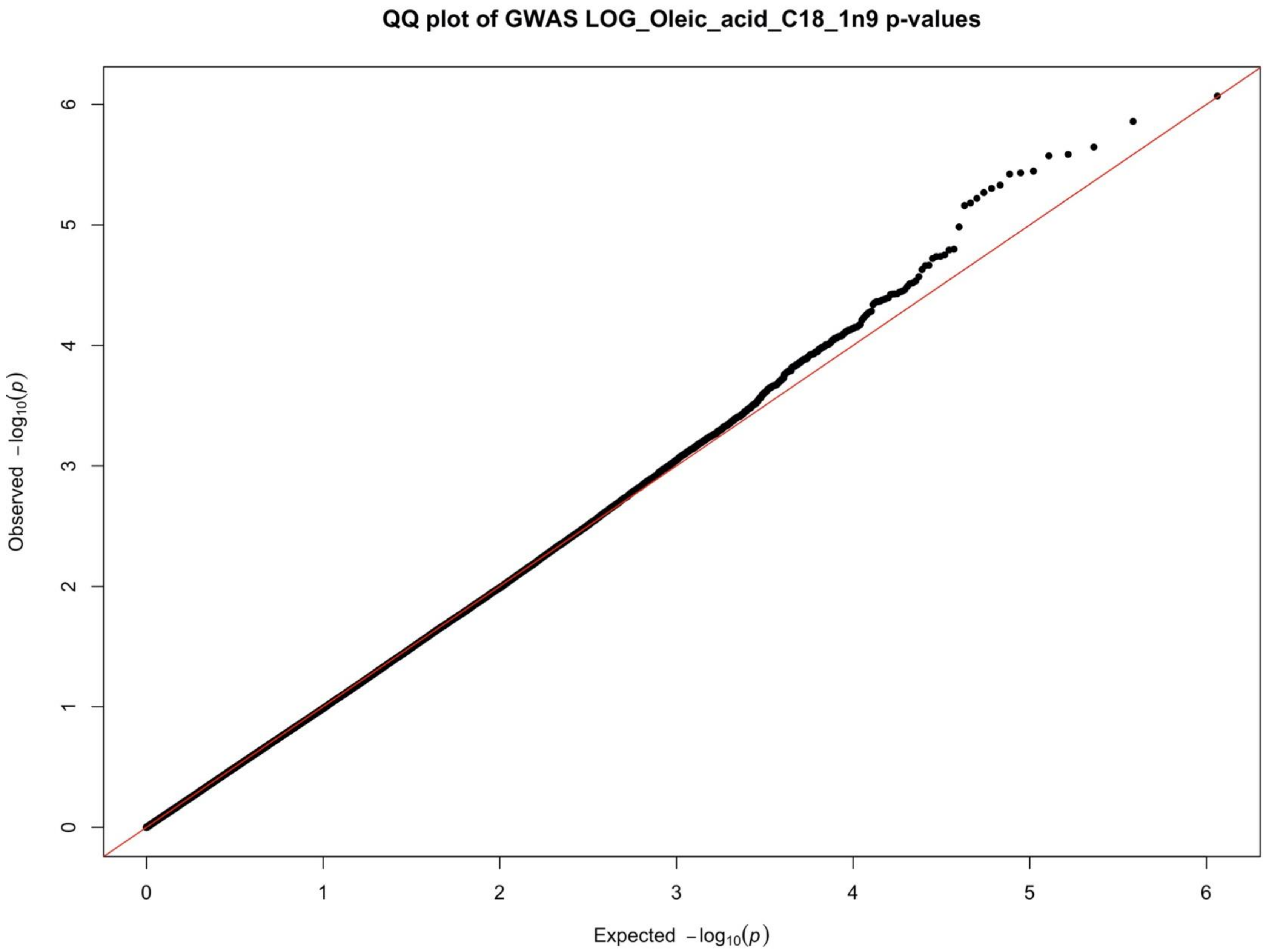
QQ plot of GWAS LOG_Oleic_acid_C18_1n9 p-values
Observed −log10(p)
Expected −log10(p)
0
1
2
3
4
5
6

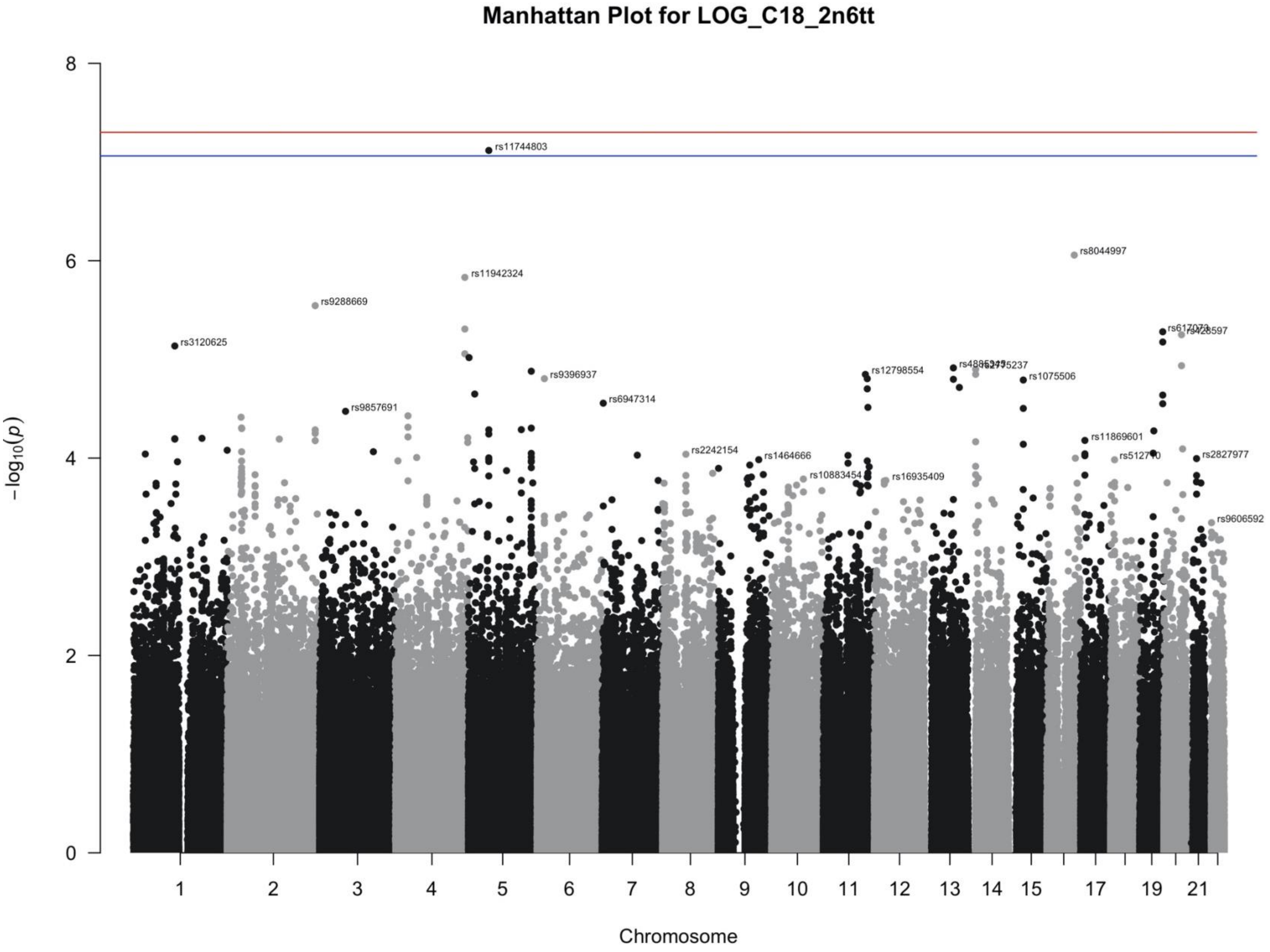
Manhattan Plot for LOG_C18_2n6tt
$-\log_{10}(p)$
8
6
4
2
0
rs11744803
rs8044997
rs11942324
rs9288669
rs3120625
rs9396937
rs12798554
rs1075506
rs6947314
rs9857691
rs11869601
rs2242154
rs1464666
rs10883454
rs16935409
rs2827977
rs9606592
1
2
3
4
5
6
7
8
9
10
11
12
13
14
15
17
19
21
Chromosome

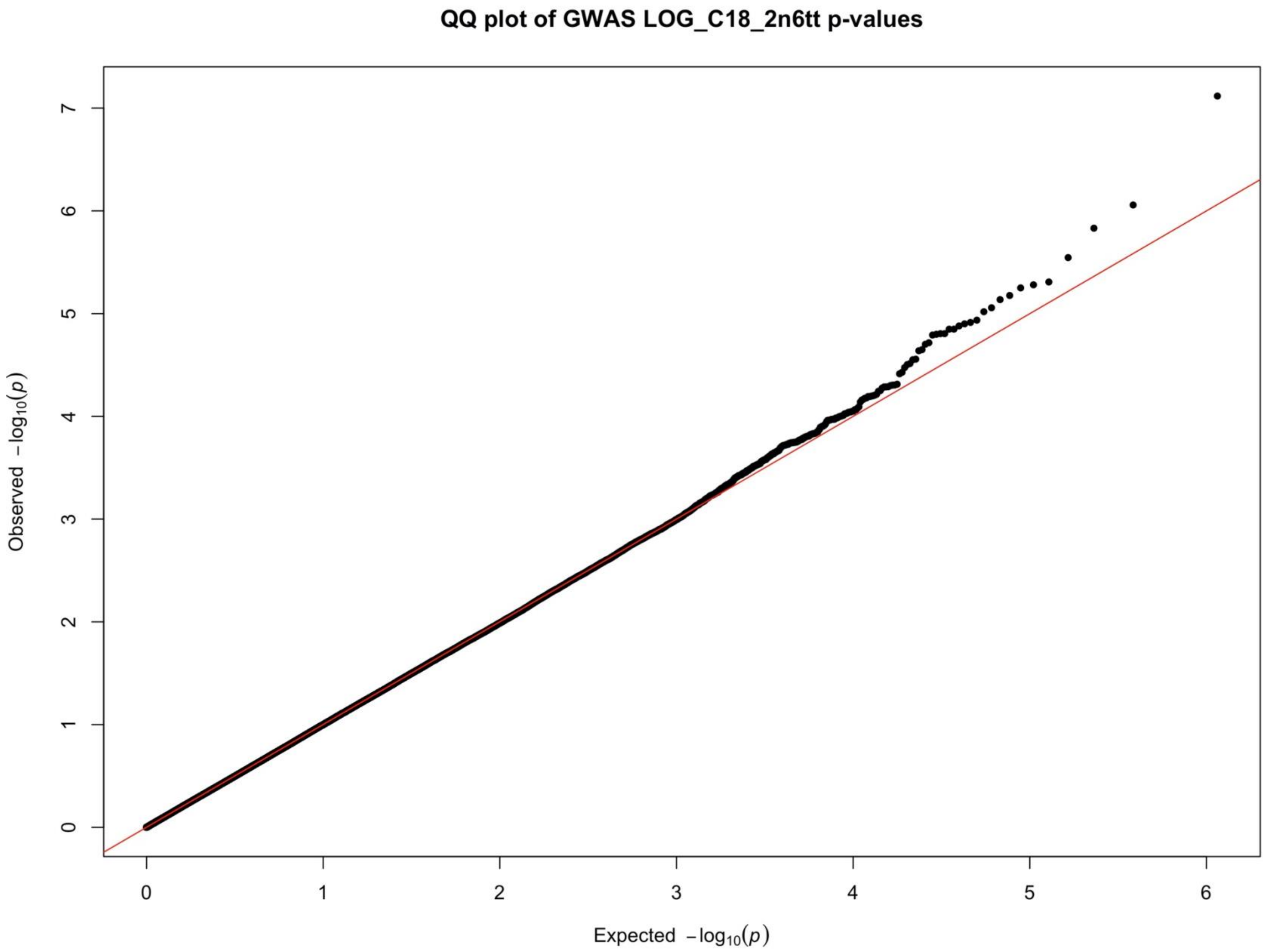
QQ plot of GWAS LOG_C18_2n6tt p-values
Observed −log10(p)
Expected −log10(p)
0
1
2
3
4
5
6
7

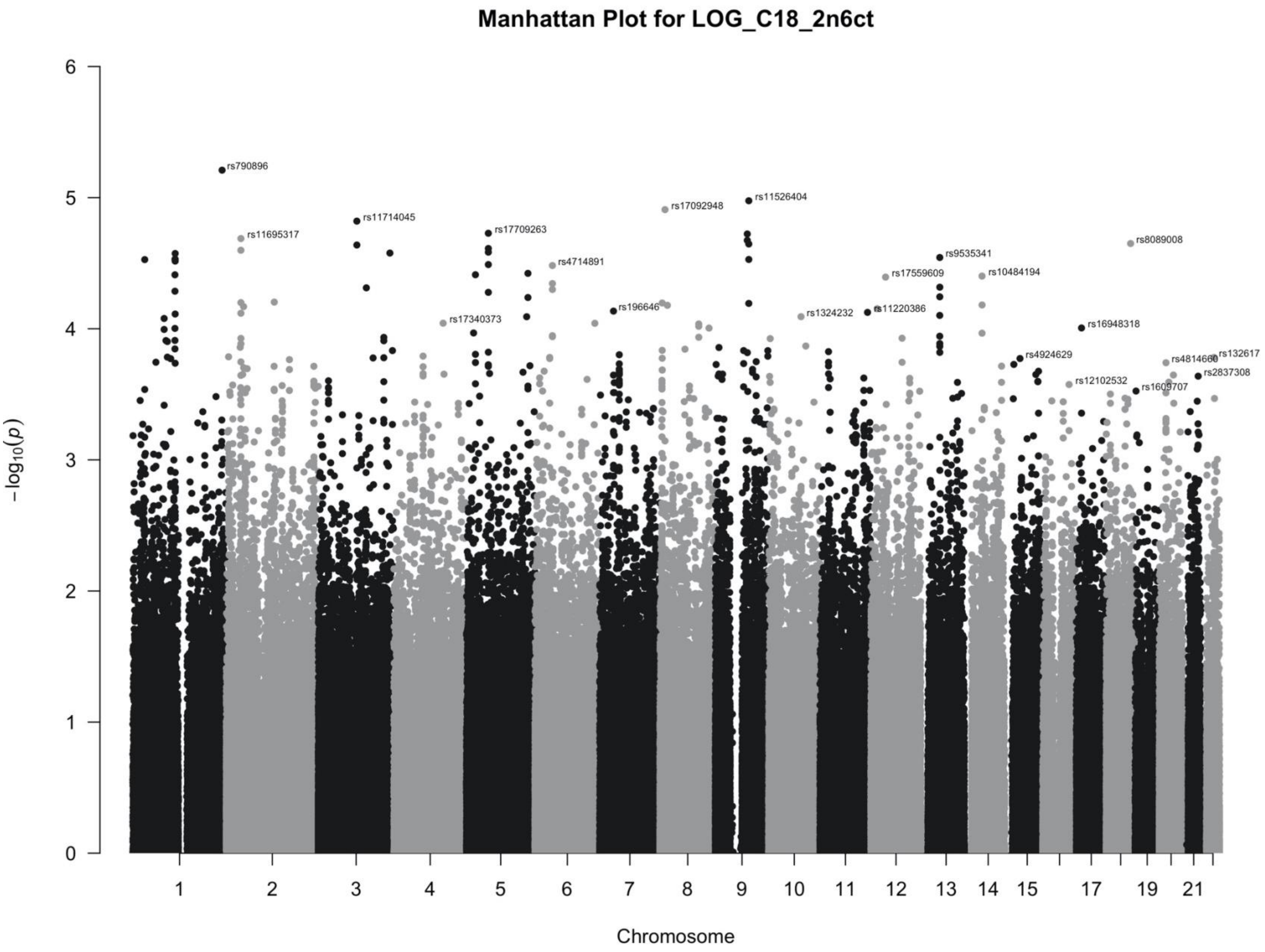
Manhattan Plot for LOG_C18_2n6ct
−log10(p)
0
1
2
3
4
5
6
Chromosome
1
2
3
4
5
6
7
8
9
10
11
12
13
14
15
17
19
21
rs790896
rs11695317
rs11714045
rs17709263
rs4714891
rs17340373
rs196646
rs17092948
rs11526404
rs1324232
rs11220386
rs17559609
rs9535341
rs10484194
rs8089008
rs16948318
rs4924629
rs12102532
rs1609707
rs4814660
rs132617
rs2837308

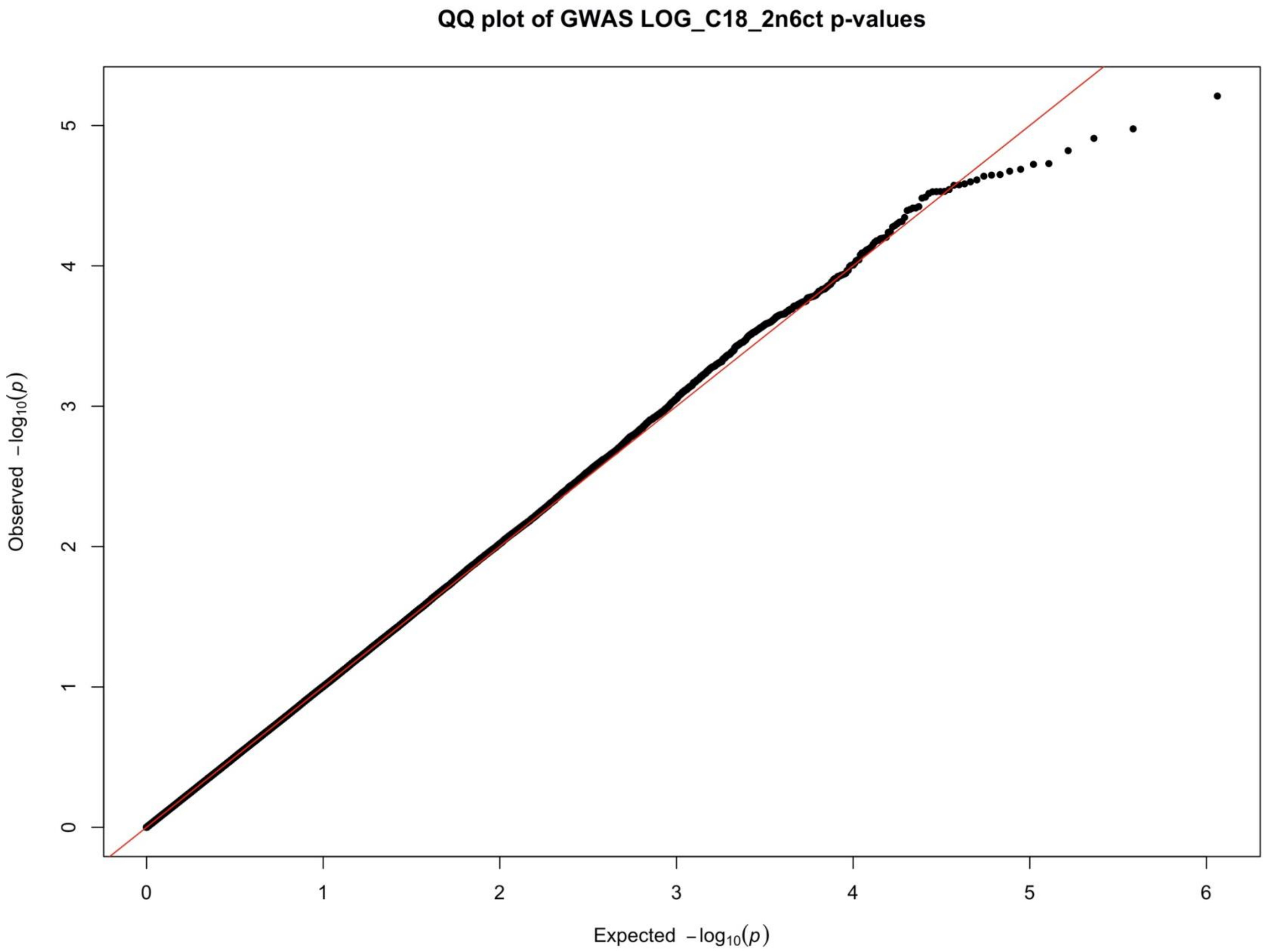
QQ plot of GWAS LOG_C18_2n6ct p-values
Observed −log10(p)
Expected −log10(p)
0
1
2
3
4
5
6

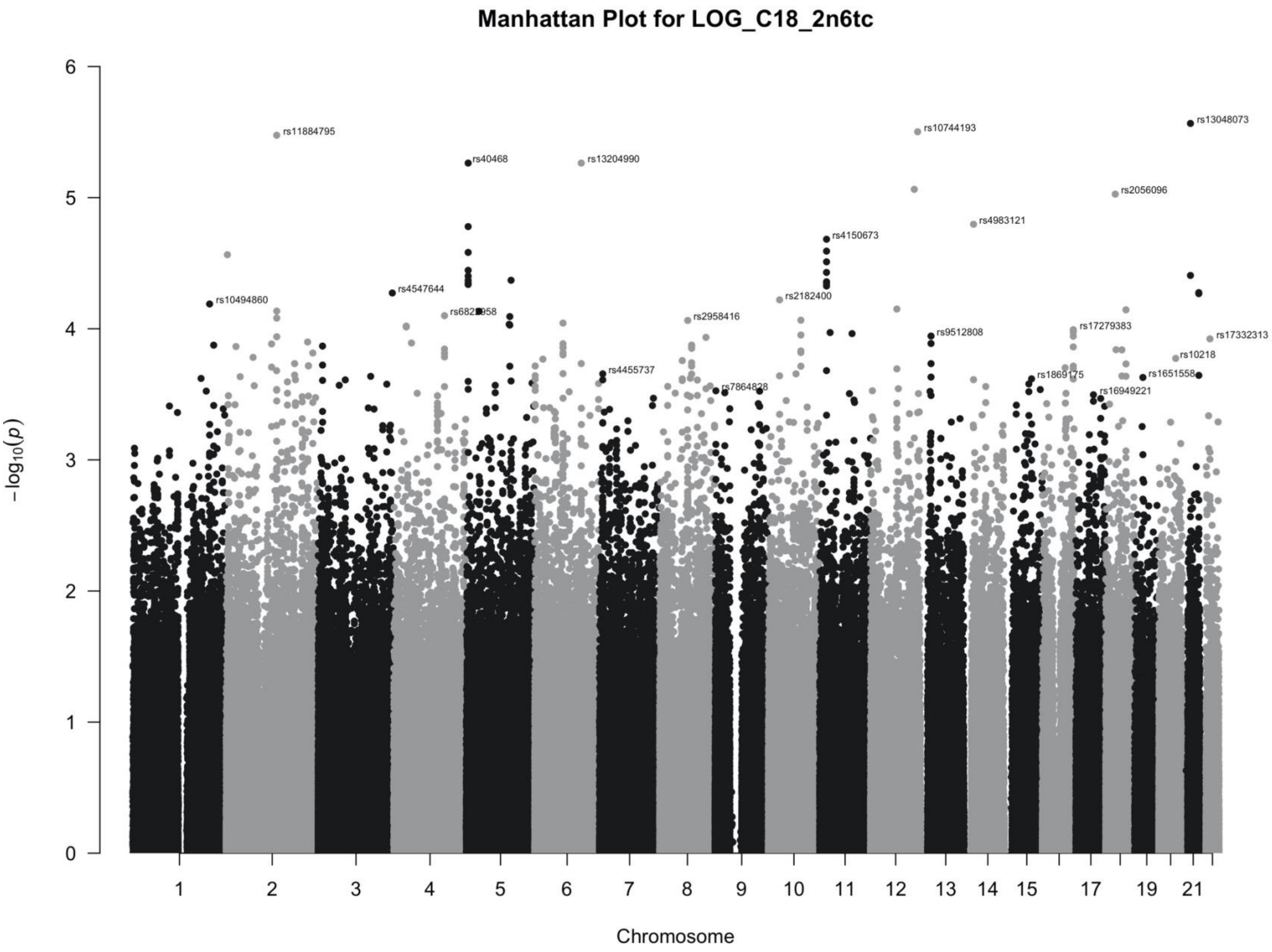
Manhattan Plot for LOG_C18_2n6tc
-log10(p)
0
1
2
3
4
5
6
Chromosome
1
2
3
4
5
6
7
8
9
10
11
12
13
14
15
17
19
21
rs11884795
rs40468
rs13204990
rs10744193
rs13048073
rs2056096
rs4983121
rs4150673
rs10494860
rs4547644
rs2182400
rs2958416
rs9512808
rs17279383
rs17332313
rs10218
rs4455737
rs7864828
rs1869175
rs1651558
rs16949221

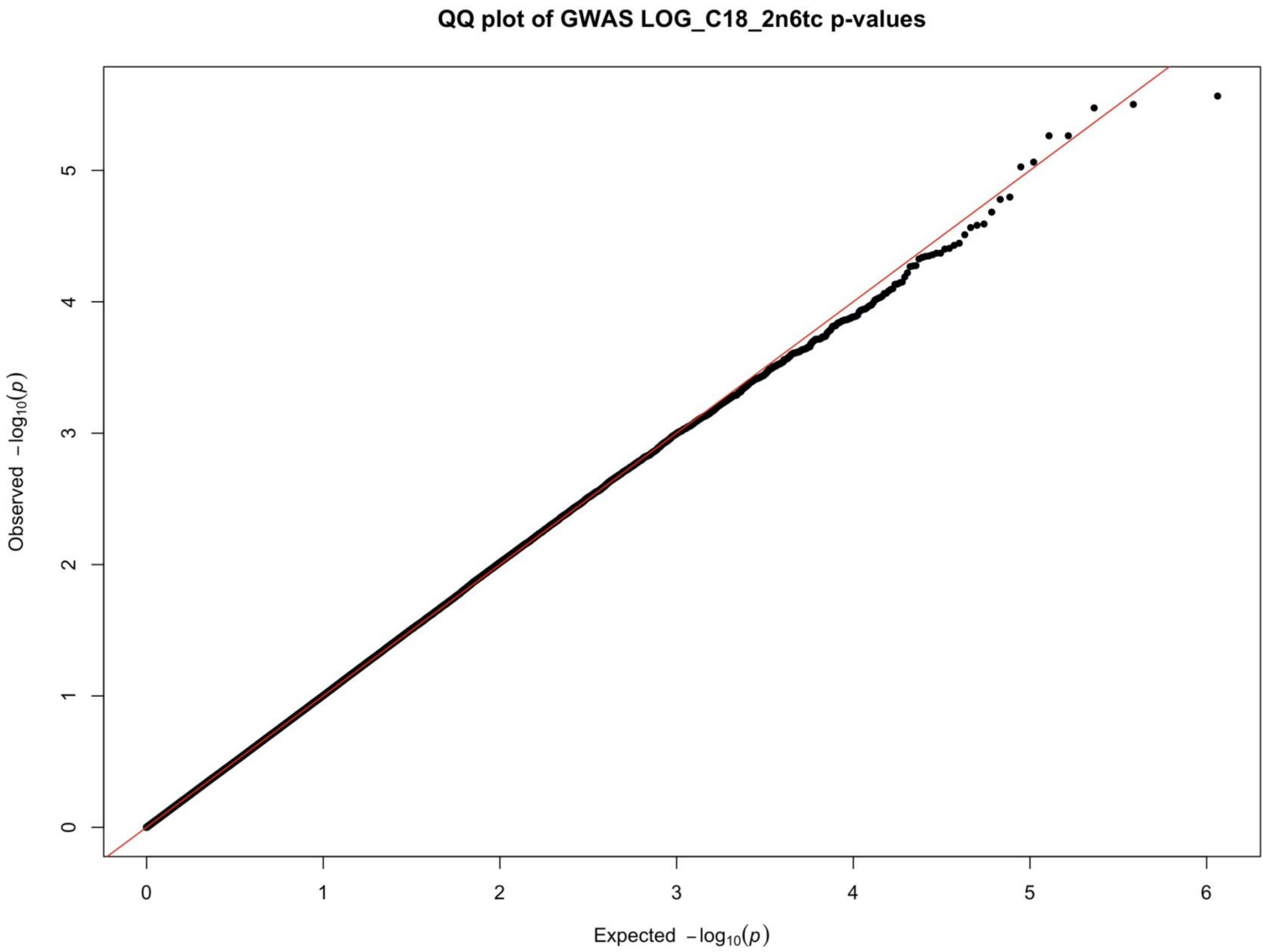
QQ plot of GWAS LOG_C18_2n6tc p-values
Observed −log10(p)
Expected −log10(p)
0
1
2
3
4
5
6

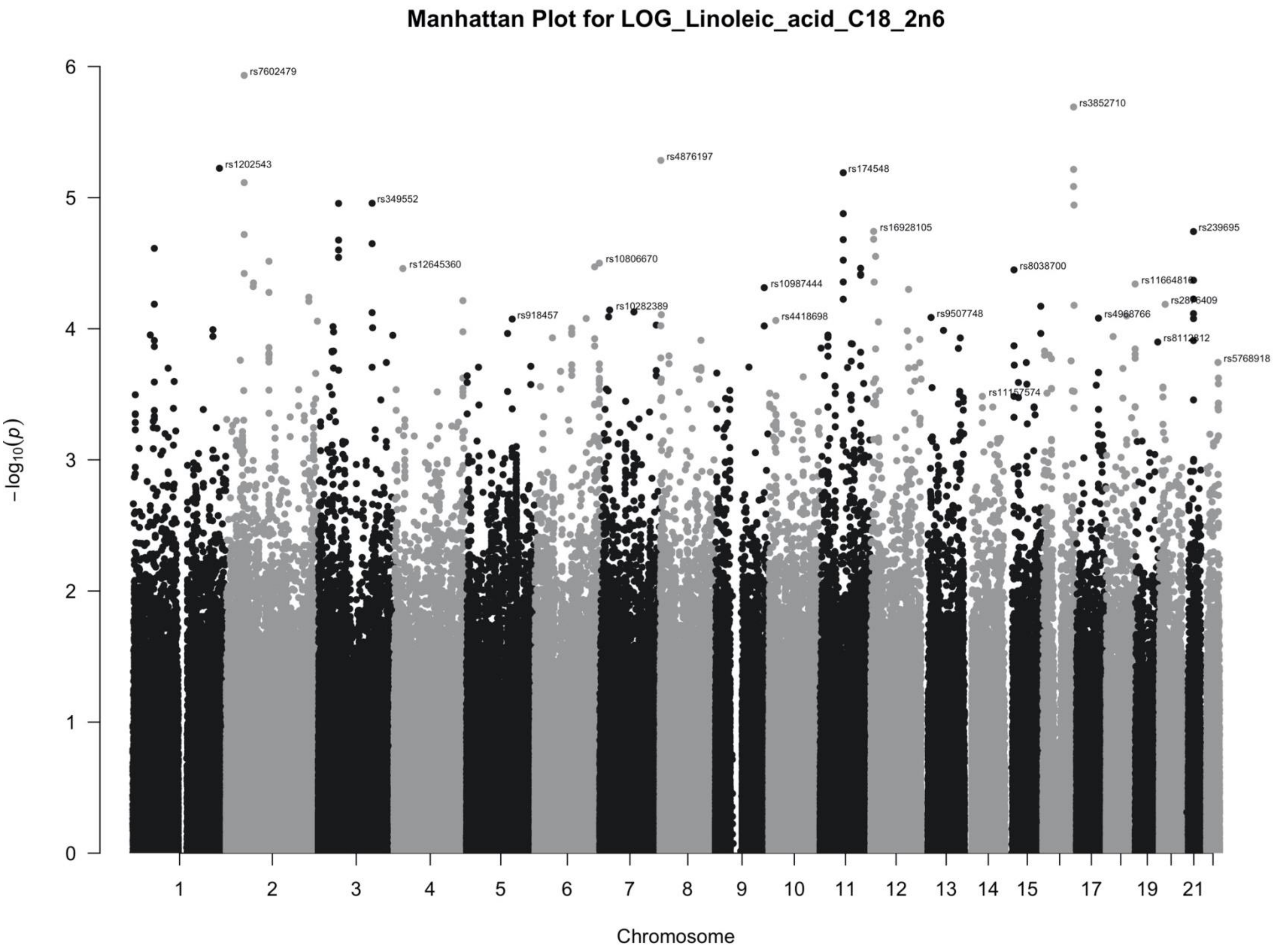
Manhattan Plot for LOG_Linoleic_acid_C18_2n6
−log10(p)
Chromosome
0
1
2
3
4
5
6
1
2
3
4
5
6
7
8
9
10
11
12
13
14
15
17
19
21
rs7602479
rs3852710
rs1202543
rs4876197
rs174548
rs349552
rs16928105
rs239695
rs12645360
rs10806670
rs8038700
rs10987444
rs10282389
rs918457
rs4418698
rs9507748
rs4968766
rs5768918
rs11157574

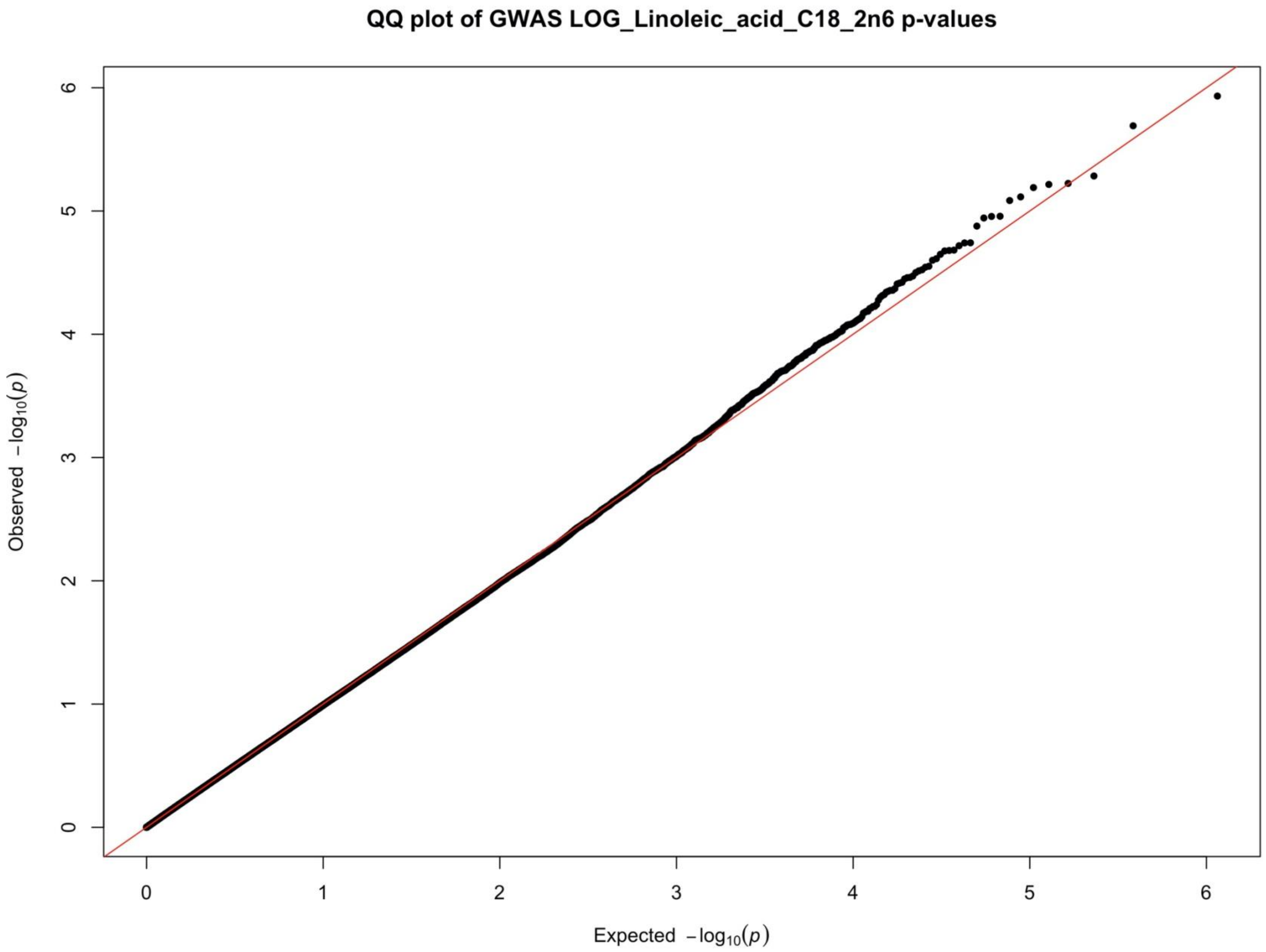
QQ plot of GWAS LOG_Linoleic_acid_C18_2n6 p-values
Observed −log10(p)
Expected −log10(p)
0
1
2
3
4
5
6

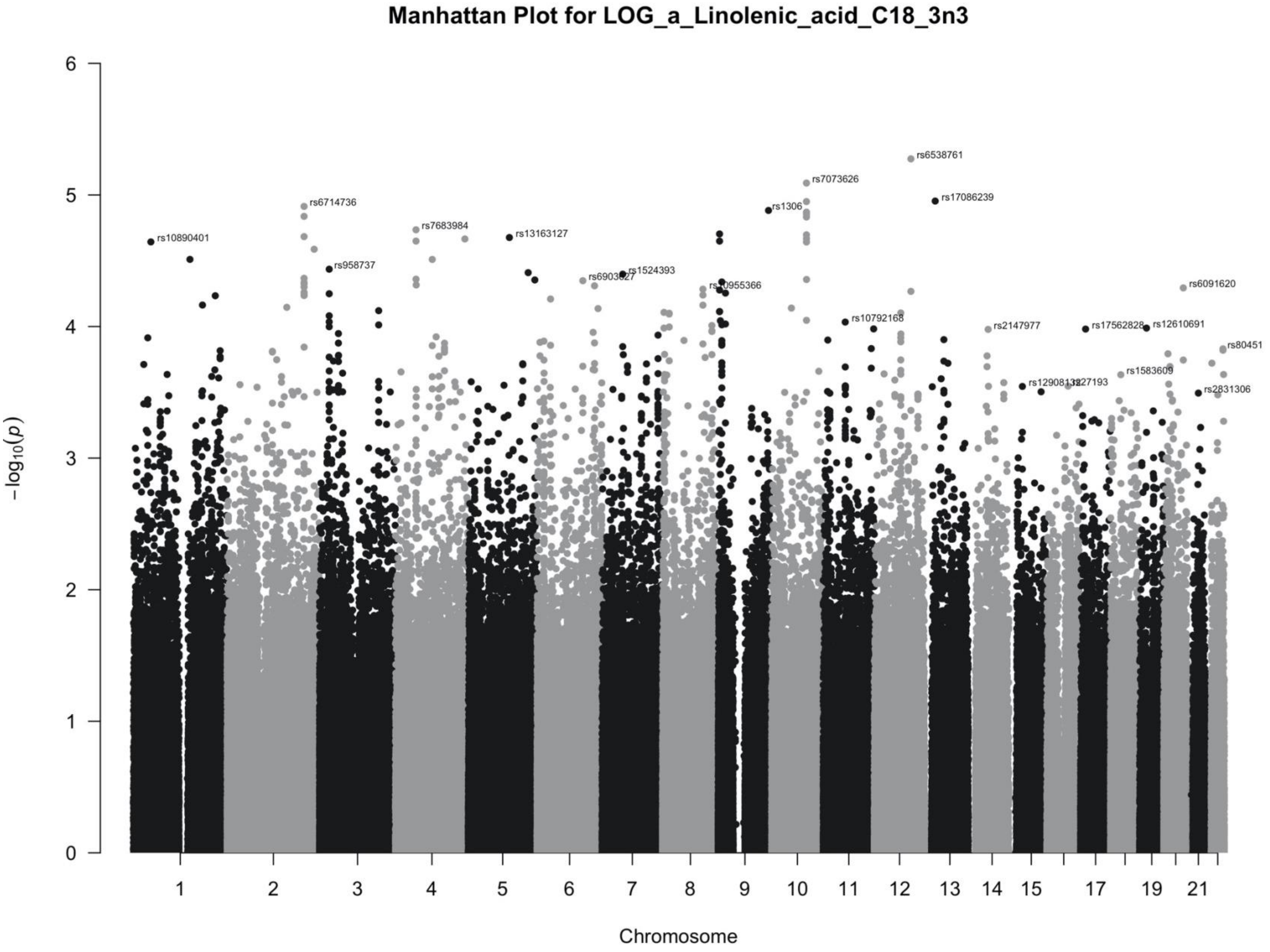
Manhattan Plot for LOG_a_Linolenic_acid_C18_3n3
−log10(p)
0
1
2
3
4
5
6
Chromosome
1
2
3
4
5
6
7
8
9
10
11
12
13
14
15
17
19
21
rs10890401
rs6714736
rs958737
rs7683984
rs13163127
rs1524393
rs1306
rs7073626
rs6538761
rs17086239
rs10792168
rs2147977
rs17562828
rs12610691
rs1583609
rs6091620
rs80451
rs2831306

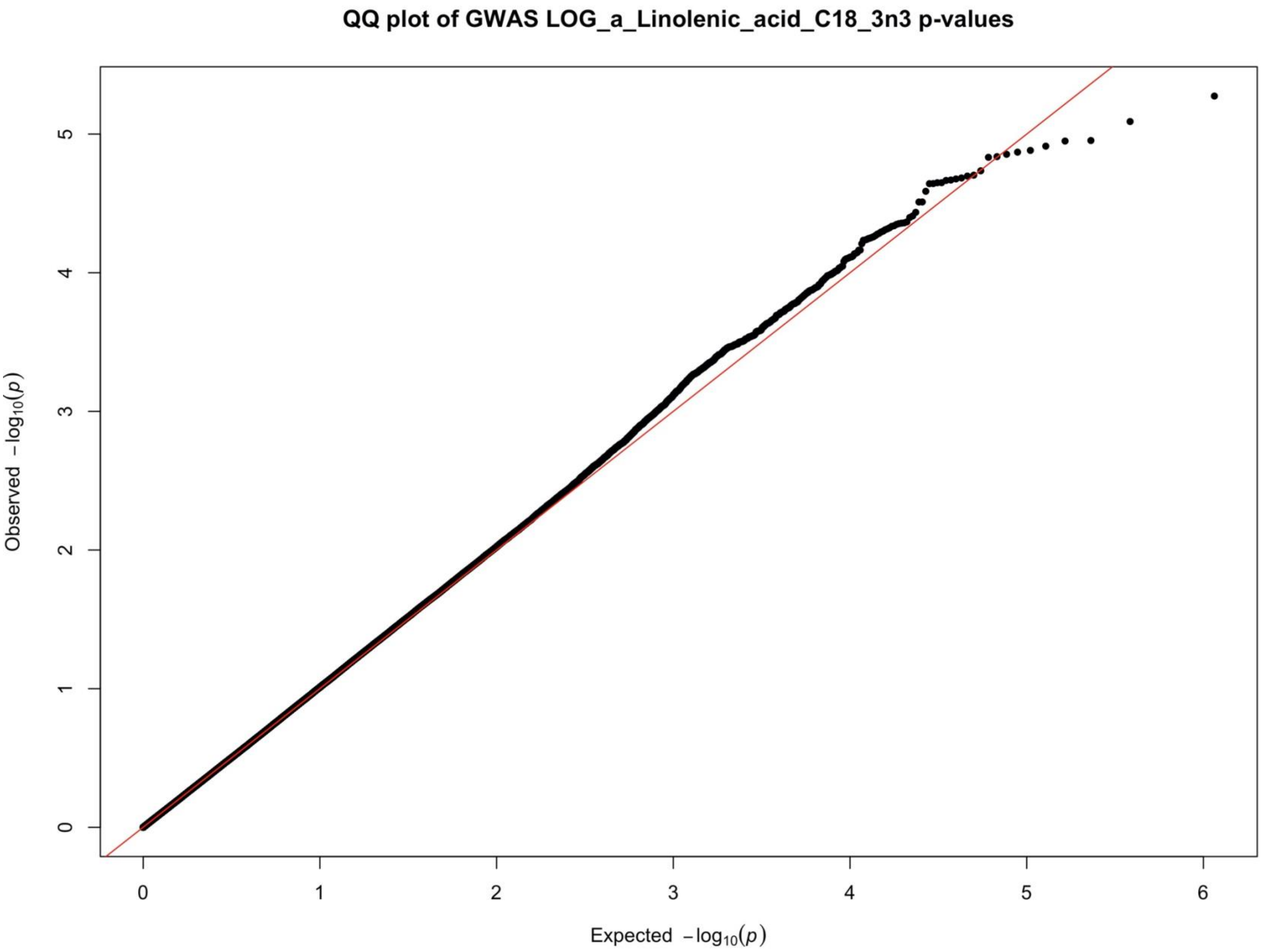
QQ plot of GWAS LOG_a_Linolenic_acid_C18_3n3 p-values
Observed −log10(p)
Expected −log10(p)
0
1
2
3
4
5
6

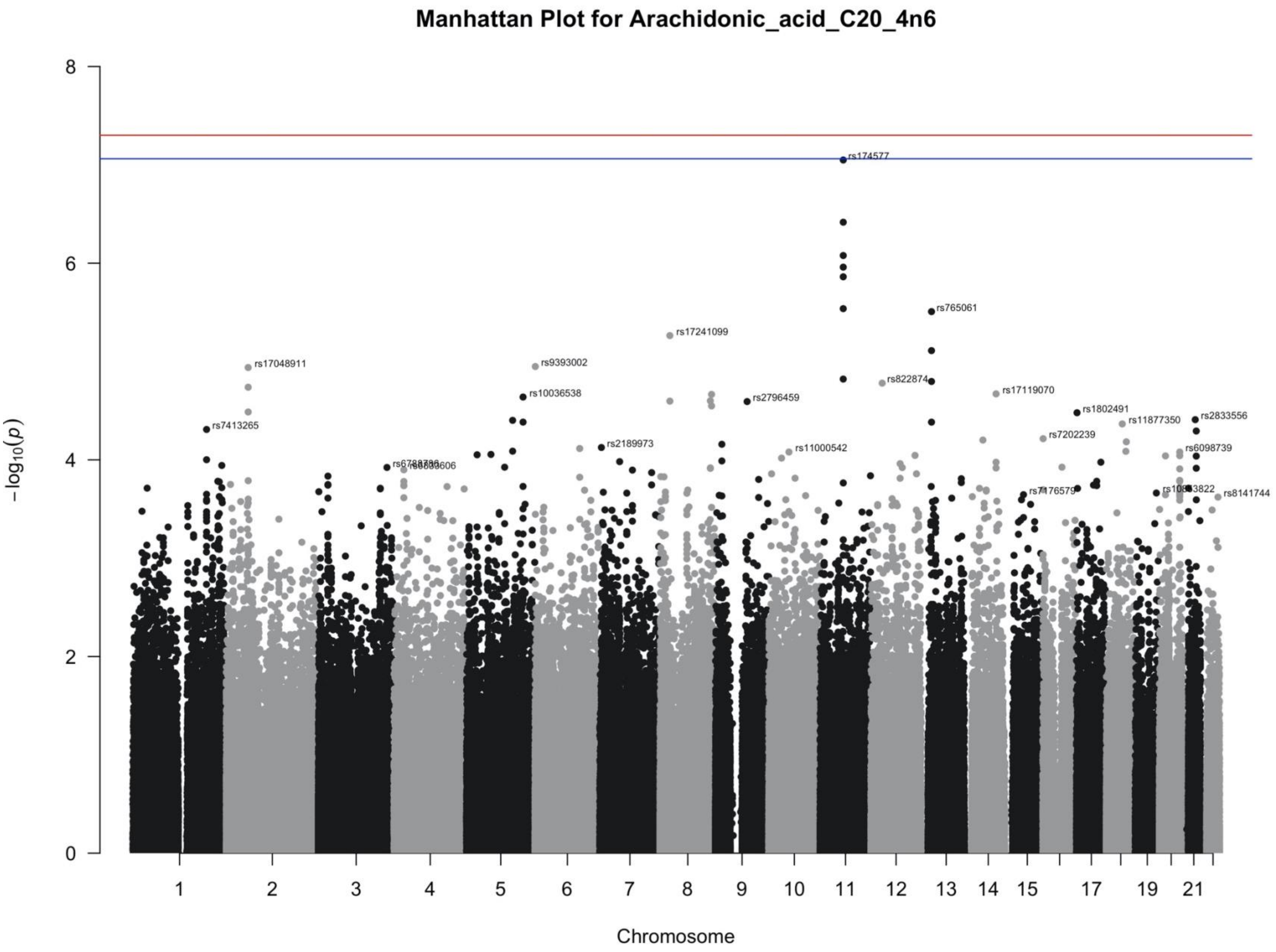
Manhattan Plot for Arachidonic_acid_C20_4n6
8
6
4
2
0
−log10(p)
rs174577
rs765061
rs17241099
rs17048911
rs9393002
rs10036538
rs822874
rs17119070
rs2796459
rs7413265
rs1802491
rs2833556
rs11877350
rs7202239
rs2189973
rs11000542
rs6098739
rs8141744
1
2
3
4
5
6
7
8
9
10
11
12
13
14
15
17
19
21
Chromosome

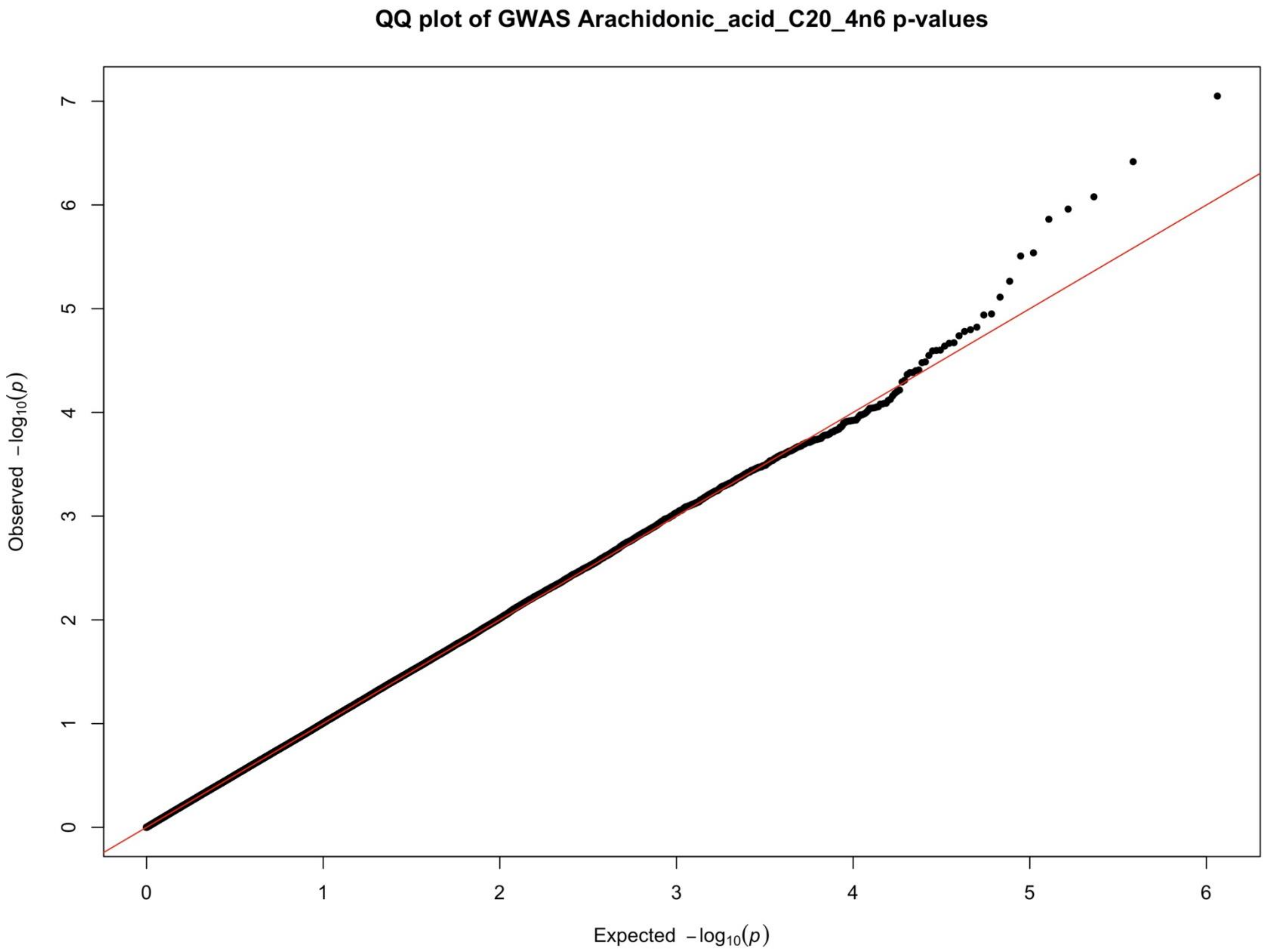
QQ plot of GWAS Arachidonic_acid_C20_4n6 p-values
Observed −log10(p)
Expected −log10(p)
0
1
2
3
4
5
6
7
0
1
2
3
4
5
6

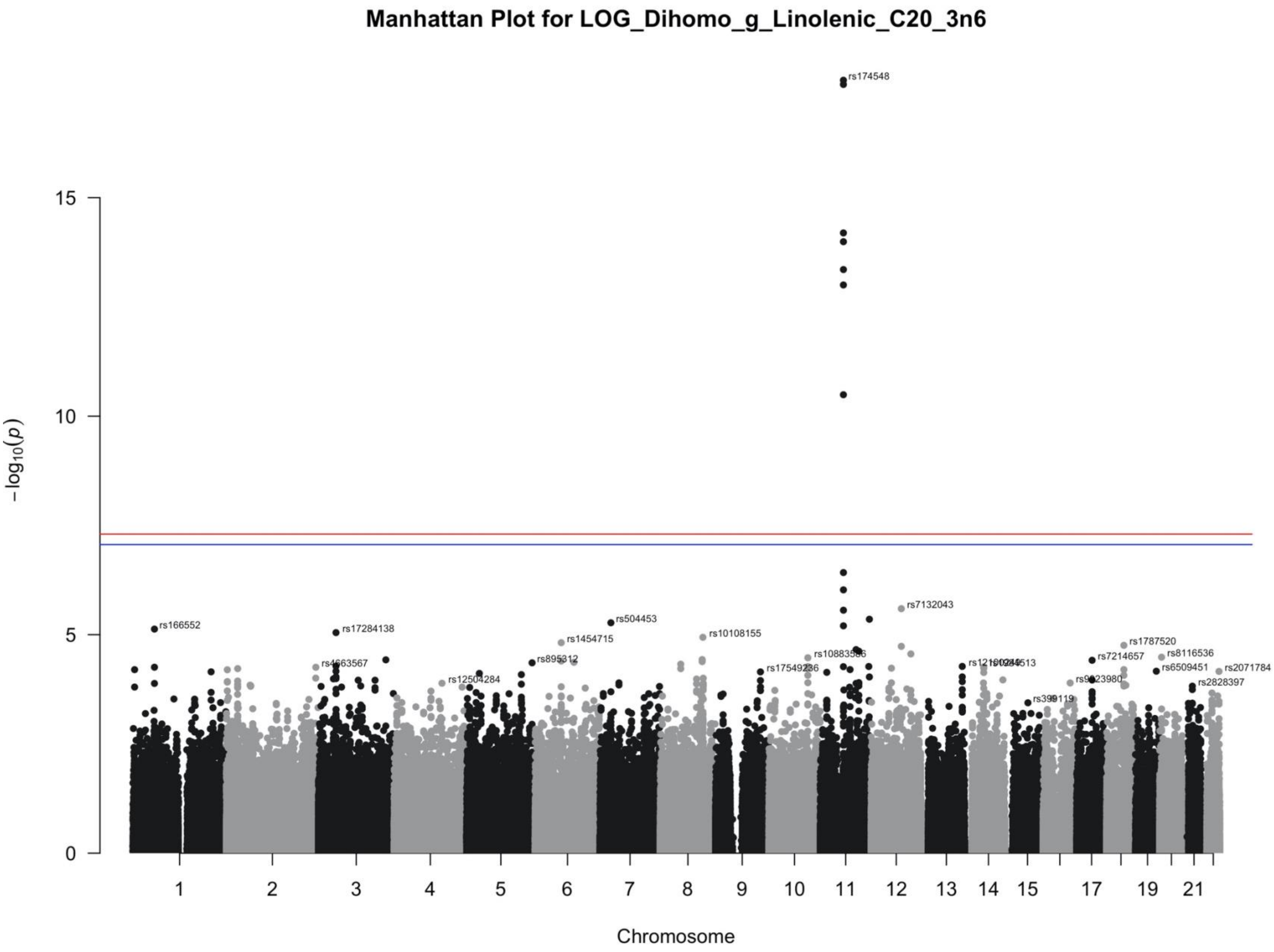
Manhattan Plot for LOG_Dihomo_g_Linolenic_C20_3n6
rs174548
rs166552
rs17284138
rs504453
rs1454715
rs10108155
rs7132043
rs1787520
rs8116536
rs7214657
rs6509451
rs2071784
rs2828397
rs17549236
rs12504284
−log10(p)
15
10
5
0
1
2
3
4
5
6
7
8
9
10
11
12
13
14
15
17
19
21
Chromosome

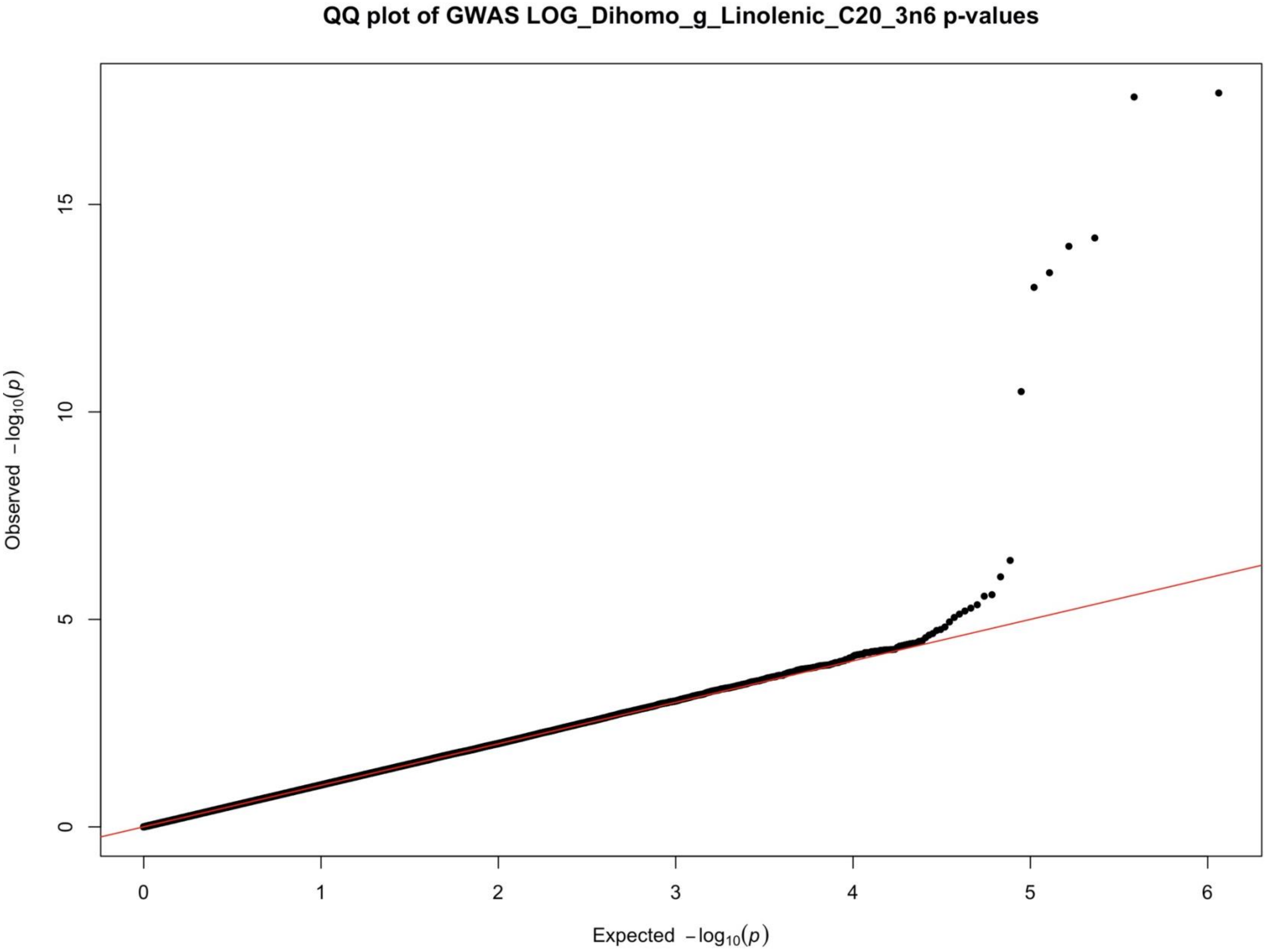
QQ plot of GWAS LOG_Dihomo_g_Linolenic_C20_3n6 p-values
Observed −log10(p)
Expected −log10(p)
0
5
10
15
0
1
2
3
4
5
6

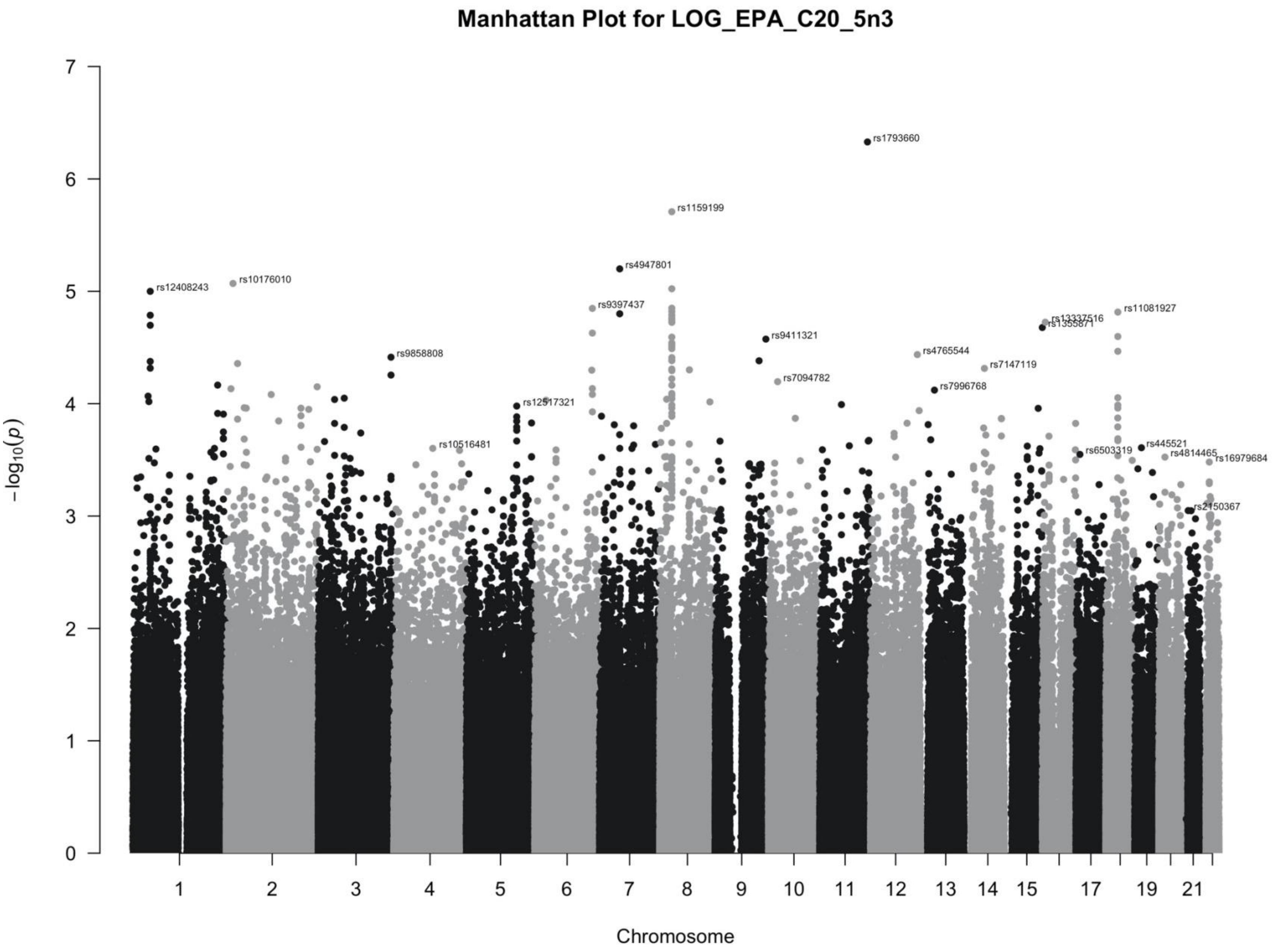
Manhattan Plot for LOG_EPA_C20_5n3
−log10(p)
Chromosome
0
1
2
3
4
5
6
7
1
2
3
4
5
6
7
8
9
10
11
12
13
14
15
17
19
21
rs1793660
rs1159199
rs4947801
rs12408243
rs10176010
rs9397437
rs11081927
rs9411321
rs4765544
rs9858808
rs7147119
rs7094782
rs7996768
rs12517321
rs10516481
rs6503319
rs445521
rs4814465
rs16979684
rs2150367

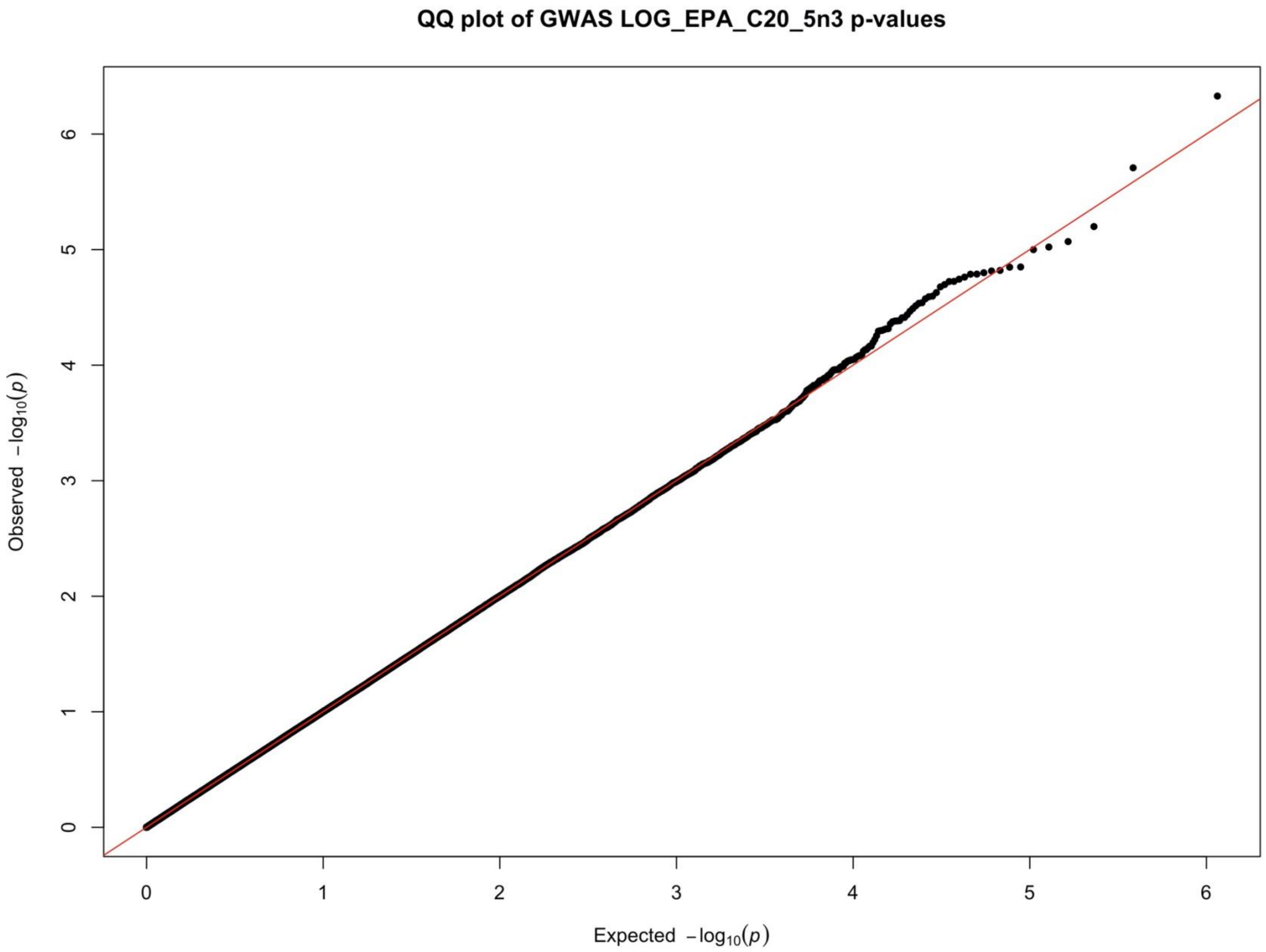
QQ plot of GWAS LOG_EPA_C20_5n3 p-values
Observed −log10(p)
Expected −log10(p)
0
1
2
3
4
5
6
