# Supplementary material for: Investigation of gene-gene interactions in cardiac traits and serum fatty acid levels in the LURIC Health Study
Source: PLoS One. 2020 Sep 11;15(9):e0238304. doi: 10.1371/journal.pone.0238304 (PMC7485803; doi:10.1371/journal.pone.0238304)
Supplement: S2 Fig — Genetic principal component analysis (PCA) results: A) Distribution of the first 20 PCs and B) PC1 against PC2. The genetic principal components were calculated based on the individual genetic information. PC1 explained 0.103% of genetic variations within the LURIC cohort and PC2 could explained 0.0674% of genetic variation within the LURIC cohort. (PDF) [file pone.0238304.s002.pdf]

S2 Fig. Genetic principal component analysis (PCA) results: A) Distribution of the first 20 PCs and B) PC1 against PC2. The genetic principal components were calculated based on the individual genetic information. The PC1 could explain 0.103% of genetic variations within the LURIC cohort and the PC2 could explain 0.0674% of genetic variation within the LURIC cohort.

A)

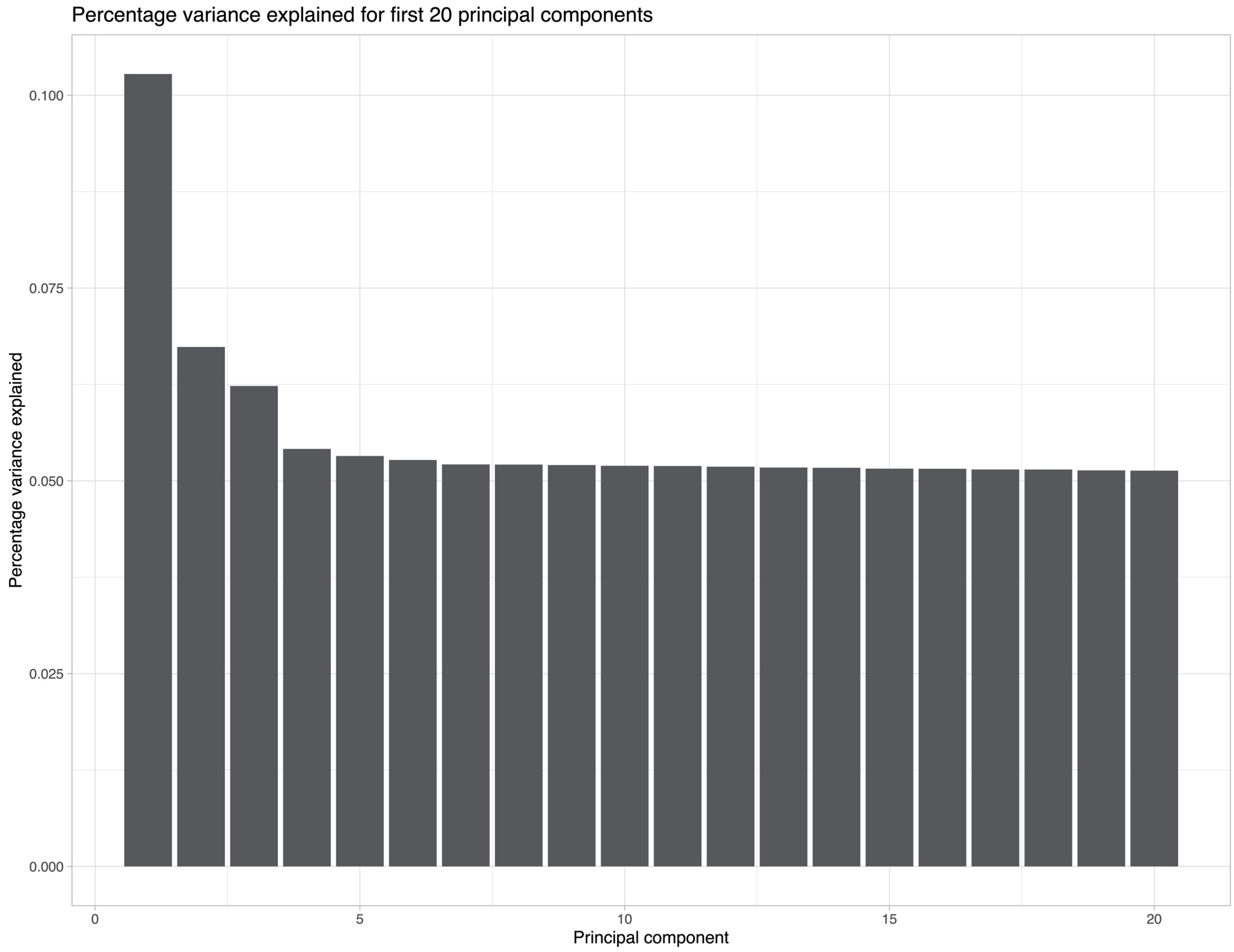

Percentage variance explained for first 20 principal components
Percentage variance explained
0.100
0.075
0.050
0.025
0.000
0
5
10
15
20
Principal component

B)

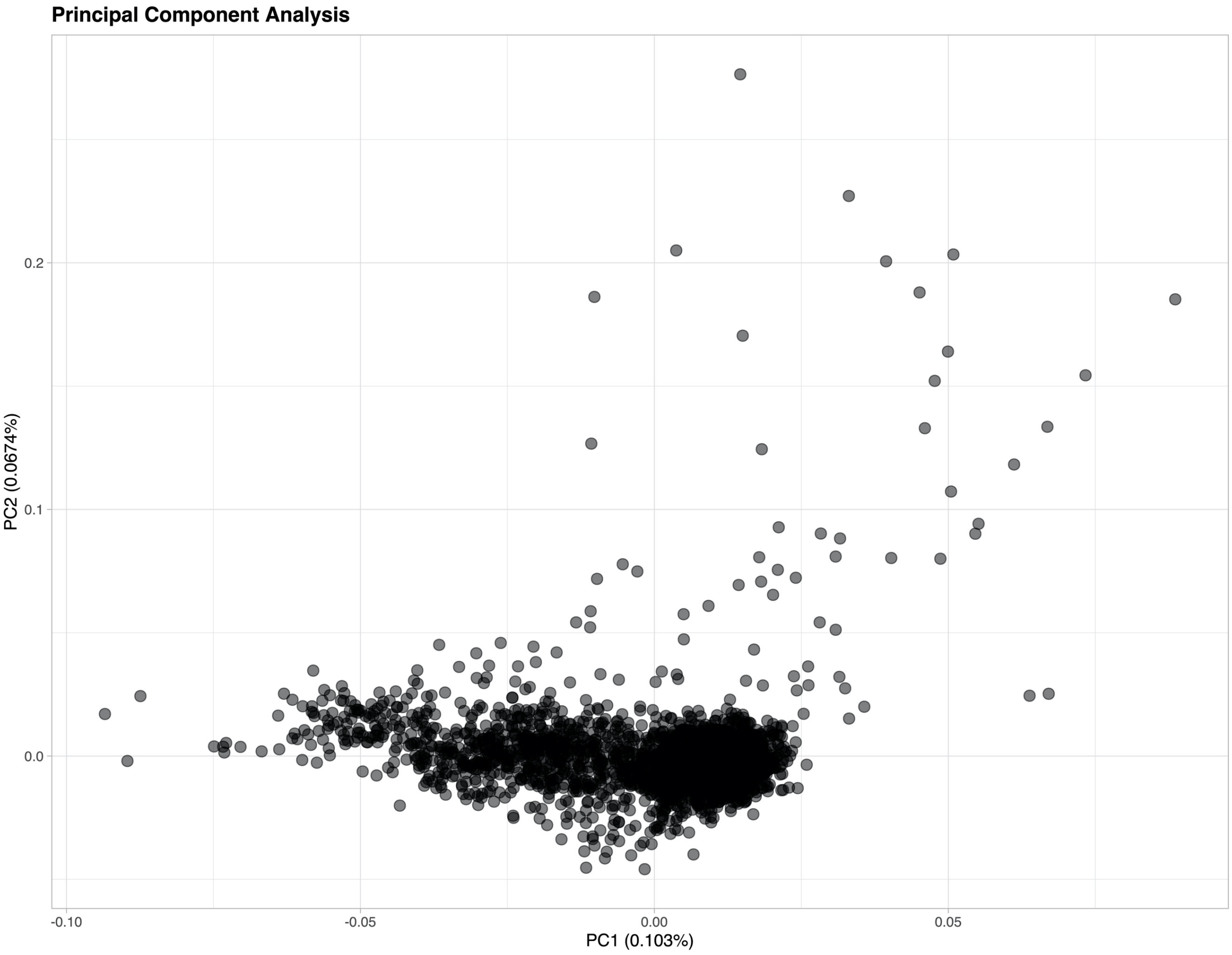

Principal Component Analysis
PC2 (0.0674%)
PC1 (0.103%)
0.2
0.1
0.0
-0.10
-0.05
0.00
0.05
