# Supplementary material for: Investigation of gene-gene interactions in cardiac traits and serum fatty acid levels in the LURIC Health Study
Source: PLoS One. 2020 Sep 11;15(9):e0238304. doi: 10.1371/journal.pone.0238304 (PMC7485803; doi:10.1371/journal.pone.0238304)
Supplement: S3 Fig — Power simulations under the main effect filtering for number of SNP A) cardiac traits in logistic regression and B) fatty acids in linear regression, and for sample size with C) cardiac traits in logistic regression and D) fatty acids in linear regression. Power was estimated at varying beta coefficients under different regression models for sample size and for number of selected SNP for filtering. Each simulation was performed with 1000 replications. The desired power at 80% was labeled with black horizontal line and the sample size at 2824 or the number of selected SNP at 100 was labeled with red vertical. 80% power was reached with 100 SNPs (4,950 pairwise combinations of SNPs) if the beta coefficient is larger than 0.2 in the logistic regression (A) and if the beta coefficient is larger than 0.1 in the linear regression (B). 80% power was reached with 2824 people if the beta coefficient is larger than 0.2 in the logistic regression (C) and if the beta coefficient is larger than 0.1 in the linear regression (D). (PDF) [file pone.0238304.s003.pdf]

S3 Fig. Power simulations under the main effect filtering for number of SNP A) cardiac traits in logistic regression and B) fatty acids in linear regression, and for sample size with C) cardiac traits in logistic regression and D) fatty acids in linear regression. The test power was estimated at different beta coefficients under different regression models for sample size and for number of selected SNP. Each simulation was performed with 1000 replications. The desired power at 0.8 was labeled with black horizontal line and the sample size at 2824 or the number of selected SNP at 100 was labeled with red vertical. The test power could reach 0.8 for the analyses with 100 SNPs (4,950 of pairwise combinations) if the beta coefficient is larger than 0.2 in the logistic regression (A) and if the beta coefficient is larger than 0.1 in the linear regression (B). The test power could reach 0.8 for the analyses with 2824 people if the beta coefficient is larger than 0.2 in the logistic regression (C) and if the beta coefficient is larger than 0.1 in the linear regression (D).

A)

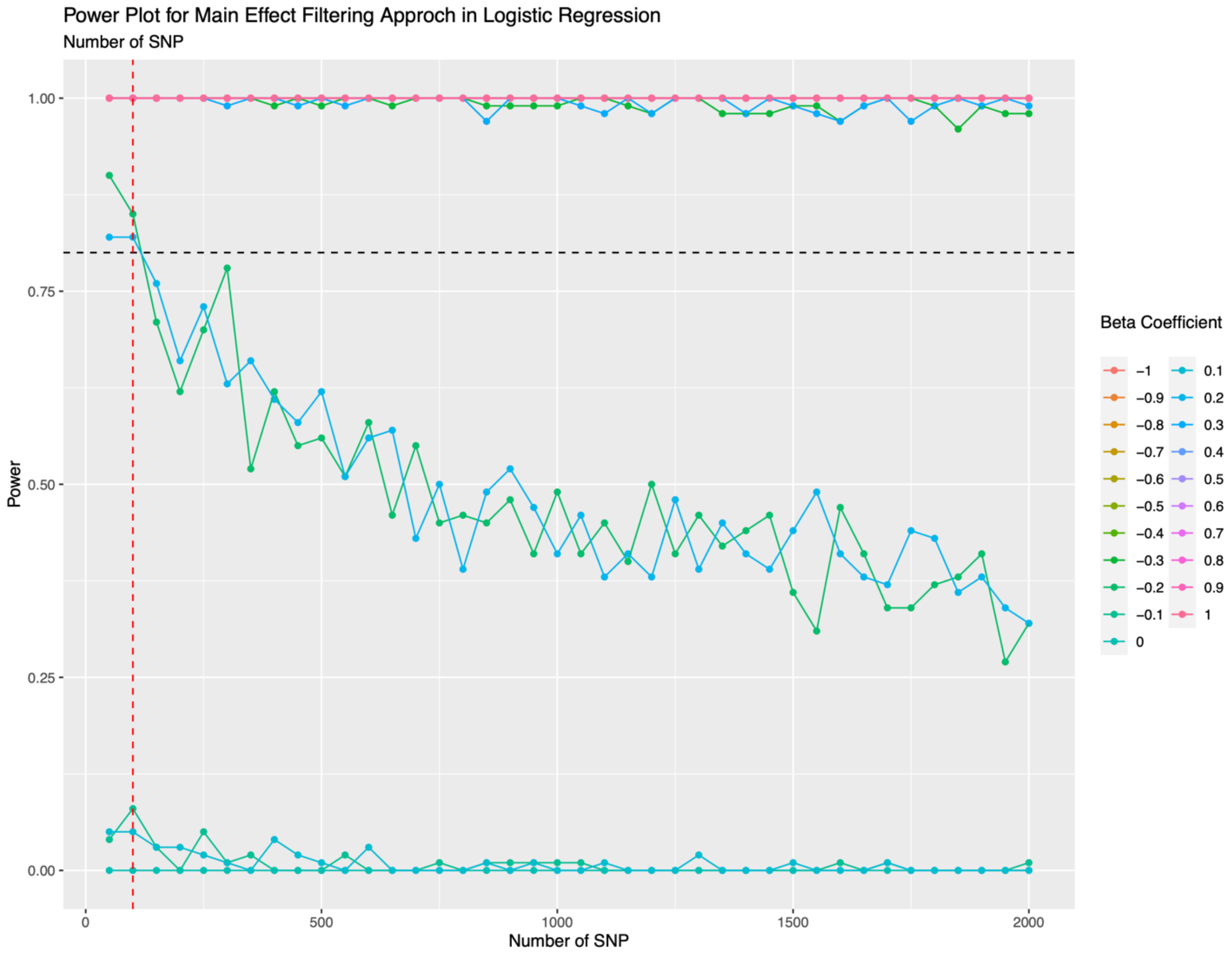

Power Plot for Main Effect Filtering Approch in Logistic Regression
Number of SNP
Power
1.00
0.75
0.50
0.25
0.00
0
500
1000
1500
2000
Number of SNP
Beta Coefficient
−1
−0.9
−0.8
−0.7
−0.6
−0.5
−0.4
−0.3
−0.2
−0.1
0
0.1
0.2
0.3
0.4
0.5
0.6
0.7
0.8
0.9
1

B)

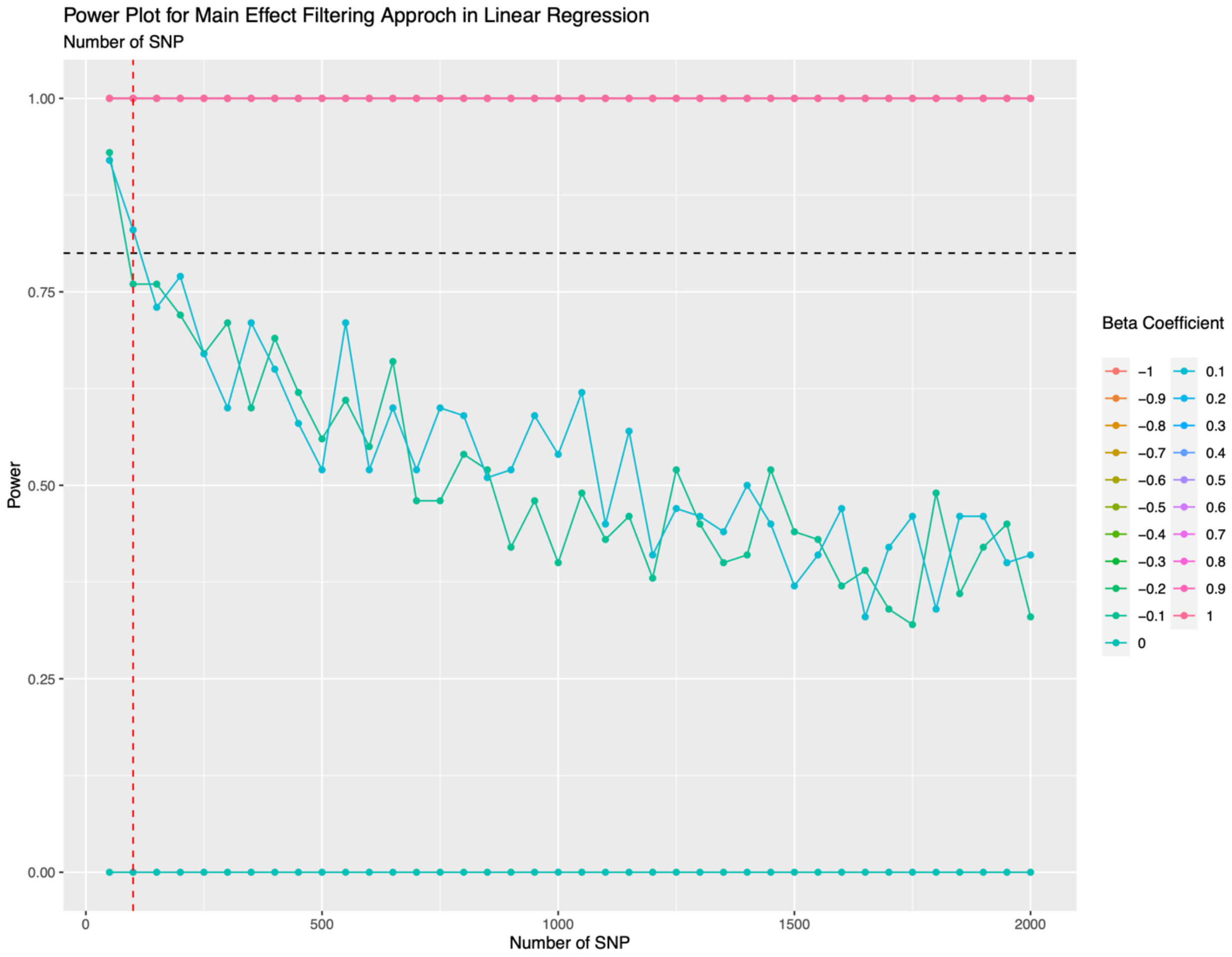

Power Plot for Main Effect Filtering Approch in Linear Regression
Number of SNP
Power
1.00
0.75
0.50
0.25
0.00
0
500
1000
1500
2000
Number of SNP
Beta Coefficient
−1
−0.9
−0.8
−0.7
−0.6
−0.5
−0.4
−0.3
−0.2
−0.1
0
0.1
0.2
0.3
0.4
0.5
0.6
0.7
0.8
0.9
1

C)

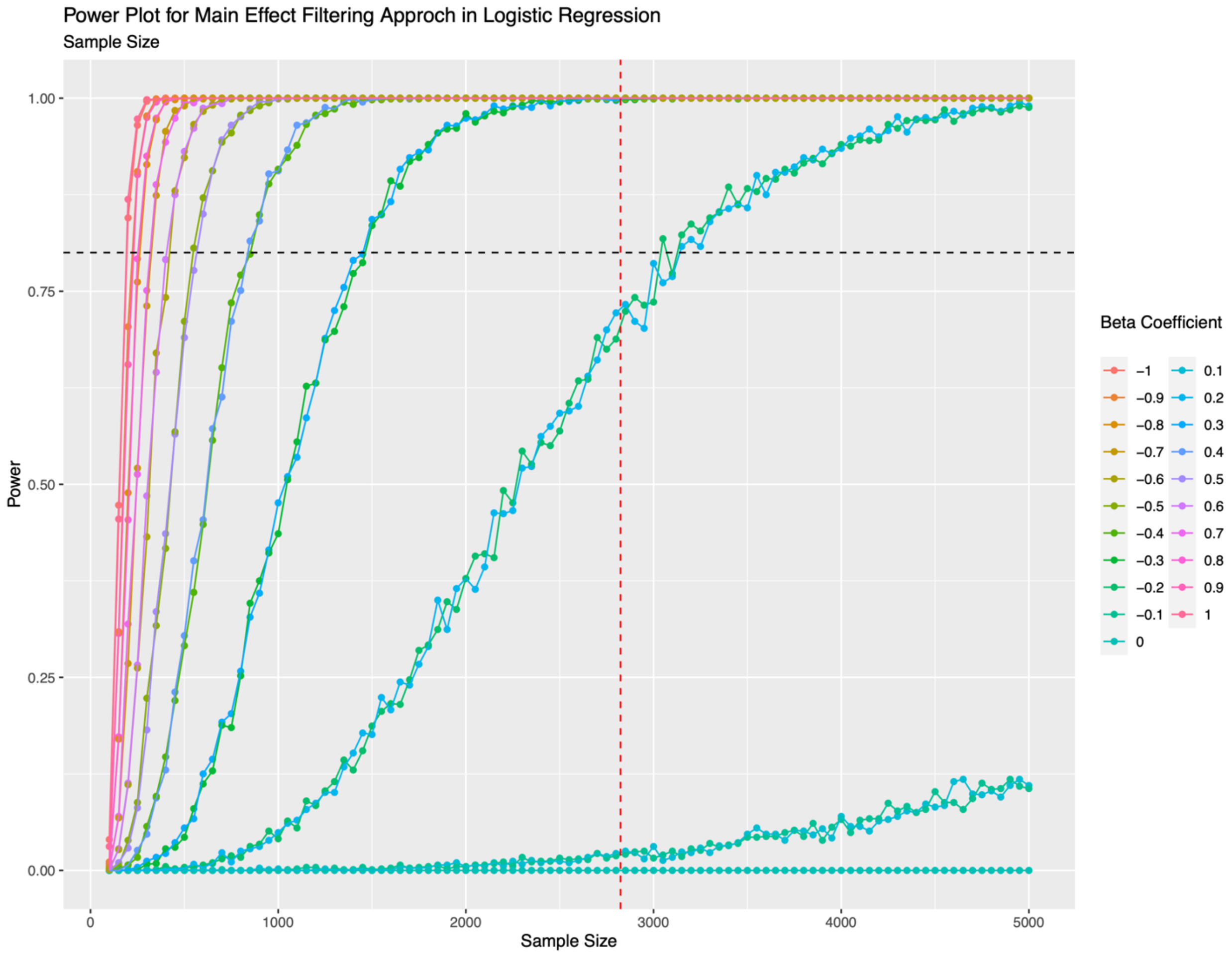

Power Plot for Main Effect Filtering Approch in Logistic Regression
Sample Size
Power
1.00
0.75
0.50
0.25
0.00
0
1000
2000
3000
4000
5000
Sample Size
Beta Coefficient
−1
−0.9
−0.8
−0.7
−0.6
−0.5
−0.4
−0.3
−0.2
−0.1
0
0.1
0.2
0.3
0.4
0.5
0.6
0.7
0.8
0.9
1

D)

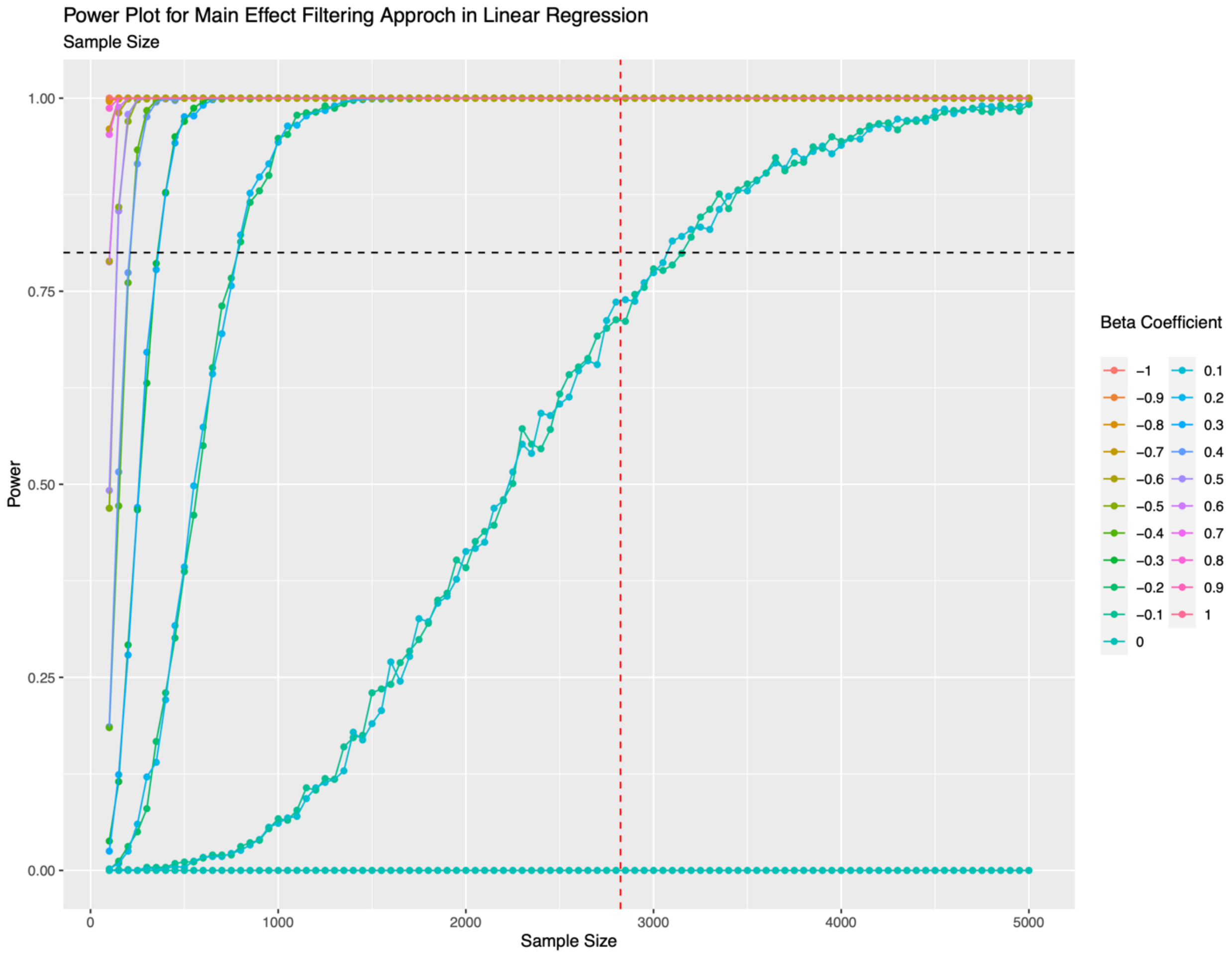
Power Plot for Main Effect Filtering Approch in Linear Regression
Sample Size
Power
1.00
0.75
0.50
0.25
0.00
0
1000
2000
3000
4000
5000
Sample Size
Beta Coefficient
−1
−0.9
−0.8
−0.7
−0.6
−0.5
−0.4
−0.3
−0.2
−0.1
0
0.1
0.2
0.3
0.4
0.5
0.6
0.7
0.8
0.9
1
