# Supplementary material for: Investigation of gene-gene interactions in cardiac traits and serum fatty acid levels in the LURIC Health Study
Source: PLoS One. 2020 Sep 11;15(9):e0238304. doi: 10.1371/journal.pone.0238304 (PMC7485803; doi:10.1371/journal.pone.0238304)
Supplement: S4 Fig — Distribution of observed LRT p-values for the referential G×Gs based on the same set of 1000 random selected SNPs and G×G under two filtering methods to A) cardiac traits and B) fatty acids. G×G based on 1000 random selected SNPs was performed as baseline to understand the inflation (Black). G×G under main effect filtering (Blue) and G×G under Biofilter filtering (Red) were performed and compared with the G×G based on 1000 random selected SNPs for each phenotype. Individual Q-Q plots were generated to visualize the observed LRT p-values to the expected LRT p-values for cardiac traits and fatty acids. Red line represents the ideal estimation of LRT p-values. The corresponded genomic inflation factors were calculated for showing the model inflation (S2 Table). (PDF) [file pone.0238304.s004.pdf]

S2 Table (A). Statistical summary of covariates and results of ANOVA test between covariates and cardiac traits.

Phenotype	Covariates	Median	Mean	Standard Deviation	N	F value	Pr (>F)	Significance[#]
afibtyp	Age	68.0402	66.4148	9.1279	340	5.760	0.0169	*
	Sex	NA	NA	NA		0.097	0.7554	
	Waist-Hip Ratio	0.9672	0.9640	0.0693		0.637	0.4253	
	BMI	26.9896	27.6906	4.2811		0.612	0.4346	
afibyn	Age	63.7663	62.8722	10.5118	2798	48.072	5.08×10^{-12}	***
	Sex	NA	NA	NA		1.204	0.273	
	Waist-Hip Ratio	0.9638	0.9603	0.0780		0.033	0.856	
	BMI	27.0524	27.4561	4.0510		1.834	0.176	
cadyn	Age	63.8005	62.9203	10.5165	2824	130.405	0	***
	Sex	NA	NA	NA		173.573	0	***
	Waist-Hip Ratio	0.9636	0.9602	0.0779		11.892	0.000572	***
	BMI	27.0416	27.4454	4.0442		0.304	0.581144	
canceryn	Age	63.8100	62.9241	10.5205	2821	52.096	6.77×10^{-13}	***
	Sex	NA	NA	NA		0.849	0.3568	
	Waist-Hip Ratio	0.9636	0.9602	0.0780		0.188	0.6650	
	BMI	27.0416	27.4458	4.0415		2.971	0.0849	.
cmpyn	Age	63.8005	62.9203	10.5165	2824	0.757	0.38444	
	Sex	NA	NA	NA		8.161	0.00431	**
	Waist-Hip Ratio	0.9636	0.9602	0.0779		0.142	0.70639	
	BMI	27.0416	27.4454	4.0442		0.051	0.82201	
death2010	Age	63.8005	62.9203	10.5165	2824	339.192	0	***
	Sex	NA	NA	NA		23.660	1.21×10^{-6}	***
	Waist-Hip Ratio	0.9636	0.9602	0.0779		5.962	0.01468	*
	BMI	27.0416	27.4454	4.0442		8.258	0.00409	**
diabetes2010	Age	63.8005	62.9203	10.5165	2824	143.040	0	***
	Sex	NA	NA	NA		2.801	0.0943	.
	Waist-Hip Ratio	0.9636	0.9602	0.0779		46.374	1.19×10^{-11}	***
	BMI	27.0416	27.4454	4.0442		54.682	1.86×10^{-13}	***
dm2yn	Age	63.8005	62.9203	10.5165	2824	90.491	0	***
	Sex	NA	NA	NA		0.754	0.385	
	Waist-Hip Ratio	0.9636	0.9602	0.0779		31.638	2.04×10^{-8}	***
	BMI	27.0416	27.4454	4.0442		25.561	4.56×10^{-7}	***
hyptenyn	Age	63.8005	62.9203	10.5165	2824	102.922	0	***
	Sex	NA	NA	NA		7.648	0.00572	**
	Waist-Hip Ratio	0.9636	0.9602	0.0779		28.003	1.3×10^{-7}	***
	BMI	27.0416	27.4454	4.0442		107.473	0	***
insuthyn	Age	63.8101	62.9310	10.5320	2811	20.553	6.04×10^{-6}	***
	Sex	NA	NA	NA		7.833	0.00517	**
	Waist-Hip Ratio	0.9636	0.9602	0.0778		9.930	0.00164	**
	BMI	27.0416	27.4446	4.0492		19.412	1.09×10^{-5}	***
rhythyn	Age	63.7663	62.8639	10.5348	2789	11.154	0.00085	***
	Sex	NA	NA	NA		4.316	0.03786	*
	Waist-Hip Ratio	0.9636	0.9602	0.0780		0.946	0.33092	
	BMI	27.0538	27.4478	4.0521		0.000	0.98690	
strokeyn	Age	63.8005	62.9203	10.5165	2824	64.557	1.36×10^{-15}	***
	Sex	NA	NA	NA		0.516	0.472	
	Waist-Hip Ratio	0.9636	0.9602	0.0779		0.396	0.529	
	BMI	27.0416	27.4454	4.0442		2.292	0.130	
vdyn	Age	63.8005	62.9203	10.5165	2824	118.539	0	***
	Sex	NA	NA	NA		9.801	0.00176	**
	Waist-Hip Ratio	0.9636	0.9602	0.0779		1.777	0.18267	
	BMI	27.0416	27.4454	4.0442		2.968	0.08503	.
venthrom	Age	63.7991	62.9135	10.5061	2817	15.699	7.61×10^{-5}	***
	Sex	NA	NA	NA		14.305	0.000159	***
	Waist-Hip Ratio	0.9640	0.9603	0.0780		1.574	0.209665	
	BMI	27.0484	27.4511	4.0462		16.687	4.53×10^{-5}	***

[#]Significance codes: '*' - 0.001 / '**' - 0.01 / '*' - 0.05 / '.' - 0.1 / ' ' - 1 / NA - Not Available**

S2 Table (B). Statistical summary of covariates and results of ANOVA test between covariates and fatty acids.

Phenotype and Covariates		Summary Statistics				ANOVA Test		
**Phenotype**	**Covariates**	**Median**	**Mean**	**Standard Deviation**	**N**	**F value**	**Pr (>F)**	**Significance[#]**
	**Palmitic_acid_C16_0**	21.8800	21.9149	1.1415		NA	NA	NA
	Age	63.8101	62.9438	10.5233		0.359	0.549	
**Palmitic_acid_C16_0**	Sex	NA	NA	NA	2776	1.849	0.174	
	Waist-Hip Ratio	0.9636	0.9600	0.0776		0.010	0.922	
	BMI	27.0022	27.4317	4.0350		2.586	0.108	
	**Stearic_acid_C18_0**	17.1600	17.2080	1.1727		NA	NA	NA
	Age	63.8101	62.9438	10.5233		1.772	0.18328	
**Stearic_acid_C18_0**	Sex	NA	NA	NA	2776	14.067	0.00018	***
	Waist-Hip Ratio	0.9636	0.9600	0.0776		10.380	0.00129	**
	BMI	27.0022	27.4317	4.0350		4.831	0.02803	*
	**DHA_C22_6n3**	5.0200	5.1028	1.0893		NA	NA	NA
	Age	63.8101	62.9438	10.5233		118.883	0	***
**DHA_C22_6n3**	Sex	NA	NA	NA	2776	0.000	0.988	
	Waist-Hip Ratio	0.9636	0.9600	0.0776		0.510	0.475	
	BMI	27.0022	27.4317	4.0350		0.549	0.459	
	**LOG_Myristic_acid_C14_0**	0.1989	0.1988	0.1000		NA	NA	NA
	Age	63.8101	62.9438	10.5233		0.079	0.778038	
**LOG_Myristic_acid_C14_0**	Sex	NA	NA	NA	2776	10.923	0.000962	***
	Waist-Hip Ratio	0.9636	0.9600	0.0776		9.313	0.002297	**
	BMI	27.0022	27.4317	4.0350		9.703	0.001858	**
	**LOG_Trans_Palmitoleic_acid_C16_1n7t**	0.1310	0.1402	0.0606		NA	NA	NA
	Age	63.8101	62.9438	10.5233		29.758	5.33×10^{-8}	***
**LOG_Trans_Palmitoleic_acid_C16_1n7t**	Sex	NA	NA	NA	2776	37.874	8.63×10^{-10}	***
	Waist-Hip Ratio	0.9636	0.9600	0.0776		0.785	0.376	
	BMI	27.0022	27.4317	4.0350		27.431	1.75×10^{-7}	***
	**LOG_Oleic_acid_C18_1n9**	2.7619	2.7642	0.0861		NA	NA	NA
	Age	63.8101	62.9438	10.5233		17.469	3.01×10^{-5}	***
**LOG_Oleic_acid_C18_1n9**	Sex	NA	NA	NA	2776	14.514	0.000142	***
	Waist-Hip Ratio	0.9636	0.9600	0.0776		0.379	0.538397	
	BMI	27.0022	27.4317	4.0350		8.440	0.003700	**
	**LOG_C18_2n6tt**	0.0488	0.0592	0.0567		NA	NA	NA
	Age	63.8101	62.9438	10.5233		0.022	0.8819	
**LOG_C18_2n6tt**	Sex	NA	NA	NA	2776	0.326	0.5678	
	Waist-Hip Ratio	0.9636	0.9600	0.0776		3.946	0.0471	*
	BMI	27.0022	27.4317	4.0350		0.156	0.6931	
	**LOG_C18_2n6ct**	0.0198	0.0296	0.0264		NA	NA	NA
	Age	63.8101	62.9438	10.5233		0.221	0.638	
**LOG_C18_2n6ct**	Sex	NA	NA	NA	2776	0.009	0.923	
	Waist-Hip Ratio	0.9636	0.9600	0.0776		0.155	0.694	
	BMI	27.0022	27.4317	4.0350		0.002	0.961	
	**LOG_C18_2n6tc**	0.1133	0.1122	0.0312		NA	NA	NA
	Age	63.8101	62.9438	10.5233		0.090	0.7648	
**LOG_C18_2n6tc**	Sex	NA	NA	NA	2776	30.876	3.01×10^{-8}	***
	Waist-Hip Ratio	0.9636	0.9600	0.0776		0.886	0.3468	
	BMI	27.0022	27.4317	4.0350		5.386	0.0204	*
	**LOG_Linoleic_acid_C18_2n6**	2.4882	2.4915	0.1376		NA	NA	NA
	Age	63.8101	62.9438	10.5233		3.521	0.0607	.
**LOG_Linoleic_acid_C18_2n6**	Sex	NA	NA	NA	2776	15.640	7.85×10^{-5}	***
	Waist-Hip Ratio	0.9636	0.9600	0.0776		42.047	1.05×10^{-10}	***
	BMI	27.0022	27.4317	4.0350		5.294	0.0215	*
	**LOG_a_Linolenic_acid_C18_3n3**	0.1044	0.1107	0.0391		NA	NA	NA
	Age	63.8101	62.9438	10.5233		8.582	0.00342	**
**LOG_a_Linolenic_acid_C18_3n3**	Sex	NA	NA	NA	2776	11.025	0.00091	***
	Waist-Hip Ratio	0.9636	0.9600	0.0776		7.824	0.00519	**
	BMI	27.0022	27.4317	4.0350		0.004	0.95175	
	**Arachidonic_acid_C20_4n6**	16.5400	16.5249	1.5202		NA	NA	NA
	Age	63.8101	62.9438	10.5233		5.189	0.02281	*
**Arachidonic_acid_C20_4n6**	Sex	NA	NA	NA	2776	7.169	0.00746	**
	Waist-Hip Ratio	0.9636	0.9600	0.0776		4.890	0.02709	*
	BMI	27.0022	27.4317	4.0350		0.119	0.72998	
	**LOG_Dihomo_g_Linolenic_C20_3n6**	0.9933	0.1006	0.1357		NA	NA	NA
	Age	63.8101	62.9438	10.5233		4.43	0.0354	*
**LOG_Dihomo_g_Linolenic_C20_3n6**	Sex	NA	NA	NA	2776	15.18	9.99×10^{-5}	***
	Waist-Hip Ratio	0.9636	0.9600	0.0776		0.01	0.9187	
	BMI	27.0022	27.4317	4.0350		26.97	2.22×10^{-7}	***
	**LOG_EPA_C20_5n3**	0.5306	0.5535	0.1595		NA	NA	NA
	Age	63.8101	62.9438	10.5233		0.132	0.7165	
**LOG_EPA_C20_5n3**	Sex	NA	NA	NA	2776	4.134	0.0421	*
	Waist-Hip Ratio	0.9636	0.9600	0.0776		0.456	0.4995	
	BMI	27.0022	27.4317	4.0350		0.283	0.5950	

[#]Significance codes: '*' - 0.001 / '**' - 0.01 / '*' - 0.05 / '.' - 0.1 / ' ' - 1 / NA - Not Available**
