# Supplementary material for: Investigation of gene-gene interactions in cardiac traits and serum fatty acid levels in the LURIC Health Study
Source: PLoS One. 2020 Sep 11;15(9):e0238304. doi: 10.1371/journal.pone.0238304 (PMC7485803; doi:10.1371/journal.pone.0238304)
Supplement: S1 Table — (PDF) [file pone.0238304.s005.pdf]

## S1 Table. Description for phenotypes in cardiac traits and fatty acids with post-QC sample size.

| **Cardiac trait** | **Description** | **Sample Size** |
|---|---|---|
| *afibtyp* | Arrhythmia (1=chronic,2=intermit.) | 340 |
| *afibyn* | Atrial fibrillation (yes/no) | 2798 |
| *cadyn* | Coronary artery disease (>10% (=20% or more)) or clinical(y/n) | 2824 |
| *canceryn* | Cancer disease | 2821 |
| *cmpyn* | Cardiomyopathy (yes/no) | 2824 |
| *death2010* | Yes/No | 2824 |
| *diabetes2010* | Diabetes (definition including hba1c > = 6.5) | 2824 |
| *dm2yn* | Type II diabetes mellitus | 2824 |
| *hyptenyn* | History of arterial hypertension | 2824 |
| *insuthyn* | Treatment with insulin | 2811 |
| *pvdyn* | Peripheral vascular disease | 2824 |
| *rhythyn* | Arrhythmia | 2789 |
| *strokeyn* | Stroke/PRIND/TIA | 2824 |
| *vdyn* | Valve disease (yes/no) | 2824 |
| *venthrom* | Venous thrombosis/pulmonary embolism | 2817 |
| **Fatty Acids** | **Description** | **Sample Size** |
| *Palmitic_acid_C16_0* | Saturated fatty acid | 2776 |
| *Stearic_acid_C18_0* | Saturated fatty acid | 2776 |
| *DHA_C22_6n3* | polyunsaturated omega-3 fatty acid | 2776 |
| *LOG_Myristic_acid_C14_0* | Log-transformed measurement in saturated fatty acid | 2776 |
| *LOG_Trans_Palmitoleic_acid_C16_1n7t* | Log-transformed measurement in exogenous monosaturated fatty acid, trans-palmitoleic acid (C16:1n7t) | 2776 |
| *LOG_Oleic_acid_C18_1n9* | Log-transformed measurement in monounsaturated omega-9 fatty acid | 2776 |
| *LOG_C18_2n6tt* | Log-transformed measurement in trans-fatty acid isomer of C18:2n6, 9-trans 12-trans octadecanoic acid, linolelaidic acid | 2776 |
| *LOG_C18_2n6ct* | Log-transformed measurement in trans-fatty acid isomer of C18:2n6, 9-cis 12-trans octadecanoic acid | 2776 |
| *LOG_C18_2n6tc* | Log-transformed measurement in trans-fatty acid isomer of C18:2n6, 9-trans 12-cis octadecanoic acid | 2776 |
| *LOG_Linoleic_acid_C18_2n6* | Log-transformed measurement in polyunsaturated omega-6 fatty acid | 2776 |
| *LOG_a_Linolenic_acid_C18_3n3* | Log-transformed measurement in polyunsaturated omega-3 fatty acid | 2776 |
| *Arachidonic_acid_C20_4n6* | Polyunsaturated omega-6 fatty acid | 2776 |
| *LOG_Dihomo_g_Linolenic_C20_3n6* | Log-transformed measurement in polyunsaturated omega-6 fatty acid | 2776 |
| *LOG_EPA_C20_5n3* | Log-transformed measurement in polyunsaturated omega-3 fatty acid | 2776 |
