# Supplementary material for: Investigation of gene-gene interactions in cardiac traits and serum fatty acid levels in the LURIC Health Study
Source: PLoS One. 2020 Sep 11;15(9):e0238304. doi: 10.1371/journal.pone.0238304 (PMC7485803; doi:10.1371/journal.pone.0238304)
Supplement: S3 Table — The SE median was reported for the standard median approach (M) and the SE was reported for the linear regression approach (R). (PDF) [file pone.0238304.s007.pdf]

S3 Table. Genomic inflation factor (Lambda) for GxGs from the standard median approach and the linear regression approach for different models. The SE median was reported for the standard median approach (M) and the SE was reported for the linear regression approach (R).

Phenotype	Approach	randomly selected 1000 SNPs		main effect filtering		Biofilter filtering	
		Lambda	SE median/SE	Lambda	SE median/SE	Lambda	SE median/SE
afibtyp	M	1.03821	0.01297616	0.946278	0.1349771	1.072622	0.07178712
	R	1.030303	1.051124×10^{-5}	1.072858	0.0006492925	1.129662	0.0004007088
afibyn	M	1.006215	0.01271232	0.972404	0.1453494	0.949328	0.06048544
	R	1.009363	8.282718×10^{-6}	1.147279	0.002054755	0.9518124	0.000338127
cadyn	M	1.008898	0.01270998	1.039487	0.1246664	1.03251	0.06193636
	R	1.009182	7.123819×10^{-6}	0.9901078	0.0008235546	0.9751615	0.0002615357
canceryn	M	1.004603	0.01261851	0.9259866	0.1201818	0.9622107	0.05628091
	R	1.001896	1.200701×10^{-5}	0.9534782	0.001010342	0.8862285	0.0002157663
cmpyn	M	1.007556	0.01264038	0.9329605	0.1217168	1.118909	0.0635293
	R	1.003653	7.795837×10^{-6}	0.9675241	0.0005634399	0.999694	0.000357618
death2010	M	1.001386	0..01262588	1.004067	0.1296779	0.9224971	0.06559898
	R	1.002506	6.689825×10^{-6}	1.031356	0.000361966	1.032554	0.000325015
diabetes2010	M	1.003532	0.01266436	0.9251811	0.1195487	1.024462	0.06353775
	R	1.005554	8.719343×10^{-6}	0.9495772	0.0007615231	1.000275	0.0002866534
dm2yn	M	1.007288	0.0126762	1.078125	0.1308955	0.977773	0.0673575
	R	1.006496	8.122141×10^{-6}	1.040344	0.0006531178	1.060487	0.0002895953
hyptenyn	M	1.005676	0.01267009	1.095298	0.1478245	1.002456	0.06183031
	R	1.006014	7.245161×10^{-6}	1.147654	0.003651003	0.9738963	0.0001671638
insuthyn	M	1.001924	0.01264147	1.081613	0.1132811	1.021777	0.06493984
	R	1.00374	7.826372×10^{-6}	0.9003065	0.0005778866	1.021992	0.000350687
pvdyn	M	0.9992397	0.01259861	1.07088	0.132036	0.9356438	0.05707506
	R	1.000344	5.370829×10^{-6}	1.049797	0.0005189321	0.8983526	0.0002878237
rhythyn	M	0.9976273	0.01267003	1.08376	0.1324914	1.028752	0.06232874
	R	1.005954	1.652876×10^{-5}	1.052039	0.0009262465	0.9806963	0.0003656504
strokeyn	M	1.012923	0.01274881	0.9308163	0.8849962	1.029829	0.06587682
	R	1.012262	7.96484×10^{-6}	0.8849962	0.0008690248	1.037805	0.0001164889
vdyn	M	1.003532	0.01271096	0.9224972	0.1214283	0.8902999	0.06192187
	R	1.009263	6.335183×10^{-6}	0.9649543	0.000650852	0.9738736	0.0004169195
venthrom	M	0.9992397	0.01256166	1.06082	0.1370548	1.051295	0.06515
	R	0.9973834	1.174052×10^{-5}	1.073865	0.002685949	1.024733	0.0004278363
Palmitic_acid_C16_0	M	0.9974154	0.01257398	0.9569238	0.1362133	0.9741287	0.06111027
	R	0.9983656	1.096399×10^{-5}	1.07936	0.001371742	0.9617847	0.0003208915
Stearic_acid_C18_0	M	0.9989483	0.0126399	1.017109	0.1286044	0.9410496	0.06194214
	R	1.003606	9.799617×10^{-6}	1.020711	0.0009997651	0.9749018	0.0003214398
DHA_C22_6n3	M	0.997979	0.01256766	1.029364	0.1274316	1.08682	0.06353865
	R	0.9978818	7.050964×10^{-6}	1.012895	0.000608883	0.9989879	0.000463686
LOG_Myristic_acid_C14_0	M	1.000259	0.0126255	1.132384	0.127516	0.9996175	0.06224137
	R	1.002477	6.4029×10^{-6}	1.012681	0.0008565434	0.9799912	0.0002575449
LOG_Trans_Palmitoleic_acid_C16_1n7t	M	0.9979254	0.01262856	1.24908	0.1591406	1.033404	0.06549707
	R	1.002696	1.158728×10^{-5}	1.259859	0.00178002	1.031354	0.0002504775
LOG_Oleic_acid_C18_1n9	M	1.002286	0.01260121	1.115227	0.1235292	1.070394	0.0581319
	R	1.000543	7.7862×10^{-6}	0.9809867	0.0008374728	0.9144163	0.0003731743
LOG_C18_2n6tt	M	0.997299	0.01290081	2.769811	0.3435913	0.9787235	0.0670423
	R	1.023997	3.786269×10^{-5}	2.731436	0.001505836	1.055543	0.0002848083
LOG_C18_2n6ct	M	1.005575	0.0128199	1.750307	0.2127019	0.998904	0.06164679
	R	1.017822	2.021909×10^{-5}	1.686578	0.001955953	0.9703363	0.0003067925
LOG_C18_2n6tc	M	1.011163	0.01269489	1.111404	0.1316418	0.07093839	0.07093839
	R	1.007968	1.067733×10^{-5}	1.043999	0.001180289	1.114916	0.000561429
LOG_Linoleic_acid_C18_2n6	M	0.9945591	0.01261709	0.9894061	0.1125458	1.138842	0.06240026
	R	1.001805	7.473811×10^{-6}	0.8942404	0.0006402555	0.9822347	0.0003042456
LOG_a_Linolenic_acid_C18_3n3	M	0.9985544	0.01263681	1.189382	0.1518738	0.9362355	0.05594655
	R	1.003348	1.205633×10^{-5}	1.206297	0.0009772376	0.8809484	0.000217603
Arachidonic_acid_C20_4n6	M	1.001417	0.01264381	1.023702	0.118707	1.010514	0.06629753
	R	1.003926	7.616915×10^{-6}	0.9421388	0.0009266119	1.043557	0.0003271516
LOG_Dihomo_g_Linolenic_C20_3n6	M	0.9961191	0.01264797	1.068671	0.1325154	0.9293836	0.064759
	R	1.004254	8.289096×10^{-6}	1.05358	0.0005324368	1.018875	0.0003880255
LOG_EPA_C20_5n3	M	1.002762	0.01258748	1.105038	0.1356612	0.9664827	0.06673365
	R	0.9994536	7.648535×10^{-6}	1.077219	0.0009458766	1.050984	0.0002193462
