# Supplementary material for: Investigation of gene-gene interactions in cardiac traits and serum fatty acid levels in the LURIC Health Study
Source: PLoS One. 2020 Sep 11;15(9):e0238304. doi: 10.1371/journal.pone.0238304 (PMC7485803; doi:10.1371/journal.pone.0238304)
Supplement: S4 Table — All significant gene-gene pairs for specific phenotypes were recorded with the SNP pairs, genes, p-values from GWAS, sample size, number of cases, beta coefficients, LRT p-values, FDR and Bonferroni-adjusted LRT p-values, and other information. Interactions for overall FDR and Bonferroni-adjusted LRT p-values less than 0.05 are highlighted in yellow, and interactions for only overall FDR-adjusted LRT p-value is less than 0.05 are highlighted in blue. The beginning of a new phenotype is highlighted in blue. Genes which were annotated by Biofilter with a 5kb boundary are highlighted in brown. Genes which were annotated by Biofilter with a 5kb boundary are highlighted in brown. (PDF) [file pone.0238304.s008.pdf]

S4 Table. Summary for significant interactions among the cardiac traits and fatty acids under two filtering methods based on the PLATO output. All significant gene-gene pairs for specific phenotypes were recorded with the SNP pairs, genes, p-values from GWAS, sample size, number of cases, beta coefficients, LRT p-values, FDR and Bonferroni-adjusted LRT p-values, and other information. Interactions for overall FDR and Bonferroni-adjusted LRT p-values less than 0.05 are highlighted in yellow, and interactions for only overall FDR-adjusted LRT p-value is less than 0.05 are highlighted in blue. The beginning of a new phenotype is highlighted in blue. Genes which were annotated by Biofilter with a 5kb boundary are highlighted in brown.

(URL: https://docs.google.com/spreadsheets/d/100EAqrKBRj3sjIHU4K7XeoIGRUY3zVU8xkuIlzXtITY/edit?usp=sharing)
