# Supplementary material for: Investigation of gene-gene interactions in cardiac traits and serum fatty acid levels in the LURIC Health Study
Source: PLoS One. 2020 Sep 11;15(9):e0238304. doi: 10.1371/journal.pone.0238304 (PMC7485803; doi:10.1371/journal.pone.0238304)
Supplement: S1 File — (PDF) [file pone.0238304.s009.pdf]

S1 File. Raw GWAS results for each phenotype. All 29 raw GWAS results could be accessed at URL: https://drive.google.com/drive/folders/12ByaBModrOJW_8z2YS_V3Zuc5heIgDzJ?usp=sharing. The explanations for headers could be found at the end of PLATO manuscript at https://ritchielab.org/files/RL_software/plato-manual-2.1.pdf.
